# Nonlinear multimode photonics on-chip

**DOI:** 10.1515/nanoph-2025-0105

**Published:** 2025-06-27

**Authors:** Valerio Vitali, Thalía Domínguez Bucio, Hao Liu, Jack Haines, Pooja Uday Naik, Massimiliano Guasoni, Frederic Gardes, Lorenzo Pavesi, Ilaria Cristiani, Cosimo Lacava, Periklis Petropoulos

**Affiliations:** Electrical, Computer and Biomedical Engineering Department, University of Pavia, Pavia, 27100, Italy; Optoelectronics Research Centre, University of Southampton, Southampton, SO17 1BJ, UK; Nanoscience Laboratory, Department of Physics, University of Trento, Trento, 38123, Italy

**Keywords:** nonlinear optics, integrated waveguides, multimode waveguides, four-wave mixing, optical signal processing, quantum optics

## Abstract

Nonlinear integrated photonics, which takes advantage of the strong field enhancement in integrated waveguides to boost the efficiency of nonlinear effects, has paved the way for the demonstration of cutting-edge applications. These achievements have also been made possible by the impressive progress in material engineering and fabrication processes, which have enabled a remarkable control of the nonlinear dynamics in the waveguides. While researchers initially focused their attention on single-mode devices, in recent years, the exploitation of nonlinear effects in integrated multimode waveguides has attracted significant interest. Indeed, the simultaneous use of different spatial modes of the same multimode waveguide has opened new avenues in the realization of integrated nonlinear processors, thanks to the ability to tune the dispersion profiles of the different modes. In this review, we discuss the most recent advances in nonlinear multimode photonics on-chip. In the first part, we review the use of intermodal nonlinear effects for frequency generation. The use of intermodal nonlinear effects has been extensively reported, for example, for wavelength conversion for telecom applications, generation of photon pair sources for quantum optics and mid-infrared frequency generation. Then, we discuss several demonstrations of nonlinear multimode waveguides used to perform simultaneous multi-channel and multi-functional optical signal processing, such as nonlinear switching and logic operations. Next, supercontinuum generation in nonlinear multimode waveguides will be discussed. Finally, we report the use of high-quality-factor micro-resonators based on multimode waveguides for the realization of compact and widely-tunable integrated Raman lasers and optical frequency comb sources with record-low threshold power.

## 1 Introduction

For over half a century, nonlinear optics has continued to evolve and influence numerous areas of science and technology thanks to the opportunities it offers in tailoring the properties of propagating waves and controlling light–light and light–matter interactions. In this context, optical fibers have played a pivotal role since the early days of nonlinear optics, initially serving as a novel medium for investigating nonlinear optical effects, to the current time, where the effects of optical nonlinearities influence directly the design of transmission systems [1], [2]. However, the relatively low nonlinear coefficient of silica optical fibers poses some challenges and limitations in the implementation of advanced nonlinear devices and typically requires the use of high optical powers and/or long fiber lengths [3]. The large footprint of optical fiber-based nonlinear devices hinders their adoption in scale in applications that require a large number of nonlinear elements, and, to a degree, their cost reduction. Over the last two decades, the implementation of nonlinear processes on small-footprint integrated chips, pushed by the dominant trend in research and development towards device integration and system miniaturization, gained a central role in the nonlinear optics research community. Nonlinear integrated photonics leverages the strong field enhancement in integrated waveguides and resonators and the use of material platforms with nonlinearity coefficients that are orders of magnitude higher than that of silica glass to boost the efficiency of nonlinear effects [4], [5]. These properties have paved the way for the realization of devices and applications covering a wide range of research fields such as all-optical signal processing for optical communications [6], [7], quantum information processing [8], [9], [10] and spectroscopy [11], [12], just to name a few. These advancements were also enabled by the significant progress in material engineering and fabrication techniques in recent years, which have provided exceptional control over waveguide dispersion and consequently the nonlinear dynamics within the waveguides. While the research community initially focused its efforts on the realization of efficient single-mode devices, i.e., where the waveguide is designed to operate with just one waveguide spatial mode, commonly the fundamental mode, more recently, the exploitation of nonlinear processes in integrated multimode waveguides has been attracting increasing attention.

To enhance the accessibility of the review, we briefly recall here the key concepts underpinning nonlinear multimode photonics. In multimode waveguides, light propagates in distinct spatial modes, each with its own dispersion and field distribution. When combined with optical nonlinearities, this enables intermodal interactions that expand the design space for phase matching, spectral translation, and spatial control. Foundational theoretical models, such as the coupled-mode equations and nonlinear Schrödinger formalism, are thoroughly treated in Agrawal’s textbook on nonlinear fiber optics [2]. While originally developed in the context of optical fibers, these models are universal and directly applicable to integrated platforms, with appropriate adaptations for geometry and material dispersion. A complementary and broader perspective on multimode nonlinear dynamics, including both fiber-based and integrated systems, is provided by the tutorial by Wright et al. [13]. Indeed, the simultaneous use of distinct spatial modes in the same multimode waveguide offers more flexibility in the device design and allows the control of light in new and richer ways [13]. Multimodality can be considered as an additional degree of freedom in the design, and one that can be exploited to further expand the range of applications of nonlinear optics and build more complex and sophisticated components [14], [15].

In addition to the complex manipulation of optical spatial modes for the realization of nonlinear processors, multimode waveguides and fibers have been exploited to increase the capacity of fiber optic communication systems. This is achieved through mode-division-multiplexing (MDM), which is a type of space-division-multiplexing (SDM), where data transmission capacity is increased through the use of several, parallel mode-channels [16], [17]. MDM represents a valuable technique to overcome the so-called ‘capacity-crunch’ of optical communication systems traditionally based on single-mode fibers and devices [18]. However, it introduces unprecedented technical challenges in both system design and implementation due to the need to accurately handle and manipulate different spatial modes, as well as the linear and nonlinear interactions between them. The same considerations also apply to nonlinear multimode photonics on-chip, which has lagged behind its single-mode counterpart due to the greater complexity in design, simulation and implementation. Indeed, the impressive results that have been reported in recent years based on the use of nonlinear effects in integrated multimode waveguides and resonators have also been achieved thanks to the huge progress in the design and realization of linear devices for effective mode manipulation, such as multimode-interference couplers (MMIs) [19], [20], [21], mode converters, multiplexers and de-multiplexers [22], [23], [24], [25], optical power splitters for arbitrary guided modes [26], [27], multimode waveguide crossing [28], [29] and bending [30], [31].

The use of distinct spatial modes in nonlinear multimode waveguides can also add functionalities which might be either challenging or complex to implement in single-mode nonlinear photonics. For example, stimulated intermodal Brillouin scattering in multimode waveguides was exploited to realize circulator-free Brillouin integrated circuits, eliminating the need for additional non-reciprocal components, such as circulators, and fine spectral filtering to separate pump and signal waves, in contrast with previous demonstrations of Brillouin scattering [32], [33]. By leveraging intermodal Brillouin energy transfer, flexible on-chip light sources, optical amplifiers, and non-reciprocal devices could be realized. Moreover, stimulated intermodal Brillouin scattering represents a form of active mode conversion, with potential applications in active optical switching, power routing, and on-chip MDM [32], [33], [34].

Ultimately, a new class of photonic technologies can be achieved with multi-mode waveguides, that are fundamentally unattainable with traditional single-mode devices. In particular, the ability to achieve nonlinear phase matching across multiple distinct pairs of modes allows for the development of ultra-broadband and highly reconfigurable optical sources. By selectively pumping different modal combinations, light can be generated and/or amplified across multiple spectral bands that are widely separated and even concatenated, limited only by the material transparency window. Crucially, this does not require anomalous dispersion near the pump, as in single-mode systems. A single compact integrated multimode waveguide can thus enable access to spectral regions in the visible and mid-infrared (MIR) even beyond the reach of conventional sources such as Erbium doped fiber amplifiers (EDFAs) or quantum cascade lasers (QCLs), with clear benefits for broadband communications and sensing. Moreover, the generated or amplified light may exhibit a rich spatial structure, enabling new functionalities in spatial multiplexing, imaging, and multidimensional signal processing.

Beyond their practical relevance, nonlinear systems involving coupling across *N* spatial modes offer, from a theoretical perspective, an exceptionally fertile platform for exploring novel complex nonlinear dynamics. It is precisely in *N*-dimensional modal spaces that such dynamics emerge clearly and distinctively, whereas they remain absent or ambiguous in simpler configurations involving only one or two modes. Paradigmatic examples include the concept of multidimensional modal attraction, rejection and control [35], [36], as well as multidimensional modulational instability [37], thermalization [38], and spatial condensation phenomena such as beam self-cleaning [39]. While several of these effects were initially demonstrated in optical fibers, they are in fact adaptable to any type of multimode waveguide, including integrated platforms, where they may be potentially richer and more accessible due to the inherently stronger material nonlinearities.

In this paper, a review is given of recent progress in nonlinear multimode photonics in integrated waveguides and resonators. In Section 2, we give a review of the use of intermodal nonlinear processes for frequency generation for different applications, including wavelength conversion for telecom applications in Section 2.1, generation of photon pair sources for quantum applications in Section 2.2 and MIR frequency generation for spectroscopy applications in Section 2.3. In Section 3, we give a review of several applications of nonlinear multimode photonics to perform simultaneous multi-channel and multi-functional optical signal processing on-chip. Supercontinuum (SC) generation in nonlinear multimode waveguides is then reviewed in Section 4, reporting the use of distinct spatial modes of the same nonlinear waveguide to achieve on-chip spectral control of the SC spectrum and, in addition, the generation of intermodal dispersive waves (DWs), which could be exploited to extend the spectral bandwidth of the SC generation process. In Section 5, we review the use of high-quality-factor micro-resonators based on multimode waveguides, which have been recently utilized to demonstrate compact and widely-tunable integrated Raman lasers and optical frequency comb sources with record-low threshold power. Finally, concluding remarks and future perspectives on this exciting and rapidly growing research topic are presented in the Conclusions section.

## 2 Intermodal nonlinear processes for frequency generation

All-optical frequency generation represents an enabling operation for many applications spanning optical communications, spectroscopy and quantum technologies. Nonlinear integrated optical devices have been widely utilized to generate and convert wavelength components using well-studied parametric optical processes, such as those based on second-order-nonlinearities (*χ* ^(2)^) [40] like second harmonic generation (SHG), sum frequency generation (SFG) and difference frequency generation (DFG), as well as those based on third-order-nonlinearities (*χ* ^(3)^) [41] like four-wave mixing (FWM). In general, the majority of these demonstrations have exploited intramodal nonlinear processes, i.e., where all of the involved waves propagate in the same optical spatial mode of the integrated waveguide. In contrast, in this review, we focus on the simultaneous use of different spatial modes in nonlinear multimode waveguides, which enhances design flexibility and provides additional tools for dispersion engineering, as required in order to satisfy phase-matching and enable efficient and broadband frequency generation. This greater flexibility in design compared to the case of single-mode nonlinear devices results from the possibility to precisely tune the properties of different spatial modes over the wavelengths of interest. In the following sub-sections, the use of intermodal nonlinear processes will be discussed for wavelength conversion in telecommunication systems, generation of photon pair sources for quantum applications and MIR frequency generation. We will present the most recent results in these fields and discuss future research directions.

### 2.1 Wavelength conversion for telecommunication applications

MDM has been studied extensively in communications as a means of addressing the saturation in the capacity of transmission systems. However, while MDM is still under development and its use still lags behind compared to the universal adoption of single-mode wavelength division multiplexing (WDM), its application in all-optical signal processing has motivated innovative research and interesting on-chip demonstrations have been reported such as, for example, all-optical mode-selective wavelength conversion [42], [43], [44]. In these works, directional coupler (DC)-based multiplexer/de-multiplexer devices were implemented on-chip to handle the different spatial modes. Mode-selective wavelength conversion was demonstrated by designing the nonlinear multimode waveguide to satisfy the phase-matching condition for the intramodal degenerate FWM process while suppressing intermodal nonlinearities. Using these implementations, all-optical wavelength conversion of optical signals with advanced modulation formats was demonstrated, e.g. the transmission of an optical orthogonal frequency-division multiplexing (OFDM) signal with quadrature phase shift keying (QPSK) format at 102.6 Gb/s [44].

While solutions based on MDM techniques show great potential, a complementary attractive approach to enhancing optical network capacity arises from recognizing that current telecommunication systems exploit only a small portion of the low-loss bandwidth of silica optical fibers. In this context, system configurations that make use of optical wavelength bands outside the conventional C-band spectrum (1,530–1,565 nm) are currently under investigation [45], [46], [47], [48], [49]. In these next-generation wideband optical communication systems, the capability to generate, convert and manipulate optical signals with complex modulation formats in unconventional wavelengths is an enabling feature. In particular, all-optical wavelength conversion represents a fundamental technology for routing and handling optical wavelengths in WDM systems since it can resolve resource contention and potentially reduce the device complexity of transmitters and receivers, which is particularly critical in systems operating across different optical communication bands [50], [51], [52]. Many demonstrations based on intramodal FWM in integrated waveguides have already been reported in the literature showing the possibility of realizing wavelength conversion across an extended spectral region covering the communication wavelengths [5], [53], [54], [55], [56], [57]. As briefly discussed earlier, intermodal FWM nonlinear effects significantly benefit the realization of broadband wavelength converters thanks to the additional degree of freedom in the design given by the multimodality and the possibility of exploiting the simultaneous mode conversion in SDM systems.

Another advantage of intermodal FWM for wavelength conversion in telecom systems is that it does not require to operate in the anomalous group velocity dispersion (GVD) regime, which is typical, instead, of intramodal FWM-based systems. In the latter case, due to the shorter length scale of integrated waveguides compared to fiber-based devices, a variety of nonlinear effects can occur simultaneously, which, in turn, can result in undesired crosstalk if WDM signals are to be processed. On the contrary, intermodal FWM-based devices can be engineered to suppress concurrent nonlinear effects by properly designing the nonlinear multimode waveguide and the higher-order mode interaction to satisfy only the phase-matching condition for the desired mode and wavelength components.

Depending on the selected optical spatial modes, the waveguide cross-section and, of course, material dispersion, discrete phase-matched bands can be easily controlled and achieved even hundreds of nanometers away from the optical pumps. In addition, because phase-matching with higher-order modes is not achieved by exploiting the fourth-order dispersion coefficient *β*_4_ of the GVD, the phase-matching wavelengths are more tolerant to fabrication deviations compared to the case where the discrete-band phase-matching is obtained by compensating higher-order GVD terms [58]. Multimode waveguides offer improved fabrication tolerances also due to their larger size compared to typical single-mode waveguides. However, the generally larger effective mode area of nonlinear multimode waveguides and the limited mode field overlap between the different interacting spatial modes negatively affect the overall efficiency of the intermodal processes, which is typically lower compared to the intramodal case. Among the different types of intermodal nonlinear processes, phase-conjugation (PC) and Bragg scattering (BS) intermodal FWM have been mainly exploited in integrated multimode waveguides. PC FWM is an amplification process where one degenerate pump (degenerate FWM) or two separate pumps (non-degenerate FWM) amplify the signal and simultaneously generate a new idler component, while BS FWM is an energy exchange process in which the two optical pumps induce the transfer of photons from the signal to the idler [59], [60]. Both nonlinear processes are ruled by the laws of energy and momentum conservation, the latter giving rise to the so-called phase-matching condition. In the most generic case of a non-degenerate FWM process, the energy conservation relation and the linear phase-mismatch Δ*β* for the PC FWM process are given by Eqs. (1) and (2), respectively:

(1) $$\omega_{P1}+\omega_{P2}-\omega_{S}-\omega_{I}=0$$

(2) $$\Delta \beta =\beta^{a}\left(\omega_{P1}\right)+\beta^{b}\left(\omega_{P2}\right)-\beta^{c}\left(\omega_{S}\right)-\beta^{d}\left(\omega_{I}\right)$$

where *ω*_*P*1_, *ω*_*P*2_, *ω*_*S*_ and *ω*_*I*_ are the angular frequencies of the pump 1, pump 2, signal and idler waves, respectively, while *β* ^*a*^, *β* ^*b*^, *β* ^*c*^ and *β* ^*d*^ are the propagation constants corresponding to the modal combination {*a*, *b*, *c*, *d*} [58]. This corresponds to the most general configuration where the two pump photons are in the *a*-th and *b*-th order modes, respectively, the signal photon in the *c*-th order mode and the idler photon in the *d*-th order mode. Unlike the PC FWM process, the BS FWM process allows in principle, the generation of both blue and red shifted copies (*I*_*BS*,*b*_ and *I*_*BS*,*r*_ idler components, at a shorter and longer wavelength, respectively, compared to the signal wavelength) of the seeding signal via a scattering mechanism induced by an intensity grating resulting from the interference of the two pump beams [61]. The two idler frequencies are governed by the energy conservation principle, i.e., they are equal to *ω*_*S*_ ± Δ*ω*, where Δ*ω* is the frequency difference between the two pumps. Efficient energy transfer can generally be achieved only for one idler component at a time by satisfying the phase matching condition, and, in most situations, the other non-phase-matched idler component represents an undesired by-product of the conversion process [61], [62]. Considering, for example, the red BS idler component, the energy conservation relation and the linear phase-mismatch Δ*β* are given by Eqs. (3) and (4), respectively [61]:

(3) $$-\omega_{P1}+\omega_{P2}+\omega_{S}-\omega_{BS,r}=0$$

(4) $$\Delta \beta =-\beta^{a}\left(\omega_{P1}\right)+\beta^{b}\left(\omega_{P2}\right)+\beta^{c}\left(\omega_{S}\right)-\beta^{d}\left(\omega_{BS,r}\right)$$

where *ω*_*BS*,*r*_ is the angular frequency of the red BS idler and, in this configuration, the frequency of pump 1 is greater than that of pump 2.

**Figure 1:**
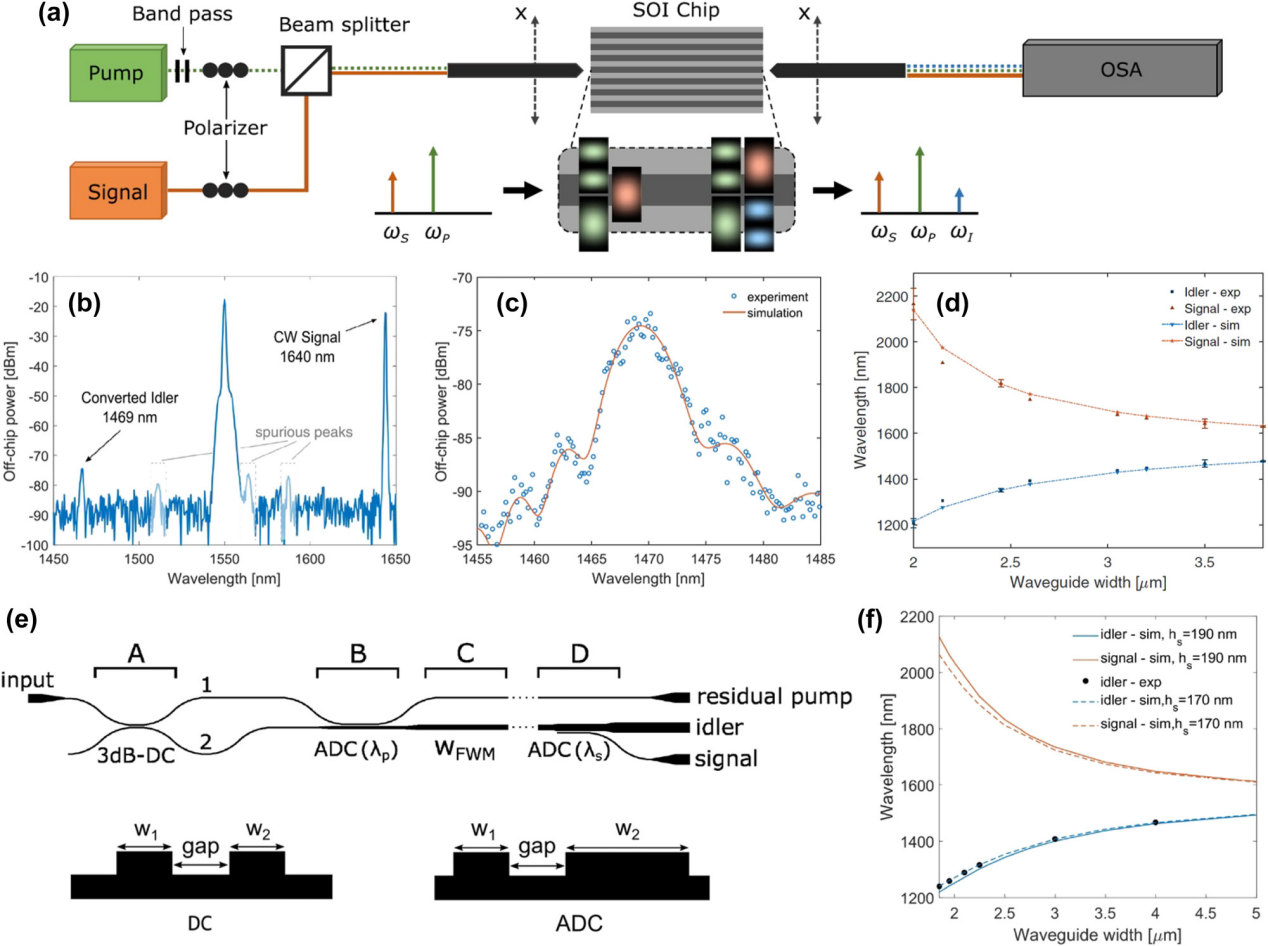
Spontaneous and stimulated intermodal FWM experiments performed at telecommunication wavelengths. (a) Experimental setup used in the spontaneous (with no seed signal source) and stimulated intermodal FWM experiments reported in ref. [58]; (b) example of an acquired optical spectrum and (c) spectral characterization of the generated idler obtained by scanning the signal wavelength in the stimulated intermodal FWM experiments performed on a 3.5 μm wide × 243 nm high multimode waveguide; (d) results of the spontaneous intermodal FWM measurements carried out on multimode waveguides with different widths. (e) Schematic layout of the chip used for the intermodal FWM experiments reported in ref. [63] and (f) results of the spontaneous intermodal FWM experiments performed on multimode waveguides with different widths. (a)–(d) Panels are reproduced with permission from ref. [58] ©2018 Chinese Laser Press. (e)–(f) Panels are adapted from ref. [63] under a CC BY 4.0 license.

The use of the PC intermodal FWM process on an integrated platform was demonstrated for the first time by Signorini et al. in silicon waveguides [58]. The authors extensively investigated a specific modal combination involving the optical pump on both the first and second horizontal transverse electric (TE) modes (TE_00_ and TE_10_, respectively) and the signal and idler on the TE_10_ and TE_00_ modes, respectively. The main experimental results obtained using this configuration are reported in Figure 1. In addition, they also reported other intermodal FWM combinations, involving up to the third-order mode in the TE polarization and also in the transverse magnetic (TM) polarization. The simultaneous excitation of the fundamental and higher-order modes in the nonlinear multimode waveguides was achieved by properly illuminating the input facet of the waveguide with a tapered lensed fiber placed in an offset position relative to the waveguide center and the same coupling technique was also used to collect the signal at the output of the waveguide. Both spontaneous and stimulated intermodal FWM experiments were performed using the setup reported in Figure 1(a), showing the input and output fibers mounted on a piezo-controlled translation stage to excite/collect the waveguide modes of interest. A pulsed pump beam at a fixed wavelength of 1,550 nm and a tunable continuous wave (CW) seed signal were used in the stimulated FWM experiments, while no seed signal was required in the spontaneous FWM case.

Figure 1(b) and (c) reports the results of the stimulated FWM experiments performed on a 3.5 μm wide × 243 nm high multimode waveguide, with an example of an acquired spectrum in panel (b) and the spectral characterization of the stimulated intermodal FWM process in panel (c), together with numerical simulations. It was possible to tune the phase-matching spectral position by varying the effective index of the modes involved in the intermodal FWM process, i.e., by changing the waveguide cross-section. Figure 1(d) reports the results of the spontaneous intermodal FWM experiments performed on nonlinear waveguides with different widths ranging from 2 to 3.8 μm. As can be seen, broad tunability of the phase-matched wavelength position was demonstrated, along with the capability to achieve an extremely wide separation between the signal and the idler. In a later work, Signorini et al. [63] performed similar experiments with the same intermodal FWM combination but with a more advanced integrated design where the different modes were handled directly on-chip. Figure 1(e) shows the layout of this implemented device, in which a 3 dB DC used as a power splitter and an asymmetric directional coupler (ADC) used as a mode converter, in stages A and B, respectively, were employed to excite the proper mode combination in the nonlinear multimode waveguide (stage C). A second ADC was then used in the mode converter and de-multiplexer section (stage D) to divide the different waves, which were individually collected at the output section. Using this device, spontaneous intermodal FWM experiments were carried out using a pulsed pump at 1,550 nm, with the resulting wavelengths of the generated idler and signal waves reported in Figure 1(f) for different multimode waveguide widths. As in the previous work, an impressive tunability of the phase-matching wavelength position was experimentally demonstrated, with the generation of signal and idler photons spaced by more than 800 nm for the smallest waveguide width.

The use of multimode silicon waveguides for intermodal FWM experiments has also been reported by other research groups for telecom applications, for example for wavelength conversion from the C- to the L-band (1,565–1,625 nm) [64] and from the C- to the O-band (1,260–1,360 nm) [65], [66]. For example, Ronniger et al. reported all-optical wavelength conversion in a p-i-n diode-assisted multimode silicon-on-insulator (SOI) waveguide capable of converting the entire C-band into the O-band [65]. The implementation of all-optical wavelength converters in these wavelength bands can act as a bridge between long-haul C-band networks and shorter-range O-band networks, allowing to overcome common approaches based on electronics which typically face challenges related to unfavorable scalability in terms of power consumption and system complexity. The multimode waveguide was designed as a rib waveguide equipped with a p-i-n diode, which was used to efficiently remove free carriers generated by two-photon absorption (TPA) and, subsequently, free-carrier absorption (FCA) [67], [68]. In the integrated device demonstrated by the authors, the use of grating couplers (GCs) optimized for the C- and O-bands and input/output mode add-drop multiplexers (MADMs) allowed for coupling an O-band pump in the fundamental horizontal TE mode and a C-band pump and signal in the first-order horizontal TE mode of the multimode waveguide, respectively, with the converted idler in the O-band being then generated in the fundamental horizontal TE mode. C-to-O band wavelength conversion experiments of 32 GBd single-polarization (SP) QPSK signals at different wavelengths (1,542, 1,548 and 1,562 nm) were performed and compared with a back-to-back (B2B) measurement at 1,548 nm. An optical signal-to-noise ratio (OSNR) penalty lower than 0.4 dB at the hard-decision forward error correction (HDFEC) threshold from the C-to-O band converted data signals compared to the B2B case was measured. In addition, transmission over 100 km of standard single-mode fiber (SSMF) was performed for the 1,548 nm data signal and the bit error rate (BER) was measured for different launch powers of the converted signals. A relatively low launch power of only −5.5 dBm was sufficient to maintain performance below the HDFEC threshold. The experimental results showed that no noticeable distortion was present after the integrated wavelength converter.

BS FWM is another well-studied nonlinear process that enables the generation of red and blue shifted copies (*I*_*BS*,*r*_ and *I*_*BS*,*b*_ idlers, respectively) of the input signal through a scattering process induced by an intensity grating resulting from the interference between two strong pumps. This is, in principle, a noiseless process and, for this reason, has attracted considerable attention both for classical and quantum applications [69]. One limitation of this process in standard single-mode waveguides is that, for relatively small frequency shifts, BS FWM becomes bi-directional in frequency, and the input signal can be either blue- or red-shifted with similar probability and multiple scattering orders can occur, which may also result in power depletion and undesired interference in the case when multiple channels are present. Frequency uni-directional BS FWM between telecom wavelengths was first demonstrated in silicon waveguides by Bell et al. [70] using two pump pulses polarized on orthogonal axes and exploiting the waveguide birefringence. Uni-directional BS FWM in an intermodal configuration was demonstrated by Lacava et al. first in a silicon waveguide [62] and, in a later work, in a silicon-rich silicon nitride (SRSN) waveguide [71] using a dual-pump CW scheme. In both works, the two pumps were launched into the fundamental horizontal TE mode (TE_00_), while the signal and the generated idlers were in the first-order horizontal TE mode (TE_10_). Experiments were carried out in the C- and L-bands and spatial mode handling was performed off-chip using free space components. The use of SRSN instead of silicon provided an additional degree of freedom in the multimode waveguide design in addition to the waveguide cross-section thanks to the possibility of changing the material refractive index by varying the deposition conditions [72]. This enabled broadband wavelength conversion between the pump waves (separated by more than 30 nm) and a signal located in a different wavelength band, 50 nm away.

**Figure 2:**
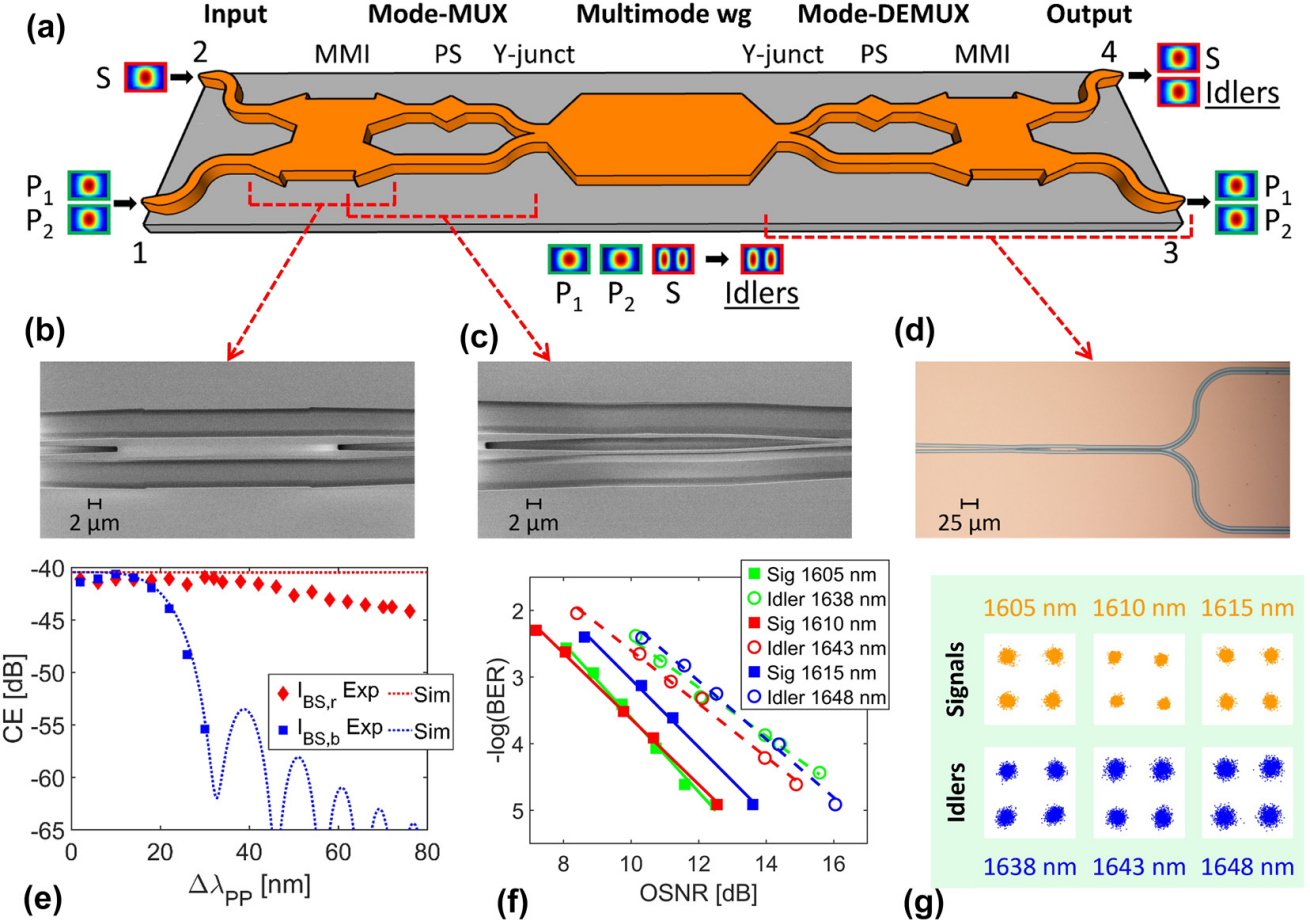
BS intermodal FWM experiments performed at telecommunication wavelengths. (a) Schematic layout of the fully-integrated BS intermodal FWM-based wavelength converter demonstrated in ref. [61]; (b) scanning electron microscope (SEM) images of MMI, (c) PS and Y-junction sections of the mode-MUX and (d) optical microscope image of the mode-DEMUX and output section; (e) experimentally measured and numerically simulated conversion efficiency (CE) values as a function of the pump-to-pump detuning Δ*λ*_*PP*_ for *I*_*BS*,*r*_ and *I*_*BS*,*b*_ with the wavelengths of *P*_1_ and *S* set at 1,540 and 1,600 nm, respectively. (f) BER curves as a function of the OSNR for the B2B L-band signals and converted U-band idlers measured using the fully-integrated BS intermodal FWM-based device reported in ref. [73] and (g) measured QPSK constellations for the signal and idler wavelengths before and after the wavelength converter, respectively. (a)–(e) Panels are adapted with permission from ref. [61] ©2024 Chinese Laser Press.

The same BS intermodal FWM (with two pumps in the TE_00_ mode, and the signal and idlers in the TE_10_) was then demonstrated by Vitali et al. [61] in a fully-integrated SRSN device integrating mode converter, multiplexing and de-multiplexing functionalities on-chip. The device layout is shown in Figure 2(a). The two pumps (*P*_1_ and *P*_2_) and the signal (*S*) were coupled simultaneously on ports 1 and 2 of the device, respectively, using an array of two polarization-maintaining lensed fibers. The mode converter and multiplexer (Mode-MUX), consisting of an MMI coupler, a 90° phase shifter (PS) and a Y-junction (Y-junct), ensured that the two pumps were maintained in the TE_00_ mode while converting the signal in the TE_10_ mode of the nonlinear multimode waveguide (multimode wg, 6.1 μm width × 310 nm thickness). The generated idlers in the TE_10_ mode were then converted in the TE_00_ mode and coupled to the output on port 4, together with the original signal, thanks to the mode converter and de-multiplexer (mode-DEMUX). The chip facets were prepared by high-precision mechanical dicing [74] and the coupling losses using inverted-taper-based edge couplers were measured to be 1.4 dB. Figure 2(b)–(d) shows images of the fabricated MMI, a detail of the mode-MUX and the full mode-DEMUX together with the output section, respectively. The nonlinear multimode waveguide was designed to satisfy the phase-matching condition between *P*_1_ at 1,540 nm and the signal at 1,600 nm, with the capability to achieve broad wavelength conversion for the *I*_*BS*,*r*_ idler by tuning the position of *P*_2_. Experiments were carried out using this wavelength configuration and Figure 2(e) shows the measured and numerically simulated conversion efficiency values as a function of the pump-to-pump detuning (Δ*λ*_*PP*_ = *P*_2_ − *P*_1_). A reduction in the conversion efficiency of the *I*_*BS*,*r*_ idler by 3 dB was observed for a pump-to-pump detuning as wide as 72 nm, giving rise to the generation of idler wavelengths across the range of 1,602–1,678 nm, covering substantial parts of the L-band and the whole of the U-band (1,625–1,675 nm). This represents the widest bandwidth ever reported in an intermodal FWM-based wavelength converter. As expected from numerical simulations, a much narrower bandwidth was measured for *I*_*BS*,*b*_ which was not efficiently phase-matched, confirming that the device offered uni-directional performance in frequency.

A device with similar features (designed to exhibit perfect phase-matching between *P*_1_ at 1,540 nm and the signal at 1,610 nm) was employed to perform L- to U-band wavelength conversion of 50-Gbps QPSK optical signals [73]. The modulated L-band signals in the wavelength range 1,605–1,615 nm were converted to the U-band in the range 1,638–1,648 nm by setting the two pump wavelengths *P*_1_ and *P*_2_ at 1,540 and 1,570 nm, respectively. Figure 2(f) reports the BER curves as a function of OSNR for the B2B L-band signals and the converted U-band idlers. As can be seen, the converted U-band idlers were received with a sensitivity penalty lower than 2.5 dB at a BER value of 10^−3^ compared to the measured B2B signals. Figure 2(g) reports the QPSK constellation diagrams of the L-band signals and the U-band converted idlers before and after the wavelength converter, respectively. The constellations confirm that the processed optical signals did not suffer any significant phase distortions during the wavelength conversion process. This work reported L- to U-band wavelength conversion of complex phase-coded telecommunication signals for the first time, highlighting the potential of intermodal FWM for optical communication applications.

While the demonstrations already reported in the literature proved the flexibility in the design and the large bandwidth achievable using intermodal FWM nonlinearities for wavelength conversion, the use of these devices in real telecom systems is still hindered by the typically low achievable conversion efficiency. In order to tackle this limitation, multiple optimization strategies could be simultaneously pursued. Material optimization to decrease linear and nonlinear losses together with the use of smaller nonlinear multimode waveguide cross-sections, for example using suspended waveguides with high-index contrast, may enhance the efficiency of the nonlinear process. Moreover, it should be noted that demonstrations reported in the literature on wavelength conversion via intermodal nonlinear processes have predominantly utilized relatively short, straight waveguides – typically only a few centimeters long. The reason can be found in the relatively large multimode waveguide cross-section of the demonstrated devices, which poses challenges in the realization of waveguide bends with low bending losses for all the considered modes and low mode cross-talk on a broad wavelength range. Overall, this limits the achievable conversion efficiency when using CW optical pumps at reasonable power levels. On the other hand, parametric gain in the CW regime was demonstrated in ultralow-loss, dispersion-engineered, meter-long silicon nitride (SiN) waveguides exploiting intramodal FWM processes [75], [76]. To achieve similar performance to their intramodal counterpart and enable practical implementations in real-world telecommunication systems, research should focus on developing meter-long multimode waveguides through optimized bend designs and waveguide geometries. This remains a key area for improvement in the next years, especially considering that intermodal processes inherently suffer from lower mode overlap compared to their intramodal counterparts, as previously discussed, which represents a limitation that may be challenging to overcome. In recent years, several works have been published on the optimization of multimode waveguide bends based on different approaches, such as shape-optimization with transformation optics [77], ultra-sharp bends with subwavelength gratings [30], designs based on B-spline curves [78] and on double free-form curves [79]. Even though much work still needs to be carried out, particularly in achieving broadband and fabrication-tolerant designs, thanks to these developments, it may become possible in the coming years to realize long, highly efficient nonlinear multimode waveguides suitable for integration into telecommunication systems. This would mark a significant step toward the practical deployment of intermodal nonlinear processes in real-world optical networks, with the possibility to efficiently convert signals between largely spaced telecom bands, which would otherwise be difficult to achieve using single-mode nonlinear devices. The realization of high-performance, meter-long multimode waveguides could also open the door to erbium-doped and, more generally, rare-earth-doped waveguide amplifiers capable of operating simultaneously on different spatial modes, thereby surpassing the performance of previously demonstrated single-mode devices [80]. In addition to optimizing the linear components, this would also require careful control of nonlinear dynamics to prevent intermodal cross-talk between the amplified channels propagating in different spatial modes. In addition, the design of advanced building blocks for mode conversion, (de)-multiplexing and, generally, mode manipulation functionalities together with the integration of laser sources on the same wavelength converter chip will enable a more efficient optical power coupling in the proper spatial modes and, hence, increase the overall efficiency of the system.

### 2.2 Generation of photon pair sources for quantum applications

Quantum information can be enabled by exploiting the different degrees of freedom of photons [81]. In the field of quantum computing, it is crucial to differentiate between physical and logical qubits. Physical qubits represent the two-level quantum systems that are processed within the quantum hardware. In contrast, logical qubits consist of clusters of redundant physical qubits that store information. On photonic platforms, the number of physical qubits is closely related to the number of photons [82]. The quality of these photons is quantified intrinsically by the purity of their states and extrinsically by their indistinguishability. Ensuring reliable physical qubits necessitates the development of high-quality photon sources [83].

There are currently two main types of integrated photon sources: deterministic and probabilistic [84]. Deterministic sources, when integrated into a silicon photonic-integrated circuit (SiPIC), remain challenging due to high coupling losses and complex fabrication processes [85]. In addition, they typically work at cryogenic temperatures and in the visible spectral range. On the other hand, there have been numerous reports of probabilistic photon pair sources successfully integrated into SiPICs over the past decade [86]. Probabilistic sources are based on spontaneous parametric processes enabled by nonlinear optics: spontaneous parametric down-conversion (SPDC) uses second-order nonlinearities while spontaneous FWM uses third-order nonlinearities. SPDC is more effective than spontaneous FWM. In integrated optics, a dominant material platform is silicon photonics, where only third-order nonlinear processes are possible [87]. In silicon photonics, correlated photon pairs can be generated by intramodal spontaneous FWM either using long waveguides arranged in a spiral configuration to decrease their footprint or by compact microring resonators. Spiral waveguide results have better performance in indistinguishability than microring resonators [88]. This can be quantified by the joint spectral amplitude (JSA) overlap, which can be as high as 98 % in spiral waveguides, as opposed to 89 % in microring resonators. The purity of the generated photon pairs plays a significant role when the generated photons have to interfere with photons produced by other independent photon sources. However, in a generic quantum application, it is desirable to have large values for both purity and indistinguishability because of the requirement of high visibility for dependent as well as independent sources. On one hand, spiral waveguides show a high visibility and JSA overlap but a low purity and low brightness, while, on the other hand, a low visibility and JSA overlap but a high purity is observed in microring-based photon pair sources. This prevents qualifying one source as better than the other *a priori*; instead, the selection should be based on which source delivers the necessary performance to accomplish a specific task [88]. Intermodal spontaneous FWM enables the implementation of both high purity and high indistinguishability correlated photon sources in spiral waveguides [86]. On the other hand, in a wide class of applications, single photons are required. On-chip single photon generation can be achieved through spontaneous FWM and heralding [86]. Here, correlated photon pairs are exploited, with one photon serving as a herald for the presence of the other, which functions as the single photon in the quantum device. This method is known as a heralded single photon (HSP) source, and offers ease of integration, flexibility, and the ability to monitor the photon generation rate. Although the generation process is probabilistic, it can be made nearly deterministic through multiplexing [89]. Therefore, HSP sources represent a practical approach to on-chip quantum photonics. To produce high-purity photon states, the spectrum generated by FWM is typically post-filtered to ensure a factorable joint spectral intensity (JSI), which enhances purity, albeit at the expense of lower brightness and heralding efficiency [86]. Additionally, the required narrow-band filtering limits the scalability of HSP solutions in terms of both integration and cost. To eliminate the need for post-filtering, one can leverage discrete band phase-matching provided by intermodal FWM [58]. This approach naturally filters the FWM spectrum, resulting in high-purity, high-brightness, and high-heralding-efficiency single photon states. Eliminating post-filtering also simplifies integration and scalability. Furthermore, intermodal FWM offers significant flexibility in terms of bandwidth and phase-matched band positioning, enabling effective control over the JSA. It allows for large detunings between the signal and idler frequencies, which simplifies the filtering of pump photons and the use of optimized single-photon detectors.

A first demonstration of the use of multimode waveguides as correlated photon pair sources used SPDC. A periodically poled multimode waveguide in potassium titanyl phosphate (KTP) was used to demonstrate polarization entanglement of different degenerate modal orders at 800 nm by pumping at 400 nm [90]. Violation of the Bell inequality with a Clauser–Horne–Shimony–Holt (CHSH) parameter up to 2.319 ± 0.006 was achieved without the use of spatial filters. The first report of intermodal spontaneous FWM in silicon waveguides demonstrated the generation of photon pairs in multimode waveguides with different mode combinations [58], thus paving the way for quantum-related demonstrations using third-order nonlinearities. In ref. [63], an integrated silicon photonic chip was demonstrated which allowed efficient excitation of spontaneous FWM and simplified separation of the generated photon pairs from the pump photons using ADCs. Correlated photon pair generation was demonstrated on-chip by using up to the third-order waveguide mode. This design demonstrated the advantages of intermodal spontaneous FWM for entangled photon generation, while, however, suffering from limitations relating to brightness, group velocity, losses and efficient scalability.

**Figure 3:**
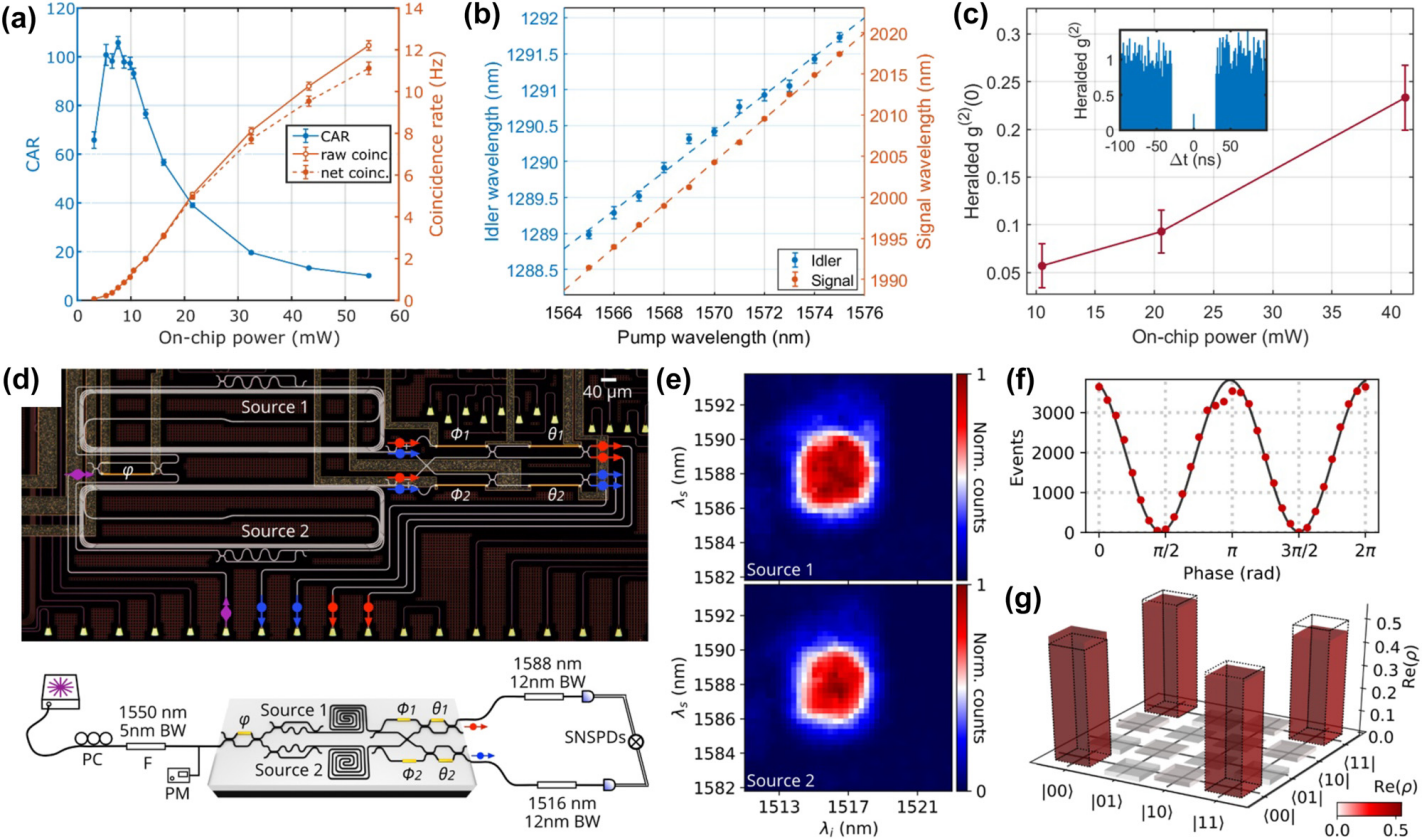
Intermodal FWM experiments for quantum applications. (a) Characteristics of an intermodal spontaneous FWM-based correlated photon source: coincidence to accidental ratio (CAR, blu symbols and line) and coincidence rates (red symbols and lines) as a function of the on-chip power from ref. [92]; (b) tuning of the idler and signal wavelengths as a function of the pump wavelength from ref. [92]; (c) heralded second-order coherence function ( $g_{H}^{\left(2\right)}$ (0)) as a function of the on-chip pump power. The inset shows the measured $g_{H}^{\left(2\right)}$ as a function of the time delay (Δ*t*) between the two single photon avalanche diodes (SPADs) clicks at an on-chip power of 10.5 mW. The adjacent bins to the zero-delayed one were discarded due to the SPAD-emitted photons [92]. (d) Optical microscope image (top panel) of the SiPIC with two identical delayed-pump intermodal spontaneous FWM photon sources demonstrated in ref. [93] with the corresponding schematic and experimental setup (bottom panel) used to measure the source indistinguishability (*ϕ*_1_, *ϕ*_2_: phase shifters; *φ*, *θ*_1_, *θ*_2_: internal phases of MZIs; PC: polarization controller; F: filter; BW: bandwidth; PM: optical power monitor; SNSPDs: superconducting-nanowire single photon detectors); (e) individual JSIs experimentally measured with separate pumping of source 1 (top panel) and source 2 (bottom panel) [93]; (f) measured reversed-HOM fringe from the two sources. The fringe visibility, which corresponds to the source indistinguishability, is equal to 0.987 ± 0.002 [93]; (g) density matrix of the two-qubit entangled state measured when the two sources were coherently pumped and reconstructed via quantum state tomography. The source indistinguishability estimated from the quantum state tomography is 0.982 ± 0.006 [93]. (a)–(c) Panels are reproduced from ref. [92] under a CC BY 4.0 license. (d)–(g) Panels are adapted from ref. [93] under a CC BY 4.0 license.

An entangled photon source of photons widely separated in wavelength was demonstrated in ref. [91] by carrying out coincidence measurements of the generated photon pairs. This report also included a thorough discussion on the influence of the various linear and nonlinear losses on the correlated photon generation. In particular, the signal to noise ratio of the source was quantified by considering the coincidence to accidental ratio (CAR). True coincidences come from the simultaneous detection of a signal photon and an idler photon belonging to the same photon pair. Coincidences between photons belonging to different pairs or coincidences with noise photons or dark counts contribute to the accidentals. A similar SiPIC as the one shown in Figure 1(e) was used in this demonstration: a 1.5 cm long, 190 nm height and 1.95 μm wide multimode waveguide was pumped with 1,550 nm pump photons to generate photon pairs at 1,260 nm and 2,015 nm. The photon source showed a CAR of 40 with a net coincidence rate of 26 Hz at an on-chip peak power of 1.2 W. Improvements in the SiPIC design and fabrication process obtained by Sanna et al. allowed these values to be improved to a maximum CAR of 100 with CW 1,550 nm pumping (see Figure 3(a)) [92]. These values also provide evidence of a high generation rate of 12 Hz at an on-chip power of 55 mW with a large pair wavelength tunability (see Figure 3(a)). Interestingly, due to the refractive index dispersion, it was observed that small variations in the pump wavelength (about 12 nm) gave rise to large variations in the MIR region (about 30 nm for the signal wavelength, see Figure 3(b)), improving what can be achieved by intramodal FWM. Here, also the heralded second-order coherence function, $g_{H}^{\left(2\right)}$ (0), was measured [86]. This parameter quantifies how close the source is to single photon behaviour and is measured by a Hanbury–Brown and Twiss (HBT) interference measurement on one photon of the pair while detecting the other photon. Figure 3(c) shows a minimum value of 0.06 at 10.5 mW on-chip power, which confirms the anti-bunching regime of the generated photon in the MIR and, hence, the single photon regime of the source. In ref. [94], the different spatial modes of a multimode waveguide were used as a degree of freedom to label different quantum states. Here, the idea was to entangle photons that propagated in different modes to get a transverse-mode entanglement. A significant advantage of this approach is the possibility to exploit also other degrees of freedom of the photon states (e.g., the temporal, spatial, polarization and frequency degrees of freedom) to generate hyper-entangled states which have a variety of applications [95]. They demonstrated a dual-spatial-mode pumping scheme to generate transverse-mode entangled photon pairs on-chip [94]. Entanglement was verified both by quantum state tomography as well as by fidelity measurements which yielded a Bell-state fidelity of up to 0.96 ± 0.01. The proposed SiPIC was similar to the one shown in Figure 1(e). A 3 mm long multimode waveguide with a cross-section of 760 × 220 nm^2^ was used so that the TE_0_ and TE_1_ modes could be supported. The generation of pure transverse states (both photons in a single mode) or mixed states (photons in different modes) is controlled by tuning the energy ratio and phase difference of the pump photons. In this way, maximally entangled Bell states $\left|\Phi \right\rangle =1/\sqrt{2}\left(\left|{\text{TE}}_{1}{\text{TE}}_{1}\right\rangle +\left|{\text{TE}}_{0}{\text{TE}}_{0}\right\rangle \right)$ with a fidelity approaching 1 were obtained.

A further improvement was reported by Paesani et al. [93]. Intermodal spontaneous FWM was used for the generation of entanglement and a sophisticated tailoring of the intermodal phase-matching to produce discrete phase-matching bands far from the pump was employed to achieve uncorrelated photons. This improved the single photon spectral purity without requiring the use of filters. Energy conservation in spontaneous FWM induces a strong anticorrelation between the frequencies of the generated photons which can be mitigated by the discrete generation bands of intermodal spontaneous FWM. By tailoring the waveguide cross-section, the modal dispersion can be accurately engineered to design the phase-matching band with a bandwidth similar to that of the pump (related to energy conservation). This suppresses the frequency anticorrelations imposed by energy conservation and enhances the spectral purity of the emitted photons. Moreover, to obtain a near-unit spectral purity, the residual correlations in the JSA were suppressed by inserting a delay on the fundamental mode component of the pump (with higher group velocity than the other pump mode) before injecting it into the source. The temporal walk-off between the two modes was thus gradually decreased and then increased as the fastest photons overtook the slowest ones. This resulted in an adiabatic reversing in the momentum of nonlinear interactions in the waveguide, which suppressed spurious spectral correlations. This technique is called the delayed-pump technique. In ref. [93], an 11 mm-long spiral waveguide in a 200 × 900 μm^2^ footprint was used. The waveguide had a cross-section of 2 × 0.22 μm^2^ and supported the TM_0_ and TM_1_ modes. The delayed-pump excitation scheme was implemented as shown in the layout reported in Figure 3(d), which comprised two identical delayed-pump intermodal spontaneous FWM photon sources (“Source 1” and “Source 2”). The pump, initially in the TM_0_ mode, was split by a balanced beam-splitter and a serpentine was added to one arm to induce a delay on the TM_0_ mode. At the same time on the other arm, an ADC converted the TM_0_ into a TM_1_ mode while coupling it to the multimode waveguide. Therefore, a delayed TM_0_ photon and a TM_1_ photon (which was not delayed) were injected in the multimode waveguide as pump photons to generate the photon pair. Once generated, the signal photon was separated from the idler via a second TM_1_ to TM_0_ mode converter. Optimum results were achieved when the temporal delay caused by the serpentine on the fundamental pump mode had a value *τ* = 1.46 ps. An 8 MHz photon pair generation rate, an on-chip heralded single photon $g_{H}^{\left(2\right)}\left(0\right)$ of 0.053 ± 0.001, a spectral purity of 0.9904 ± 0.0006, a mutual indistinguishability of 0.987 ± 0.002, and an intrinsic heralding efficiency exceeding 90 % were achieved [93]. The capability of the sources to generate pure photons with no requirement for filtering enables high heralding efficiency and high purity to be achieved at the same time. The experimental setup used to enable quantum interference between different sources and assess the sources’ indistinguishability is also shown in Figure 3(d). In this SiPIC, two sources were coherently pumped by splitting the pump through the use of an on-chip tunable Mach-Zehnder interferometer (MZI). The idler and signal modes from the different sources were combined and interfered on-chip using additional integrated phase shifters and MZIs. Three different measurement methods were used [86]. First, the JSI of each source was individually evaluated (see Figure 3(e)). The overlap of the JSIs from each source indicated a mutual indistinguishability of 98.5 ± 0.1 %. Then, a reversed Hong–Ou–Mandel (HOM) interference between the two sources was performed, with the results shown in Figure 3(f). The visibility of the reversed HOM fringe was measured to be equal to 98.7 ± 0.2 %, directly reflecting the source indistinguishability. Finally, the entanglement generated when the two sources were coherently pumped was quantified through quantum state tomography. In this experiment, the density matrix of the entangled states was measured (see Figure 3(g)), which showed a fidelity of 98.9 ± 0.3 % with the ideal maximally entangled state $|\Phi_{+}〉=\left(|00〉+|11〉\right)/\sqrt{2}$ , yielding an indistinguishability value of 98.2 ± 0.6 % [93].

The observation of high indistinguishability between different on-chip sources paves the way to scaling up the system. A single pump laser can be coherently split on-chip to synchronously and coherently feed many sources together. Estimations show that one could simultaneously operate more than 250 sources in parallel, which can enable experiments with tens of photons. Multi-photon experiments at this scale are expected to enter computationally interesting regimes where quantum machines can compete and surpass classical supercomputers [96], [97].

To further improve indistinguishability in the presence of fabrication errors in the delayed-pump intermodal spontaneous FWM scheme, a tapered multimode waveguide design was suggested [98]. By controlling the relative delay of the pump photons, it was possible to reduce drastically the distinguishability from different sources due to fabrication imperfections. As an example, errors up to 4 nm in waveguide height and up to 100 nm in waveguide width were accounted for, while keeping an indistinguishability level ≥95 % [98]. The proposed design applied both to different sources on the same chip and in different chips. Finally, a theoretical work was reported, which merged the concept of transverse mode entanglement generation by the dual pump scheme with the delayed-pump approach [99]. It was demonstrated that pure Bell states in the transverse waveguide mode can be generated, where the photon pairs can be nearly perfectly uncorrelated in frequency.

**Figure 4:**
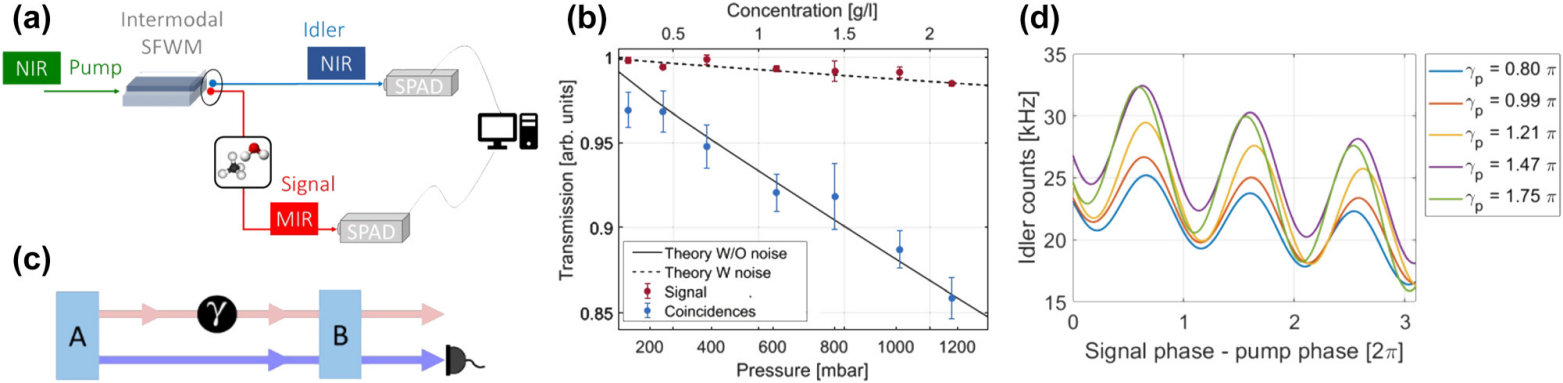
Ghost spectroscopy measurements and undetected photon quantum interference experiments based on intermodal FWM. (a) Experimental setup used in ref. [92] for the ghost spectroscopy measurements of the CO_2_ absorption (SFWM: spontaneous FWM) and (b) corresponding transmission as a function of the gas pressure and concentration in the gas chamber for the coincidence measurements (i.e., in the ghost spectroscopy settings, blue dots) and for the direct signal transmission at 2,003.3 nm (red dots). The lines represent the expected transmission intensity for direct measurement in the presence (dashed line) or absence (solid line) of the noise [92]. (c) Concept of the undetected photon quantum interference experiments presented in ref. [101]. (d) Measured idler photon counts as a function of the phase applied to the signal photons minus the phase applied to the pump beam (*γ*_*p*_) in the TE_1_ mode. The lines represent the fit of the experimental data and are plotted for different values of *γ*_*p*_ [101]. (a)–(b) Panels are reproduced from ref. [92] under a CC BY 4.0 license. (c)–(d) Panels are reproduced with permission from ref. [101] ©2023 SPIE.

An exemplary application of the advantages of intermodal spontaneous FWM is in ghost spectroscopy measurements [100]. Here the temporal and energy correlations of the generated photon pairs were used to perform gas absorption measurements in conditions of high environmental noise. The principle of this technique is shown in Figure 4(a). A SiPIC similar to the one shown in Figure 1(e) was used to generate photon pairs in the near-infrared (NIR) and MIR [92]. These were collected by two different fibers at the output of the SiPIC and, therefore, were spatially separated. The MIR photon was made to interact with a gas chamber while the NIR photon was spectrally analyzed. The energy and temporal correlation between the photons in a pair allowed to transfer the information from the MIR photon which interacted with the gas to the NIR photon via coincidence measurements. Absorption measurements on CO_2_ at 2 μm showed that a higher sensitivity in a noisy environment was obtained with the coincidence measurements with respect to direct MIR signal photon measurements (blue and red dots in Figure 4(b), respectively) [92].

Another example highlighting how useful the intermodal spontaneous FWM scheme can be for the generation of correlated photon pairs is based on the indistinguishability of two intermodal spontaneous FWM sources [102] and is discussed in the following. Figure 4(c) shows a possible configuration for undetected photon quantum interference experiments. Two intermodal spontaneous FWM entangled photon sources (“A” and “B”) were made collinear and coherently pumped in a single SiPIC, such that it was impossible to determine whether the first or the second source generated the photon pair by observing the output photons. The generated photon pairs must be indistinguishable in all degrees of freedom. In this way, quantum interference occurred between two events: generation of the pair in the first source A or generation of the pair in the second source B [102]. The visibility of the interference holds the information about the indistinguishability of the generation processes. Owing to the spatial separation between the generated photons in the intermodal spontaneous FWM scheme (see Figure 4(c)), it was possible to induce a perturbation on one of the two photons and detect it by looking at the other. In the experimental demonstration reported by Michelini et al. [101], the perturbation was induced by applying a phase shift *γ* on the signal photons (red path in Figure 4(c)) between the two collinear sources. Measurements of the quantum interference on the idler photons (blue path) while tuning the phase of the signal photons provided evidence of the undetected photon measurement, allowing the phase of the signal photons to be probed by measuring only the idler photons (see Figure 4(d)) [101].

Intermodal FWM offers significant advantages for generating entangled photon pairs by utilizing the spatial mode degree of freedom, enabling perfect phase matching at non-degenerate wavelengths and increased spectral tunability. This spatial control further allows for efficient on-chip separation and manipulation of photons using DCs and modal interferometers [103], [104]. Moreover, it yields exciting possibilities for squeezed state generation and tailored quantum noise reduction [105], for enhancing the robustness and security of quantum communication and quantum key distribution [106] and for implementing quantum gates for quantum computing [107]. Finally, it allows for improving the precision of quantum sensing and imaging [108], and advancing quantum metrology through control over quantum noise properties [109].

### 2.3 Frequency generation towards the mid-infrared

The MIR spectral region, spanning approximately from 2 to 20 μm, is crucial for spectroscopic techniques used to detect and analyse various molecular species [110], [111]. Several relevant gases exhibit their fundamental absorption lines in this region, making MIR sources invaluable for spectroscopic applications in environmental sensing [112], medical diagnostics [113], and industrial process monitoring [114]. However, the generation of light across the MIR spectrum is challenging, largely due to the scarcity of efficient gain media that can operate in this range. Traditional MIR light sources, such as optical parametric oscillators (OPOs) based on bulk crystals, have been successful but suffer from several drawbacks, including their bulky size, high cost, and complexity of their setup. These limitations have driven the search for more compact, cost-effective, and efficient alternatives. One promising approach involves QCLs, which have shown great potential due to their ability to provide narrow, tunable linewidths [115]. However, QCLs are generally limited in output power and most commercially available QCLs are in the 4–11 μm wavelength range, which constrains their use in applications requiring MIR light across a broader range.

An alternative and increasingly popular approach is the use of nonlinear frequency generation in integrated waveguides. This method leverages the high power and tunability of NIR sources to generate MIR light through nonlinear optical processes. The attraction of using integrated waveguides includes their compactness, scalability, and potential for integration with other on-chip components. Initially, research in this area focused on single-mode phase-matching in second-order nonlinear processes, such as DFG, which underpins the operation of optical parametric amplifiers (OPAs) and OPOs. However, the inherent limitations of single-mode phase-matching for MIR generation soon became apparent, leading researchers to explore intermodal phase-matching techniques. This section provides an overview of the development and application of intermodal phase-matching for on-chip MIR generation, mainly focusing on second-order nonlinearities. These processes rely on materials with a non-centrosymmetric crystal structure, which allows for interactions like SHG, SFG and DFG. III–V semiconductors, such as gallium arsenide (GaAs) and aluminum gallium arsenide (AlGaAs), have been at the forefront of research into second-order nonlinear processes for MIR generation. These materials are particularly attractive due to their high nonlinear coefficients and the ability to engineer their bandgap and dispersion properties [116], [117]. However, the lack of natural birefringence in materials with zinc blende structures poses significant challenges for satisfying the phase-matching condition. Historically, phase-matching in III–V semiconductors was initially achieved using Bragg reflection waveguides (BRWs). Early work in the 1970s demonstrated SHG in a GaAs waveguide by phase-matching the TE and TM modes, enabling the generation of light at around 5 μm from a CW CO_2_ laser source tunable around 10 μm [118]. These early demonstrations laid the groundwork for subsequent developments in the field. In the 1990s, Fiore et al. demonstrated MIR generation exploiting intermodal DFG in III–V semiconductors, achieving the generation of light at 5.3 μm using two CW pump lasers (a Nd:YAG laser with a wavelength of 1.32 μm and a tunable Titanium:Sapphire (Ti:Sa) laser set at a wavelength of 1.058 μm) [119]. Specifically, they designed the device to satisfy the phase-matching condition over such a broad wavelength range by exploiting a built-in artificial birefringence in a composite GaAs-based multilayer material, where the isotropy of bulk GaAs was broken by inserting several thin oxidized aluminum arsenide (AlAs) layers in GaAs. Birefringent phase-matching was achieved in the composite material by tuning the properties of the supported TE and TM modes. Moreover, the tunability of the generated MIR radiation was demonstrated by varying the material temperature, showing the capability to cover the wavelength range from 5.2 to 5.6 μm with a temperature scan from 0 to 150 °C. Although these planar waveguide structures were effective in satisfying the phase-matching condition, they suffered from limited mode overlap, which resulted in low conversion efficiencies.

The introduction of integrated ridge waveguides marked a significant advancement in the field, improving mode overlap and allowing for more efficient DFG. Abolghasem et al. demonstrated non-degenerate DFG in the MIR region in a single-sided BRW, where, unlike standard BRWs, the Bragg mode was confined by a Bragg mirror in the lower cladding while it was evanescent in the upper cladding [120]. The Bragg mirror, a multi-layered core and the upper cladding were defined by different AlGaAs layer compositions and ridge waveguides were fabricated. In this work, the pump mode was a Bragg mode, while the signal and the generated idler modes were guided by total internal reflection. CW generation of a TE-polarized idler in the range of 2.36–2.53 μm was reported using a TE-polarized pump tuned between 938 and 952 nm and a TM-polarized signal tuned between 1,490 and 1,590 nm.

**Figure 5:**
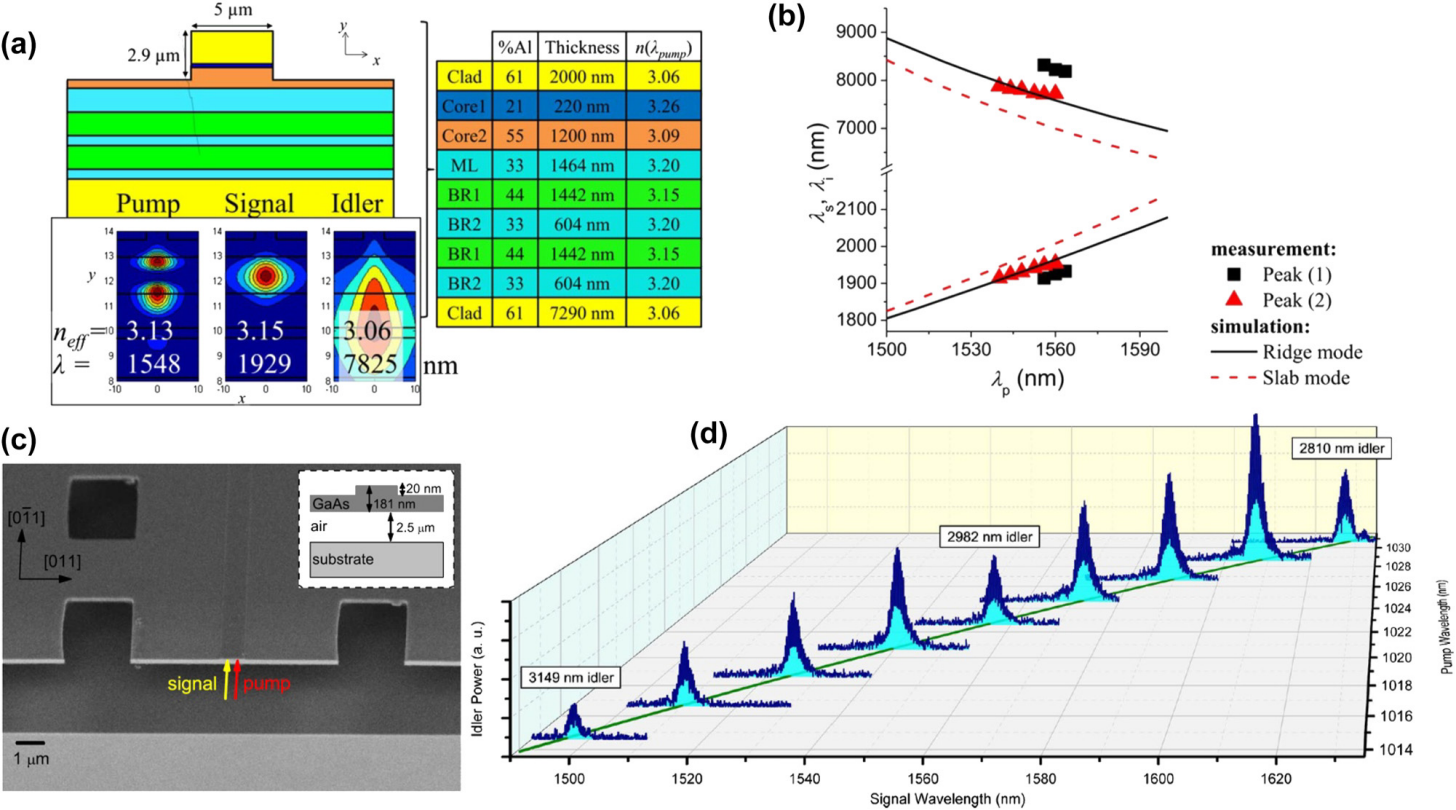
Intermodal DFG experiments for MIR generation. (a) Waveguide structure together with the layer details and the interacting pump, signal and idler modes used for the DFG experiments reported in ref. [121] and (b) experimentally measured phase-matched signal-idler wavelength pairs as a function of the pump wavelength together with the numerically simulated phase-matched curves considering the interaction of three ridge (in black) and three slab (in red) modes. (c) SEM image of a suspended GaAs rib waveguide with a width of 1 μm used in the DFG experiments reported in ref. [122], with the inset showing a schematic of the suspended waveguide, and (d) experimentally measured idler power for a 2 μm-wide suspended GaAs waveguide as a function of the signal and pump wavelengths. The solid green line shows the numerically calculated phase-matched signal wavelength as a function of the pump wavelength. (a)–(b) Panels are reproduced with permission from ref. [121] ©2013 Optica Publishing Group. (c)–(d) Panels are reproduced with permission from ref. [122] ©2014 Optica Publishing Group.

MIR generation in the 7.5–8.5 μm range was also reported in a multilayer AlGaAs BRW through DFG between a pump and a signal tunable at around 1,550 and 1,950 nm, respectively [121]. In this work, the AlGaAs multilayer waveguide was carefully designed to support a BRW mode at 1,550 nm to serve as the TE-polarized pump and to satisfy modal phase-matching with a TM-polarized signal and a TE-polarized idler at the desired wavelengths. The waveguide layout together with the layer details and the pump, signal and idler mode profiles are shown in Figure 5(a). The device consisted of a lower and upper cladding (clad), a dual-layer core, a matching layer (ML) and a Bragg reflector (BR) stack with two periods. The 7.29 μm thick low index (61 % Aluminum (Al) content) bottom cladding layer was employed to confine the large long-wavelength idler mode. As can be seen, the spatial mode overlap between the different interacting waves was limited by the multi-lobed BRW mode of the pump and the mode profile distributions at the significantly distant wavelengths. Figure 5(b) reports the measured signal and idler wavelength pairs, *λ*_*s*_ and *λ*_*i*_, respectively, as a function of the pump wavelength, *λ*_*p*_, resulting from the DFG experiments and estimated from two different power peaks in the recorded spectra, which resulted from the interaction of three ridge (in black) and three slab (in red) waveguide modes. The ridge mode interaction was responsible for the stronger power peaks and, according to numerical simulations, would allow an idler to be generated in a broad wavelength range from 6.9 to 8.9 μm by tuning the pump wavelength from 1,500 to 1,600 nm and the signal wavelength from 1,800 to 2,100 nm.

Despite the impressive signal-idler wavelength distance reported in these works, efficiencies remained relatively low, typically below 1 %W^−1^cm^−2^, mainly due to the multi-layer waveguide arrangements in these BRWs, leading to a large mode area and then ultimately hindering the efficiency of the nonlinear conversion [120], [121]. Recent advancements in waveguide technology have focused on suspended and on-insulator platforms, which offer significantly enhanced mode confinement. For instance, suspended GaAs rib waveguides exhibiting birefringent phase-matching between the TE_00_ and TM_00_ modes have been proposed. Such a demonstration achieved DFG with a CW pump tunable between 1,018 and 1,032 nm and a CW signal tunable between 1,490 and 1,620 nm to generate idlers with a conversion efficiency as high as 2,800 %W^−1^cm^−2^ in the 2,800–3,150 nm spectral band [122]. A SEM image of a fabricated suspended GaAs rib waveguide is shown in Figure 5(c) together with the material stack reported in the figure inset. The GaAs waveguides were patterned over a 2.5 μm thick sacrificial Al_0.65_Ga_0.35_As layer, which was removed with a selective HF:H_2_O wet etch, which acted on the sacrificial layer through previously defined etch-holes spaced 3–8 μm from the edge of the waveguide. Figure 5(d) reports the results of the DFG experiments showing the idler power for different fixed pump wavelengths and as a function of the signal wavelength. As can be seen, the tuning of the pump and signal by just 16 and 130 nm, respectively, allowed the generation of idler components approximately between 2,800 and 3,150 nm, resulting in an overall tuning of 350 nm in the MIR.

The use of suspended GaAs waveguides has several advantages compared to more commonly used waveguides typically embedded in silica. First, the relatively high index contrast along with the absence of an oxide surrounding material could potentially enable phase-matching in discrete spectral regions at wavelengths even beyond 10 μm thanks to the large transparency of GaAs [123]. Second, high conversion efficiencies can be achieved thanks to the relatively small effective areas of the interacting modes, which are mainly confined to the GaAs layer. However, these waveguides are still limited by TPA at high powers and fragility due to the absence of a cladding material. The use of a multimode AlGaAs-on-insulator waveguide for intermodal DFG-based MIR generation was theoretically proposed by Haines et al. [124]. They numerically demonstrated that by using a 1.7 cm-long multimode waveguide with a cross-section of 265 × 1,200 nm^2^ pumped by a beam in the first higher-order horizontal TM mode at a wavelength of 1,550 nm and seeded by a signal in the fundamental TE mode at a wavelength of ≈2,693 nm, it is possible to generate an idler component in the fundamental TE mode at ≈3,652 nm. Assuming propagation losses equal to 2 dB/cm, it was shown that it is possible to achieve a pump-to-idler conversion efficiency of up to 15 %. The use of DFG for MIR generation around 2.3 μm in an AlGaAs-on-insulator waveguide was also numerically demonstrated by Madsen et al. achieving birefringent phase-matching using the TM_00_ mode for the pump and the TE_00_ mode for the signal and idler [125]. The authors demonstrated the possibility of generating signals at a ≈ mW-scale power level in the wavelength range 2,231–2,574 nm in a 5 mm long waveguide by using tunable sources around 940 and 1,550 nm.

Ferroelectric materials, particularly lithium niobate (LiNbO_3_), have also played a crucial role in the development of nonlinear optical devices. LiNbO_3_ is well known for its large electro-optic and nonlinear optical coefficients, making it an ideal candidate for a wide range of applications, including MIR generation. The development of periodic poling technology in the late 1980s and early 1990s revolutionized the field by providing a reliable method for achieving quasi-phase-matching (QPM) in ferroelectric materials. Periodically poled lithium niobate (PPLN) waveguides became a standard platform for efficient SHG and DFG, enabling the generation of MIR light in compact, integrated devices [126], [127]. The introduction of thin-film LiNbO_3_ (TFLN) on insulator platforms has further enhanced the performance of these devices. Several demonstrations of periodically poled TFLN on sapphire waveguides have been reported in the literature for MIR generation using intramodal second-order nonlinear processes [12], [128], [129]. However, the selective domain engineering of the LiNbO_3_ crystal required to realize the periodic poling and, hence, achieve QPM, may be difficult to scale as a manufacturing process for integrated circuits due to its variability. Therefore, the use of intermodal phase-matching techniques in TFLN waveguides is considered as an alternative and more fabrication-friendly approach. Broadband and efficient SHG in TFLN waveguides was numerically demonstrated exploiting both modal phase matching between two fundamental modes [130] and also considering higher-order modes [131], [132]. Moreover, experimental demonstrations of poling-free SHG generation based on intermodal phase-matching between the fundamental mode and higher-order modes were reported considering standard TFLN waveguides [133], [134] and SiN-loaded LiNbO_3_ on insulator waveguides [135], without the need to either pole or etch the LiNbO_3_ layer in the latter case. Nevertheless, although there has been a great breakthrough in SHG based on birefringence and intermodal phase-matching techniques in LiNbO_3_ waveguides [130], [131], [132], [133], [134], [135], [136], no experimental demonstrations of intermodal DFG-based MIR generation have been reported so far, despite the great potential in this material platform. A dual-layer TFLN on sapphire waveguide with DFG phase-matching between a 980 nm TE_01_ mode pump and a fundamental TE_00_ mode signal tunable in the range 1,340–1,440 nm was proposed and only numerically demonstrated [137]. A possible 340 nm bandwidth centred at 3,320 nm for the generated idler in the fundamental TE_00_ mode was reported with a theoretical conversion efficiency of 434.6 %W^−1^cm^−2^ made possible by the two reversed poled TFLN waveguide layers.

Third-order nonlinear processes, primarily driven by the Kerr effect, represent a promising alternative for MIR generation in silicon and SiN photonics. These processes, including FWM, are attractive because they are present in materials with centrosymmetric crystal structures, which are more readily available and whose fabrication in waveguide structure can be compatible with standard photonic integration techniques. Similar to TFLN waveguides, most of the demonstrations reported in the literature so far for FWM-based MIR generation rely on the use of intramodal processes [138], [139]. Early studies on intermodal FWM for MIR generation demonstrated the potential of this technique, as discussed in the previous section [58], [63], where intermodal phase-matching in the wavelength range from 1.2 to almost 2.2 μm was experimentally demonstrated using optical pumps at telecom wavelengths. Current research in this field is at a more exploratory stage, with theoretical and simulation studies suggesting the feasibility of generating light deep into the MIR using Kerr-driven intermodal phase-matching and pumping in the near-infrared. In this regard, a numerical study by Franz et al. discusses two different design strategies to achieve wideband light generation using intermodal nonlinear Kerr processes [140]. The first one consists of achieving a mirror symmetry of the inverse group velocities of the interacting modes far from the pump, thus allowing to satisfy the phase-matching condition in the case of a very large signal-to-idler wavelength separation and over a wide spectral region. Applying this approach, the authors numerically demonstrated the possibility of generating MIR radiation at wavelengths even beyond 7 μm based on suspended silicon waveguides. A second strategy for achieving wideband light generation involves concatenating multiple spectral regions where distinct mode pairs are phase-matched, thereby creating a wide, continuous band with high power spectral density.

As discussed in this section, intermodal nonlinear processes in integrated waveguides may enable flexible and broadband MIR light generation by leveraging the additional degree of freedom provided by multimodality to satisfy the phase-matching condition. In this context, second-order nonlinear processes in materials such as III–V semiconductors have shown great promise, particularly with the development of advanced phase-matching techniques and novel waveguide designs. At the same time, third-order nonlinearities, especially in silicon-based platforms, are emerging as a powerful tool for broadband and tunable MIR generation. Despite the advances reviewed above, significant challenges remain. The performance of these systems is often limited by issues such as TPA, FCA, and low fabrication tolerances. Future research should focus on overcoming these limitations through improved material systems, innovative waveguide designs, and the development of new phase-matching techniques. Additionally, the integration of these technologies into fully functional photonic circuits, including sources, modulators, and detectors, will be crucial for realizing the full potential of on-chip MIR generation.

It should be acknowledged that progress in QCLs and interband cascade lasers (ICLs) (the direct competitors for MIR frequency generation) is impressive, especially in terms of achieving a narrow linewidth, high power and high wall-plug efficiency, which now largely exceeds 20 %, approaching the theoretically predicted maximum [141], [142], [143]. Nevertheless, the realization of narrow-linewidth sources by combining well-established NIR lasers with nonlinear multimode waveguides may offer a viable and lower-complexity alternative to QCLs and ICLs, with improved performance in terms of tunability, albeit with a lower overall efficiency. As previously discussed, careful engineering of the phase matching condition between multiple waveguide modes could potentially enable the generation of narrow linewidth, widely-tunable and high-power light across the MIR. However, as this review has shown, we are still far from the realization of high-power sources using this approach. Ongoing research aims at boosting output powers across the various schemes discussed in this review through emerging technologies focused on reducing losses, improving chip power handling, and enhancing coupling efficiency between sources and waveguides, among other advancements. Despite the competition between the two technologies, with QCLs and ICLs currently dominating, possible combinations can still be envisioned. In this context, the challenges of QCLs and ICLs in power scaling could be addressed using parametric amplification through various intermodal phase-matching schemes in meter-long multimode waveguides, as discussed in Section 2.1, enabling the efficient transfer of NIR power sources into the MIR.

## 3 All-optical signal processing

Optical interconnects and communications have fed the insatiable demand for an ever increasing network capacity, while offering low power consumption, high bandwidth, and low latency. The introduction of optical logic gates and optical switches in such systems promises to add critical functionalities to the optical layer. Optical logic gates facilitate complex data processing directly in the optical domain, enhancing network performance and efficiency by eliminating the need for optical-to-electrical conversion [144]. These gates allow data to be processed at the time light takes to propagate through the device length, thereby offering the potential for ultra-fast operation. Meanwhile, optical switches enable the routing and management of optical data flow within these networks, ensuring that information is efficiently handled without needing to resort to costly optical–electrical–optical (O-E-O) conversions [145]. Together, these components form the backbone of all-optical signal processing systems, which operate entirely in the optical domain and avoid the performance degradation associated with O-E-O conversion. While single-mode waveguides have long supported on-chip optical systems due to their simplicity and low crosstalk, they face fundamental limitations in energy efficiency, functional scalability, and integration density. First, electro-optical switches in single-mode systems have a typical power consumption of around 100–500 fJ/bit [146], which becomes unsustainable in large-scale core networks [147]. Second, functional diversity is limited, as single-mode architectures primarily rely on two multiplexing dimensions, i.e., wavelength and polarization, constraining the complexity of on-chip signal processing. Third, integration scalability is challenged by the need to deploy multiple devices in parallel, which increases system size. In contrast, multimode waveguides offer a compelling alternative by enabling three-dimensional multiplexing through the additional degree of freedom given by spatial multimodality, greatly enhancing interconnect density and supporting multiple signal paths in a compact footprint [148]. Nonlinear multimode waveguides, in particular, are emerging as versatile platforms for all-optical signal processing. Although their nonlinear efficiency per mode is typically lower compared to single-mode devices due to the larger mode areas involved, the presence of multiple spatial channels enables intermodal nonlinear interactions, relaxed phase-matching conditions, and mode-selective functionalities. These advantages make them well-suited for parallel logic operations, energy-efficient switching, and functionally rich photonic systems. Rather than replacing highly optimized single-mode devices, nonlinear multimode waveguides offer a complementary pathway toward scalable, low-power, and multifunctional optical networks.

The concept of optical logic gates first emerged in the early 1970s and is akin to how electronic transistors are used in conventional computing. The primary appeal of optical logic lies in its potential for much higher processing speeds, due to the broader bandwidth of optical systems. Significant progress was made in the late 1990s and early 2000s with key advancements in nonlinear optical materials. Semiconductor optical amplifiers (SOAs) [149], [150], [151], [152] and highly nonlinear fibers (HNLFs) [153], [154], [155] were among the first platforms to be employed, enabling the implementation of all-optical logic gates such as AND, OR, XOR, NOT, and NAND. These gates operate by exploiting nonlinear effects such as FWM, cross-gain modulation (XGM), TPA, cross-phase modulation (XPM), and self-phase modulation (SPM). The mid-2000s marked a turning point with the realization of optical logic gates in microscale or even nanoscale waveguides, including silicon [156], [157], [158], PPLN [159], [160], [161], and chalcogenide waveguides [162], [163], alongside the use of photonic crystal structures [164], [165], [166]. These material platforms have enabled the realization of more compact and efficient optical circuits offering the prospect of massive integration, and pushing the concept of all-optical logic gates closer to practical implementation.

Among the available material platforms, SOI stands out due to its high nonlinearity, complementary metal-oxide-semiconductor (CMOS) compatibility, and ability to exploit key nonlinear effects such as SPM, XPM, and FWM. Its high index contrast also enables the development of compact multimode devices, which are essential for implementing MDM. Moreover, multimode devices offer a cost-effective solution for enhancing the parallelism of all-optical logic gates without significantly increasing their footprint, providing a distinct advantage over traditional single-mode waveguide devices. Additionally, by exploiting the FWM effect, logic gates can be created by encoding information in the amplitude and/or phase of the interacting waves. This approach is particularly effective for implementing logic functions like AND, XOR, and XNOR, since it generates new frequency components that directly represent the desired logical operations. Benefitting from the similarity between wavelength conversion and FWM-based logical operations, mode-selective wavelength conversion in multimode silicon waveguides [167] has become a popular method for realizing all-optical logic gates.

**Figure 6:**
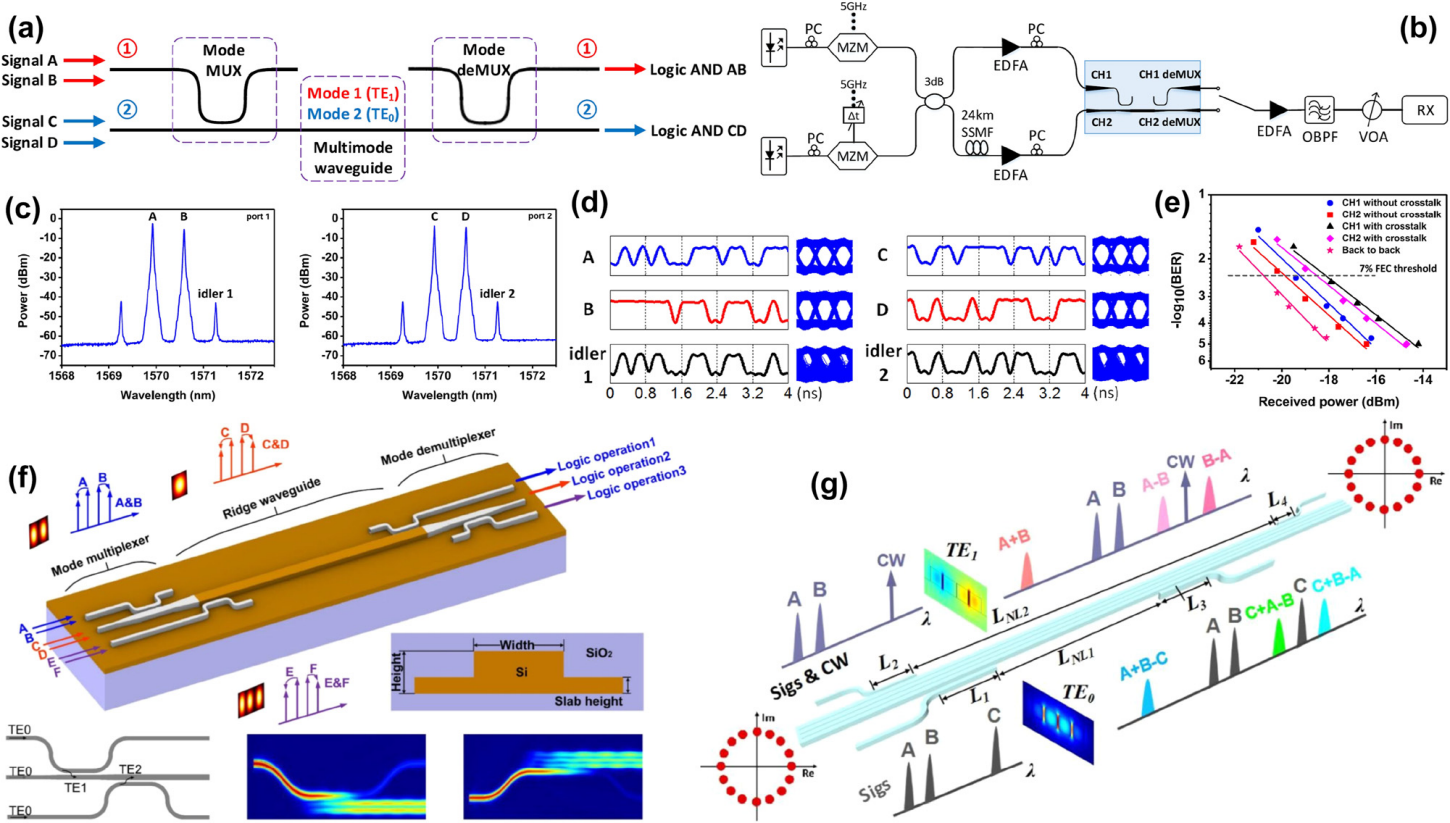
All-optical logic operations using nonlinear multimode waveguides. (a) Schematic layout of the device proposed in ref. [168] which enables the realization of dual-channel AND logic operation utilizing the fundamental TE_0_ mode and the higher-order TE_1_ mode of the multimode silicon waveguide; (b) experimental setup used for the dual-channel AND logic operations on modulated signals (PC: polarization controller; MZM: Mach-Zehnder modulator; SSMF: standard single mode fiber; EDFA: Erbium doped fiber amplifier; CH1/2: channel 1/2; OBPF: optical band pass filter; VOA: variable optical attenuator; RX: receiver); (c) example spectra obtained using intramodal FWM in the multimode silicon waveguide at the output (left) port 1 and (right) port 2 under simultaneous AND logic operation; (d) time-domain waveforms of A, B and AND product-idler at output port 1 (left) and of C, D and AND product-idler at output port 2 (right), with the corresponding eye diagram measurements; (e) BER measurements as a function of the received power for the back to back (B2B) case, single-channel (without crosstalk) and dual-channel (with crosstalk) AND logic operations for the two channels. (f) Layout and working principle of the device proposed in ref. [169], with details of the multimode waveguide cross-section, mode multiplexer and 3D finite-difference time-domain (FDTD) simulations for the two designed ADCs for TE_1_ and TE_2_ mode multiplexing. (g) Layout and working principle of the device proposed in ref. [170] used to perform 1.28 Tb/s all-optical multimode hexadecimal addition and subtraction. (a)–(e) Panels are reproduced with permission from ref. [168] ©2016 IEEE. (f) Panel is reproduced from ref. [169] under a CC BY 4.0 license. (g) Panel is reproduced from ref. [170] under a CC BY 4.0 license.

In 2017, Wang et al. [168] demonstrated a dual-channel all-optical AND logic gate based on FWM in a multimode silicon strip waveguide. The waveguide was designed with dimensions of 680 nm in width and 220 nm in height to place the zero dispersion wavelength close to 1,550 nm, which is critical for maximizing FWM efficiency around this wavelength. The device layout, which is shown in Figure 6(a), included tapered directional couplers at both the input (Mode MUX) and output (Mode deMUX) sections, enabling mode conversion and (de)multiplexing functionalities. Specifically, the waves in the fundamental TE_0_ mode coupled into input port 1 (signal A and B, channel 1) were converted in the higher-order TE_1_ mode of the multimode waveguide by the Mode MUX, while the waves in the fundamental TE_0_ mode coupled into input port 2 (signal C and D, channel 2) remained in the fundamental TE_0_ mode of the multimode waveguide. After the nonlinear FWM process in the multimode silicon waveguide, idler components conveying the AND logic result for each channel (Logic AND AB for channel 1 in the TE_1_ mode and Logic AND CD for channel 2 in the TE_0_ mode) were sent to different output ports of the output Mode deMUX, depending on their spatial mode. As a proof of concept experiment, the authors used the experimental setup reported in Figure 6(b) to experimentally demonstrate two parallel 5 Gb/s AND logic operations using on-off keying (OOK) signals, resulting in a total aggregate logic capacity of 10 Gb/s. Figure 6(c) reports the spectra of the two signals (A and B for channel 1, C and D for channel 2) as well as those of the two generated idlers measured at output ports 1 (left panel) and 2 (right panel), respectively, when the AND logic function was performed in parallel on the two channels. The temporal waveforms and measured eye diagrams of the signals and the generated idler for channels 1 (left panel) and 2 (right panel) are shown in Figure 6(d). The comparison of the temporal waveform sequences of the input signals and the generated idlers confirm the dual-channel AND logic operations. Figure 6(e) reports the BER results for the two idlers measured at output port 1 (CH1, TE_1_ mode) and port 2 (CH2, TE_0_ mode), under dual-channel AND logic operations with the signals propagating in parallel (i.e., with crosstalk) and single-channel AND logic operation with the signals propagating in each channel individually (i.e., without crosstalk). The authors measured power penalties of 0.9 and 1.5 dB for the TE_0_ and TE_1_ modes, respectively, relative to the B2B case in the absence of mode crosstalk, at the 7 % forward error correction (FEC) threshold, while additional power penalties of 1.3 and 1.1 dB were measured when mode crosstalk was present. These results highlight the potential of multimode silicon waveguides for compact, high-capacity optical logic devices, with scalability to support more complex logic functions and a greater number of channels. It is worth noting that the demonstrated dual-mode AND logic gate enables parallel operation within a single multimode waveguide, thereby aggregating the data throughput within a compact footprint, contrasted to the use of multiple single-mode waveguides for the same operation. However, this demonstration was still limited by the low data rate and the small number of channels, primarily due to the low conversion efficiency (about −40 dB), which was caused by significant scattering losses from waveguide sidewall roughness, as well as TPA- and FCA-induced nonlinear losses in the silicon waveguide.

The performance was further improved by Hu et al. [169], who utilized a greater number of modes to realize AND logic functions using a triple-mode silicon rib waveguide. This waveguide geometry was selected for its lower losses compared to strip waveguides and its ability to reduce mode crosstalk while performing AND logic operations via FWM. The rib waveguide dimensions were optimized with a rib thickness of 220 nm, a 70 nm slab height and a 1,412 nm waveguide width. This design supported three spatial modes (TE_0_, TE_1_, and TE_2_), with low losses of 0.6, 0.75 and 0.9 dB/cm, respectively, resulting in a conversion efficiency higher than −20 dB for all three modes. Proper waveguide design, along with low loss for all spatial modes, large bandwidth and strong nonlinear interaction ensured that high performance could be achieved. The device design, which is shown in Figure 6(f), included two ADCs at both the input and output, enabling mode conversion between TE_0_ and TE_1_ modes, as well as between TE_0_ and TE_2_ modes. Using this configuration, the authors were able to demonstrate three-channel logic operations within a single multimode silicon waveguide. Thanks to the low losses and relatively high conversion efficiency, they successfully demonstrated high-quality AND logic operations at a significantly higher data rate of 3 × 40 Gbit/s, with fully opened eye diagrams for the idler components generated in the three spatial modes. It should be appreciated that to achieve the same aggregate throughput with single-mode devices, approximately three times the footprint would have to be sacrificed.

Another advantage of using a silicon rib waveguide geometry is that it allows the inclusion of a pn-junction in the silicon slab to reduce FCA-related losses, enabling higher pump power injection and, hence, increasing the conversion efficiency. However, as previously mentioned, it is important to notice that using higher-order modes for FWM results in lower conversion efficiency due to a typically larger effective area compared to the single-mode case. This makes the use of spatial modes as a degree of freedom for scaling up parallel logic operations more challenging. Achieving greater nonlinear Kerr index coefficients, which could improve conversion efficiency for higher-order modes, remains difficult when relying solely on silicon. This limitation may also hinder the implementation of more complex logic operations using advanced modulation formats, which, while improving spectral efficiency, demand higher OSNR.

To address these challenging issues, Wang et al. [170] proposed and numerically demonstrated a highly nonlinear organic-silicon slot waveguide (HN-OSSW) designed for high-capacity, multi-channel all-optical logic operations. The layout of the device is shown in Figure 6(g). The nonlinear polymer MEH-PPV, which offers a 40-fold increase in the nonlinear Kerr coefficient *n*_2_ compared to that of silicon, was incorporated into the slot. This configuration significantly enhanced the nonlinear coefficients for the TE_0_ and TE_1_ modes, reaching values of 7,078 and 5,858 W^−1^m^−1^, respectively. The HN-OSSW was optimized to support efficient FWM with minimal intermodal crosstalk, making it suitable for MDM systems. The device design featured a multimode slot waveguide which could be fabricated using standard CMOS-compatible processes, where the TE_0_ and TE_1_ modes were excited and manipulated through directional couplers that served as mode multiplexers and de-multiplexers. Numerical simulations showed that the HN-OSSW can perform multimode all-optical hexadecimal addition and subtraction for 16 phase-shift keying (16-PSK) signals with an aggregate data rate of 1.28 Tb/s. In addition, six different logic operations (A + B − C, A + C − B, B + C − A, A + B, A − B and B − A, with A, B and C being input signals) could be simultaneously performed, thanks to the high number of FWM products generated by the strong nonlinear interaction in the device. The optimized phase-mismatch conditions and efficient light confinement within the slot waveguide ensured high FWM conversion efficiency, with logic operation results showing error vector magnitude (EVM) penalties lower than 5.8 dB at an input OSNR of 30.3 dB. It is worth noting that the proposed configuration outperforms the single-mode silicon strip waveguide implementation by achieving a 40 times enhancement in nonlinearity (via the MEH-PPV polymer) and enabling multimode operation with minimal crosstalk. It supported 1.28 Tb/s transmission and simultaneously performed six different logic operations across two distinct modes. This design benefited from a combined high FWM efficiency and scalable multi-channel processing, effectively overcoming the capacity and functional limitations of single-mode devices.

In addition to the development of all-optical logic gates based on nonlinear multimode waveguides, significant interest has also been devoted towards the realization of all-optical switches, which can potentially provide reconfigurable functionalities. All-optical switches have emerged as key enablers in optical interconnects since they allow for data flow reallocation and enhanced information throughput. Research on reconfigurable optical switches has made remarkable progress, particularly in the domain of on-chip silicon-based switches, where MZI and microring resonator structures are used for wavelength selectivity. The port counts of single-mode optical switches have scaled up to 240 × 240 [171]. Recently, MDM techniques have been introduced to extend these capabilities to multimode operations. For instance, a mode-insensitive 2 × 2 three-mode optical switch was developed using a pair of MMI 3-dB couplers and a widened phase shifter, designed to provide consistent thermal sensitivity across the first three modes [172]. Following another approach, multimode signals were de-multiplexed into single-mode signals, which were then processed separately by single-mode optical switches [173]. A more compact device was later proposed based on the use of microring resonators by introducing mode exchangers to realize de-multiplexing-free operation [174]. Despite significant advancements, MZI-based optical switches suffer from large device footprints, especially when using multimode waveguides. Nevertheless, even if microring resonator-based devices allow realizing more compact structures, their switching speeds are limited by free carrier recovery times and thermal sensitivity, which can range from picoseconds to microseconds depending on the configuration. These constraints, combined with the need for electrical power to drive the switching mechanism, have spurred the exploration of alternative approaches, such as the use of nonlinear optical effects, particularly in multimode waveguides. One promising approach involves tuning the phase difference between different modes through nonlinear phase modulation, enabled by a high-power pump. By leveraging multimode interference effects, such as those based on intermodal cross-phase modulation (iXPM), it is possible to achieve efficient all-optical switching. This bypasses the need for electro-optical conversion, making the system more compact, faster and, potentially, more energy-efficient. Furthermore, utilizing nonlinear materials with high nonlinearity can significantly reduce the device footprint by providing strong nonlinear phase modulation over a short effective interaction length.

**Figure 7:**
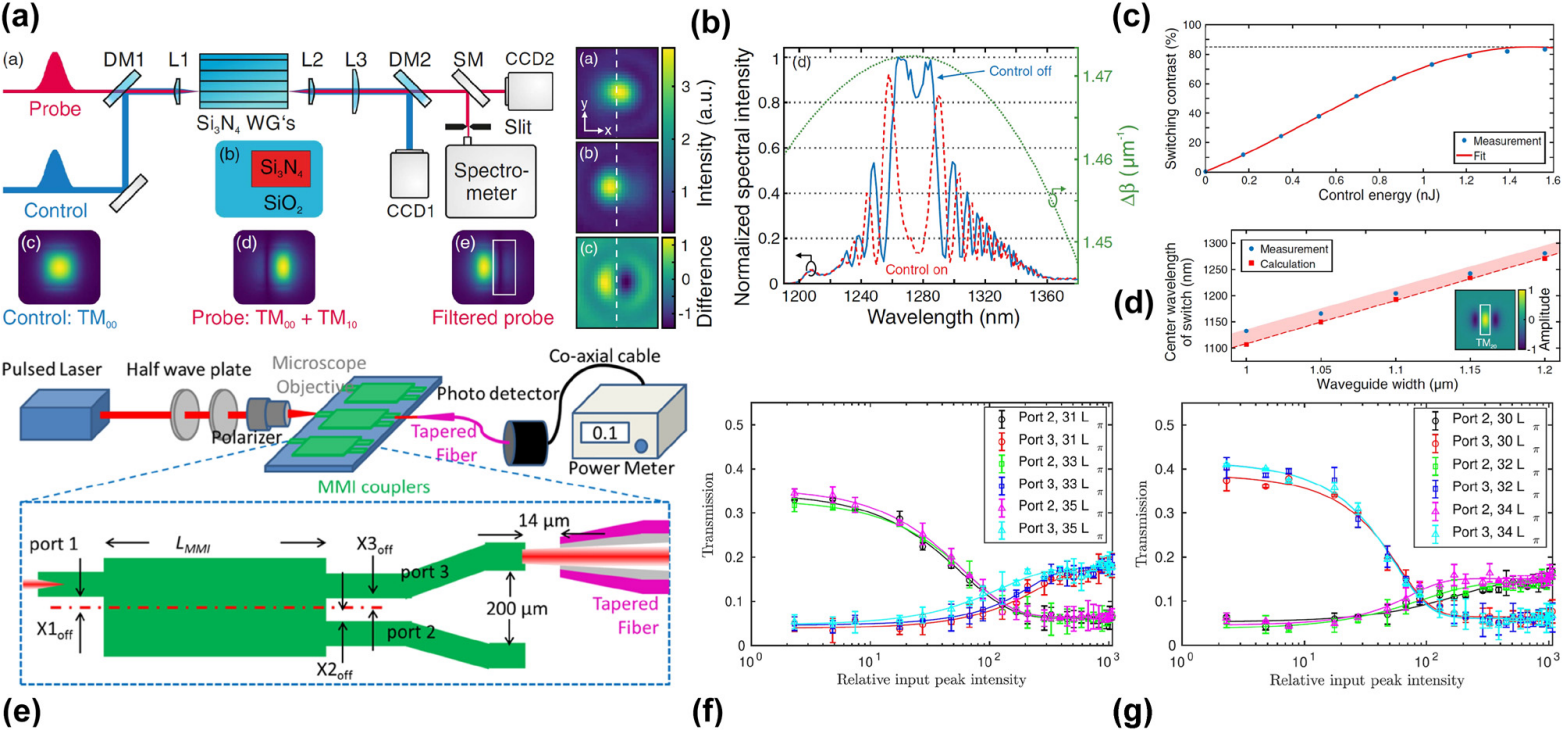
All-optical switching experiments exploiting nonlinear multimode waveguides. (a) Setup used for the all-optical switching experiments based on a nonlinear SiN multimode waveguide presented in ref. [175], with the waveguide cross-section, intensity distributions of the control and probe beams and spatial profile of the transmitted probe beam with a white rectangle indicating the spatial filtering applied by the slit (filtered probe). DM: dichroic mirror; L: lens; WG: waveguide; SM: switchable mirror; CCD: charge-coupled-device camera. (b) (left) Probe beam profiles at the waveguide output acquired by the CCD2 camera (bandpass filtered at 1,280 nm) with the control beam turned off (top) and on (middle) and the difference between the two images (bottom). (right) Normalized spectral intensity measured after the slit with (dashed red curve) and without (solid blue curve) the presence of the control beam and numerically simulated variation of the difference of propagation constants Δ*β* of the two probe modes (dotted green line) as a function of wavelength [175]. (c) Experimentally measured switching contrast versus coupled control beam energy and (d) dependence of the center wavelength of the switch on the SiN waveguide width when using the TM_20_ instead of the TM_10_ mode as the second probe mode. The calculated field distribution of the TM_20_ mode is displayed in the inset, with the filtering slit represented by a white rectangle [175]. (e) Experimental setup used to perform the all-optical switching experiments based on the nonlinear Ta_2_O_5_ MMI coupler reported in ref. [176] and schematic top view of the device. (f) Experimentally measured and calculated transmission curves at output ports 2 and 3 as a function of the relative input peak intensity for lengths of the MMI couplers equal to odd multiples of *Lπ* and (g) even multiples of *Lπ* [176]. (a)−(d) Panels are reproduced with permission from ref. [175] ©2018 Optica Publishing Group. (e)–(g) Panels are reproduced with permission from ref. [176] ©2023 American Chemical Society.

In 2018, Lüpken et al. explored the utilization of SiN waveguides for all-optical switching, leveraging its relatively high nonlinear refractive index (*n*_2_ ≈ 2.5 × 10^−19^ m^2^/W) and broad transparency window [175]. The SiN multimode waveguides used in this work were designed with a height of 0.93 μm and widths ranging from 0.7 to 1.2 μm to support multiple transverse modes. The fundamental TM_00_ mode and the higher-order TM_10_ mode were employed in the experiments performed using the setup reported in Figure 7(a). The working principle of the all-optical switch was as follows: a probe beam was coupled into an equally distributed superposition of the TM_00_ and TM_10_ modes of the SiN multimode waveguide, while, simultaneously, a second control beam at a different wavelength was coupled into the TM_00_ mode to shift the phase between the two probe modes through iXPM. This was possible because the TM_00_ mode of the probe beam acquired a greater phase shift compared to the TM_10_ probe mode due to the higher intensity overlap with the TM_00_ control mode. The authors then performed a spatial filtering of the interference pattern obtained from the two probe modes as a phase-sensitive discriminatory to realize the all-optical switching operation. This was experimentally realized by filtering the probe beam’s output profile with a slit and measuring the transmitted light with a spectrometer. The measured normalized spectral intensity for the control beam on and off is reported in Figure 7(b), showing the switching behaviour of the device. The switching contrast at the center wavelength of ≈1,280 nm was measured as a function of the coupled control beam energy, with the results reported in Figure 7(c), showing the capability to achieve a switching contrast of 83 % with an energy of only 1.6 nJ. This represents a significant reduction in required power compared to similar devices based on optical fibers.

Furthermore, the spectral range of switching (3 dB range) was measured to be equal to 4.2 THz, more than an order of magnitude broader than switches based on resonant structures [177]. The figure of merit (FoM) for all-optical switches, defined as the product of the necessary control peak power times the required waveguide length [178], was found to be 2.3 Wm, a 2,000-fold improvement over previous experiments using transverse modes in graded-index fibers [179]. These results highlight the potential of SiN waveguides for low-power, broadband all-optical switching. The significant reduction in power requirements and broad working frequency range make this approach particularly appealing for applications in optical interconnects, data centers, and high-speed telecommunications, where space and energy efficiency are critical. The authors also demonstrated the possibility of tuning the operation wavelength of the switch, which can be achieved by using different probe spatial modes and/or waveguide geometries. Figure 7(d) shows the variation of the center wavelength of the switch as a function of the waveguide width (with a fixed height of 0.93 μm) when using the TM_20_ instead of the TM_10_ mode as the second probe mode.

In ref. [176], Lin et al. chose tantalum pentoxide (Ta_2_O_5_) as the material platform for all-optical signal processing applications, exploiting its high Kerr nonlinear coefficient (*n*_2_ ≈ 1 × 10^−18^ m^2^/W) and its wider bandgap (4.05–4.18 eV [123], [180]) compared to silicon and SiN, which allows achieving negligible nonlinear losses in the visible and near-infrared spectrum, thus improving the device efficiency at these wavelengths. The researchers demonstrated an all-optical switching mechanism in a low-power optically-controlled Y-junction device based on a nonlinear MMI coupler with a thickness of 100 nm, a width of 8 μm and a length of 4.3 mm. The setup used for the experimental characterization and the device layout are schematically shown in Figure 7(e). A mode-locked laser emitting 1 ps pulses at 1,030 nm (repetition rate of 18 MHz) was used as the optical source and a combination of a half-wave plate and a polarizer was employed to control the power coupled into the device. At the device output, the optical power emitted either from port 2 or port 3 of the MMI was collected using a tapered fiber and measured. When optical pulses were coupled into port 1 of the device, a certain modal distribution in the MMI was excited and the laser pulses could be redirected by the modal interference into output port 2 or 3 depending on the pulse power, thereby allowing to achieve all-optical power-dependent switching functionality. Figure 7(f) and (g) shows the measured and numerically calculated transmission curves at output ports 2 and 3 as a function of the relative input peak intensity for nonlinear MMI couplers with lengths equal to odd and even multiples of *L*_*π*_, respectively. *L*_*π*_ represents the characteristic length that causes the accumulated phase difference between the TE_00_ and TE_10_ modes to be *π*, where TE_00_ and TE_10_ were the two main guided modes in the MMI. Considering the case where the MMI lengths were equal to odd multiples of *L*_*π*_, it is possible to see from Figure 7(f) that the transmission at port 2 decreased while increasing the incident peak power, with the opposite behaviour recorded at port 3. For MMI lengths equal to even multiples of *L*_*π*_, the opposite trend was observed, as shown in Figure 7(g). In addition to the demonstrated all-optical switching functionality, the proposed configuration holds promise for the realization of other nonlinear integrated photonic functional devices such as saturation absorbers and optical power limiters by properly designing the multimode interaction in the MMI section.

The works reviewed in this section have demonstrated that nonlinear multimode waveguides offer a powerful platform for enabling both all-optical logic gates and all-optical switches, leveraging their ability to support multiple modes and nonlinear interaction combinations. For all-optical logic gates, leveraging multiple spatial modes (e.g., TE_0_, TE_1_, TE_2_, …) enables parallel logic operations within a single waveguide, significantly boosting aggregate throughput and area efficiency. For example, as previously reported, these devices can support simultaneous multi-channel processing at data rates up to 1.28 Tb/s while performing multiple distinct logic functions concurrently. Furthermore, integrating highly nonlinear materials like MEH-PPV into hybrid organic-silicon slot waveguides can enhance FWM efficiency by a factor of 40, overcoming silicon’s inherent Kerr nonlinearity limitations. However, challenges remain. Higher-order modes typically suffer from lower FWM efficiency due to their larger effective areas compared to the single-mode case, and mode crosstalk can degrade signal quality. To fully exploit multimode waveguides for high-capacity, parallel and multifunctional all-optical processing, integrating advanced nonlinear materials is key. Platforms like ultra-low-loss SiN [181] and highly nonlinear III–V semiconductors [182] could offer improved FWM performance. Leveraging these materials in multimode designs could greatly enhance the scalability and functionality of all-optical logic systems.

In addition, nonlinear multimode waveguide-based all-optical switches offer several advantages over single-mode devices, particularly in enabling compact, high-speed, and energy-efficient systems. By exploiting iXPM and multimode interference, these switches allow for phase-sensitive control without the need for electro-optic conversion, offering faster response times and reduced energy consumption. Additionally, the ability to use multiple spatial modes enables parallel switching and broader spectral operation within a single waveguide, reducing footprint and improving integration density. Material platforms like SiN and Ta_2_O_5_ further enhance performance by offering low losses and broad transparency windows, leading to reduced control power requirements and wider bandwidths. Still unresolved challenges include mode-dependent dispersion and limited spatial overlap which can limit the achievable switching contrast. Mode crosstalk and fabrication-induced variations in waveguide geometry also affect stability and reproducibility. Furthermore, precise spatial mode excitation and filtering often require complex input/output coupling or free-space alignment strategies. To address these limitations, possible solutions may include optimized waveguide designs to tailor modal dispersion and confinement, the use of broadband and fabrication-tolerant mode converters, and advanced nonlinear materials with high Kerr coefficients and negligible absorption. Continued innovations in device architecture, such as the design of de-multiplexing-free microring-based systems and nonlinear MMI couplers, also show strong promise in advancing multimode all-optical switching toward scalable and reconfigurable photonic systems.

## 4 Supercontinuum generation

SC radiation represents a powerful tool for many applications ranging from optical communications to metrology and spectroscopy, only to name a few [4]. Extensive research into the generation of SC beams started in the 2000s, mainly favoured by the advent of micro-structured photonic crystal optical fibers, which made available to researchers an optical medium with unprecedented nonlinear features [183]. The most investigated technique for obtaining SC radiation exhibiting a wide optical spectrum relies on the propagation of ultrashort pulses in a highly nonlinear regime [184]. The ability to control spectral broadening and flatness is a key challenge, and it essentially depends on the material nonlinearity and the precise engineering of the spatial confinement and dispersion of the chosen optical waveguide. For these reasons, CMOS-compatible integrated platforms quickly emerged as ideal candidates for the development of compact, low-cost SC sources with excellent spectral quality. More recently, the growing interest in multimodal systems has introduced a new degree of freedom, enabling nonlinear processes involving multiple spatial modes with distinct dispersive properties. In this context, the use of integrated platforms provides powerful modal manipulation techniques, offering the possibility of selectively exciting and controlling highly confined modal distributions. In most of the published works, the generation of SC radiation is ruled by the spectral dynamics of higher-order soliton pulses. When such pulses propagate in the anomalous dispersion regime near the zero of the GVD, they undergo a strongly perturbed propagation where a number of nonlinear effects such as SPM, FWM, pulse self-steepening, Raman scattering and soliton fission simultaneously take place, imposing a massive broadening of the initial spectrum. In addition, higher-order soliton dynamics induce a release of energy to a broadband DW that can grow coherently along propagation, contributing to SC spectral broadening and flattening. Energy transfer to DW is effective if a phase-matching condition between the phase of the soliton pulse and that of the dispersive radiation itself is satisfied [185]. The phase mismatch Δ*β* can be described by the following relation [186]:

(5) $$\Delta \beta \left(\omega \right)=\beta \left(\omega_{\text{DW}}\right)-\beta \left(\omega_{s}\right)-\beta_{1}\left(\omega_{\text{DW}}-\omega_{s}\right)-\frac{1}{2}\gamma P_{s}$$

where *β*(*ω*) is the frequency-dependent propagation constant for the considered waveguide modal distribution, *γ* is the Kerr nonlinear coefficient, and *P*_*s*_ is the peak power of the soliton pulse. *ω*_DW_ and *ω*_*s*_ are the angular frequencies of the DW and soliton, respectively. The spectral position of the phase-matching frequencies is governed by the combination of even and odd higher-order dispersion coefficients that may generate more than two DW peaks depending on their signs and absolute values [187]. SiN on insulator is one of the most studied platforms for SC generation thanks to the wide spectral transparency offered by the platform, ranging from the visible to the MIR. In addition, recent advancements in fabrication have made thicker waveguides available (even thicker than 1 μm), thus triggering the investigation of multimode nonlinear interactions and propagation. In an early work, Epping et al. designed and fabricated a SiN waveguide with a D-shape core and a SiO_2_ cladding through a low-pressure chemical vapor deposition (LPCVD) process [188]. The use of a large waveguide cross-section (1 μm thickness, different widths around 0.8 μm were considered) allowed to shift the zero dispersion wavelength to values as short as 1 μm enabling operation in the anomalous dispersion regime and phase-matched DW excitations with a pump wavelength at 1.064 μm. Due to the large dimension, the multimode waveguide supported three modes in the TM polarization, namely the fundamental TM_00_ and the two higher-order modes TM_10_ and TM_20_. By coupling ultrashort pulses (115 fs) emitted by a mode-locked Yb-doped fiber laser (repetition rate of 41 MHz, maximum average output power of 200 mW), an ultra-wideband SC radiation extending from 470 nm to 2,130 nm was obtained. Nevertheless, numerical calculations performed through multimode nonlinear Schrödinger equations demonstrated that only the fundamental and the TM_20_ modes were actually excited in the waveguide. In addition, SC generation and broadening were only due to the fraction of power propagating in the fundamental mode. Indeed, the authors verified that the TM_20_ mode underwent normal dispersion in the waveguide. The results of this work outlined two main issues: the importance of the spatial control of the input coupling and the need for a fine-tuning of the dispersion properties for all the modes involved in the nonlinear process.

**Figure 8:**
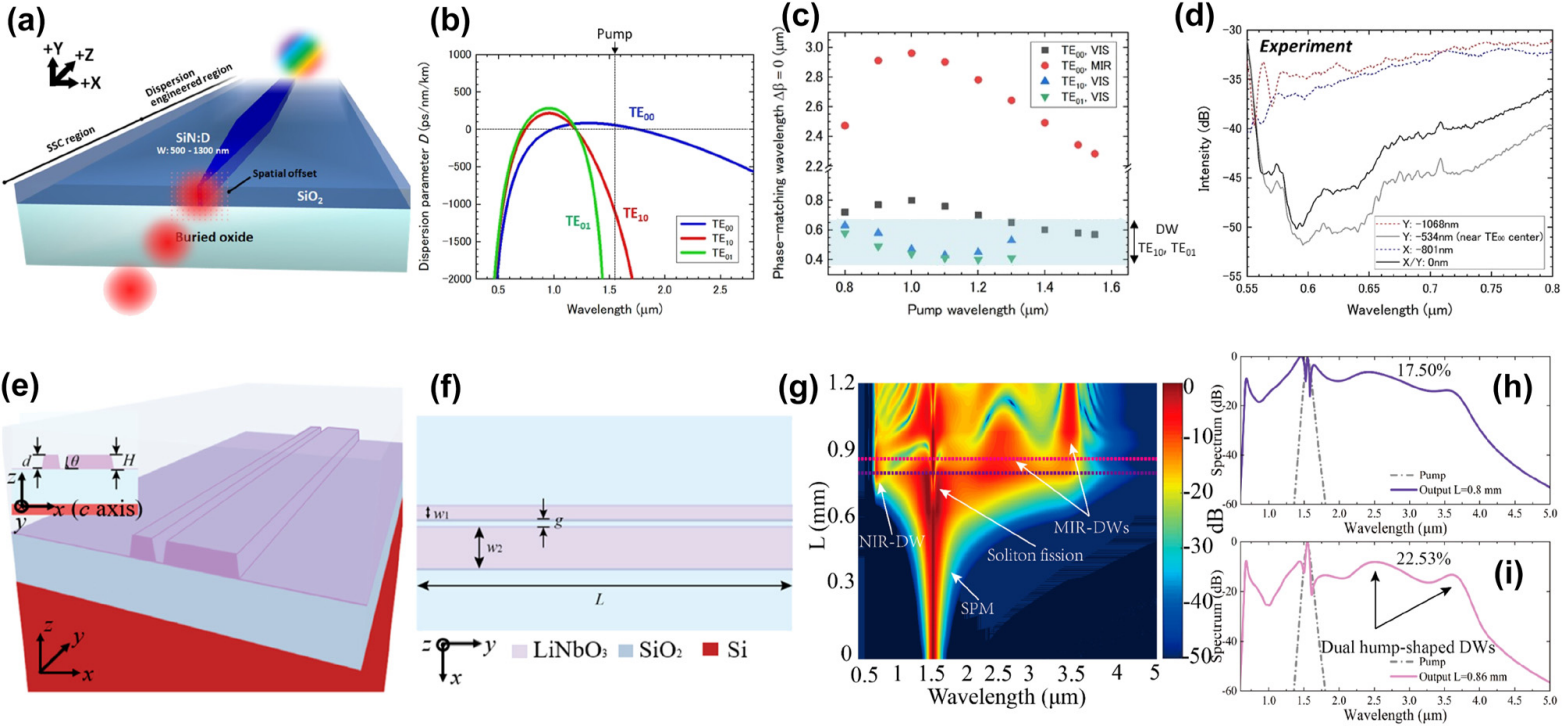
SC generation in nonlinear multimode waveguides. (a) Scheme of the SiN:D waveguide for SC generation including an adiabatic mode converter demonstrated in ref. [186]; (b) calculated dispersion parameter *D* as a function of wavelength for different spatial modes (TE_00_, TE_10_ and TE_01_); (c) phase-matching wavelength for DW generation as a function of the pump wavelength for all the modes supported by the waveguide. The horizontal blue shaded area on the graph highlights the role of higher-order modes for SC generation in the visible (VIS) through phase-matched DWs; (d) experimental results on SC generation in the visible for different spatial position offsets of the incident focusing beam in the *X*- and *Y*-axes to control the power coupling into different spatial modes. (e) 3D schematic diagram and (f) 2D top view of the dual-coupled LiNbO_3_ waveguide proposed by Jia et al. [189] (inset of panel (e): cross-section view of the dual-coupled waveguide); (g) spectral evolution of the higher-order soliton as a function of the propagation length *L*; (h) optical spectra of the pump and the output at a propagation length *L* = 0.8 mm and (i) at *L* = 0.86 mm. (a)–(d) Panels are reproduced with permission from ref. [186] ©2023 Optica Publishing Group. (e)–(i) Panels are adapted from ref. [189] under a CC BY 4.0 license.

Effective multimode contribution to SC generation was demonstrated by Kou et al. [186]. A dispersion-engineered deuterated SiN (SiN:D) waveguide was designed to control the dispersion relation of all the involved modes within the anomalous dispersion regime and a SiO_2_ planarized top-cladding was employed to further decrease the mode confinement and increase the absolute anomalous dispersion value. As shown in Figure 8(a), an adiabatic spot-size converter (SSC) was fabricated at the edge of a straight waveguide and the degree of excitation of each spatial mode could be controlled by adjusting the spatial incidence position of the pump light with a precision of a few hundreds of nanometers. Figure 8(b) reports the numerically simulated dispersion properties for each supported mode (TE_00_, TE_10_ and TE_01_), showing that the fundamental mode experiences anomalous dispersion around 1.55 μm, whereas higher-order modes propagate in the anomalous dispersion regime at shorter wavelengths. Figure 8(c) shows the numerically simulated phase-matching conditions for DW generation as a function of the pump wavelength for each considered spatial mode. It can be easily observed that TE_10_ and TE_01_ modes can contribute to extending the SC radiation in the visible thanks to phase-matched DWs, as outlined by the horizontal blue shaded area on the graph. The waveguide was pumped at 1.55 μm through a passively mode-locked Er-doped fiber laser with a 74 fs pulse duration. As predicted, in this spectral range only the TE_00_ mode exhibited anomalous dispersion. According to numerical simulations, a new virtual radiation below 1.2 μm could be generated by the SPM effect of the TE_00_ mode at 1.55 μm, which, in turn, excited higher-order solitons in the TE_10_ and TE_01_ modes by a cascaded pump mechanism. Figure 8(d) reports the experimental evidence of SC flattening and broadening in the visible range when higher-order modes were properly excited by finely tuning the spatial incidence position in the *X* and *Y* directions at the input cross-section. It can be observed that the spectral intensity in the 0.5–0.8 μm range increased by up to 15 dB (dotted lines) when also the TE_10_ and TE_01_ modes contributed to the SC radiation.

SC generation based on multimode nonlinear propagation has been investigated in several integrated platforms, which enabled access to different spectral regions thanks to their distinctive transparency bands, nonlinear and dispersive properties. Ta_2_O_5_ channel waveguides, which are characterised by a higher nonlinearity compared to SiN, were exploited in ref. [190] to generate SC radiation assisted by DW resonance for several TE and TM modes with a spectral extension larger than 600 nm when pumping in the TM_10_ mode. Aluminum nitride (AlN) is also a promising platform thanks to its broadband transparency in the visible and UV range. In ref. [191], SC generation was obtained in a multimode AlN waveguide fabricated on a sapphire substrate where the TE_10_ mode was selectively exploited to guarantee propagation in the anomalous dispersion regime at 800 nm, leading to SC generation extending from 490 to 1,100 nm.

The phase-matching condition between solitons and DWs can also be achieved through more complex manipulations of the modal propagation regime, enabled by the design and fabrication capabilities offered by integrated optical platforms. In 2018, Hickstein et al. proposed a QPM mechanism via periodic modulations of a waveguide structure to control the evolution of SC generation in the TE_20_ and TE_00_ waveguide modes [192]. The authors reported two different schemes in a SiN platform based on waveguide width modulation and cladding modulation, respectively. Periodic modulation of the waveguide structure has a direct impact on the modal area, inducing a periodic variation of both the nonlinear coefficient *γ* and GVD. This provides an additional contribution to the phase mismatch (Eq. (5)) ensuring a QPM condition for DW enhancement. The authors demonstrated that by using this technique it was possible to enhance the intensity of the SC generation in numerous locations across the spectrum.

DW generation induced by SC has also been widely investigated for the development of MIR sources for optical spectroscopy [193], [194]. As discussed before, the MIR spectral region has great relevance for spectroscopy and sensing with applications in health and environmental monitoring. In particular, the 3–5 μm region is important for air monitoring as it contains the molecular fingerprint of several pollutants typically present in the air. Various advances in technologies based on direct generation through QCLs and ICLs have been done in the past years [143], [195]. A promising and flexible alternative is to implement wavelength conversion in nonlinear materials in order to reach and fully cover the MIR spectral range. The main idea is to leverage the advances in technologies developed for communications and fiber lasers and translate their emission wavelength in the MIR region. Modal manipulation offers an additional tool to satisfy the phase-matching condition in this specific spectral region for DW enhancement. In this context, Jia et al. proposed and numerically demonstrated a new approach for DW generation in the MIR based on an *x*-cut LiNbO_3_ on insulator platform [189]. The authors designed a dispersion-engineered structure based on a dual-coupled ridge waveguide, whose 3D schematic diagram and 2D top view are shown in Figure 8(e) and (f), respectively. The structure consisted of two parallel ridge waveguides divided by a thin gap (the gap parameter g varies from 200 to 400 nm) obtained by etching the LiNbO_3_ thin film. The structure supported the propagation of supermodes distributed in both cores that were finely engineered as a function of the geometrical parameters (*d*, *H*, *w*_1_, *w*_2_ and *g*). One of the supermodes, namely the anti-symmetric mode, produced additional zero-dispersion wavelengths in the MIR region and, consequently, multiple regions for DW emission. Using 1.55 μm wavelength pulses with a duration of 50 fs and a peak power of 4.5 kW, the authors predicted soliton evolution in a 0.86 mm long waveguide, resulting in a dual hump-shaped MIR-DW with a conversion efficiency of up to 22.53 %. The detailed soliton spectral evolution along the propagation length is shown in Figure 8(g). In the first part of the waveguide, spectral broadening is mainly due to SPM. At a propagation length *L* ≈ 0.7 mm, soliton fission takes place with the generation of three DW peaks, one in the NIR and two in the MIR at around 2.5 and 3.6 μm. The corresponding output spectra, as simulated numerically at two values of the propagation length *L =* 0.8 and 0.86 mm, are reported in Figure 8(h) and (i), respectively. A similar approach was also proposed in ref. [196], where the selective excitation of the antisymmetric supermode of a SiN dual-core waveguide was assisted by an input Mach-Zehnder circuit. The use of coupled nonlinear waveguides was also reported in other works by the same research group, where Xia et al. experimentally demonstrated on-chip control of spatially multimode nonlinear effects in strongly-coupled SiN integrated waveguides [197], [198]. In these demonstrations, the authors exploited the significantly different dispersion profiles of the transverse supermodes in a strongly-coupled dual-core SiN waveguide to achieve simultaneous dual-supermode NIR SC generation by pumping with ultrashort optical pulses at 1.5 μm [197]. Arrays of more than two coupled waveguides have also been considered for dispersion engineering and broadband SC generation. For example, Fatema et al. exploited multiple mode couplings in a thin SiN array of five waveguides to achieve a dispersion profile that was restricted in value within 90 ps/nm/km in the wavelength range from 1,350 to 1,900 nm, enabling the numerical demonstration of octave-spanning SC generation [199]. An experimental demonstration of SC generation in the MIR spectral region for spectroscopy applications was reported using a SiN dual-core waveguide, whose careful design achieved a flatter dispersion profile in comparison to that typically observed in conventional single-core waveguides [200]. Using these waveguides, ultra-broadband MIR SC generation in the range 2,000–3,700 nm was experimentally demonstrated by pumping the dual-core waveguide at 1,550 nm. Besides SC generation, coupled nonlinear waveguides have also been extensively utilized for other applications, such as modulation instability of discrete solitons [201], soliton-based optical switching [202] and parametric amplification [203], [204], to name a few; however, these applications will not be covered in this review.

**Figure 9:**
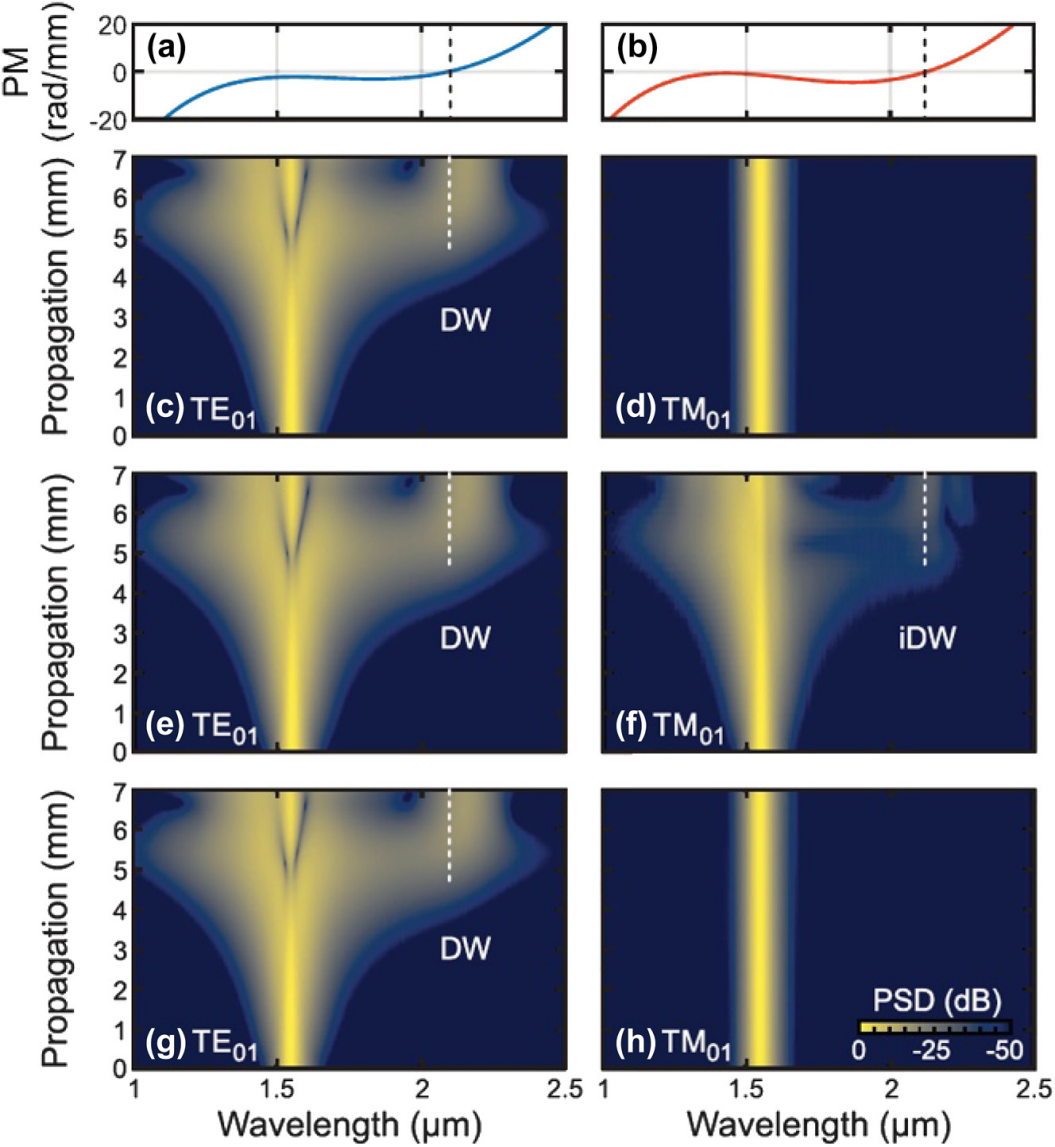
Comparison of single-mode and multimode numerical simulations carried out by Lüpken et al. [206] to demonstrate the effect of intermodal DW generation. Phase mismatch (PM) versus wavelength for (a) the single-mode and (b) the multimode case; single-mode numerical simulations of the normalized spectral dynamic of (c) the high-energy TE_01_ mode and (d) the low-energy TM_01_ mode; spectral dynamics of (e) the high-energy TE_01_ mode and (f) low-energy TM_01_ mode resulting from a numerical simulation where both modes were excited simultaneously; (g) and (h) report numerical simulations in the same conditions as in (e) and (f), but with disabled iXPM. The dashed lines indicate the phase-matched wavelengths. PSD: power spectral density. This figure is reproduced from ref. [206] under a CC BY 4.0 license.

Most of the studies reported in the literature focused on the generation of broadband SC thanks to the contribution of several excited modes based on the optimisation of intramodal nonlinear phenomena. Nevertheless, intermodal nonlinear phenomena can favour the generation of DWs as it has been widely demonstrated in optical fibers by exploiting the FWM process to transfer energy between different modal distributions [205]. Lüpken et al. reported an interesting intermodal mechanism relying on DW generation induced by iXPM between different transverse modes in a SiN waveguide [206]. In order to analyze the effect of intermodal DW generation, the authors considered two different transverse modes excited simultaneously, of which only one mode (TE_01_) had sufficient energy to form higher-order solitons and generate DWs during fission, while the other mode (TM_01_) had insufficient energy to generate a DW. The new mechanism of intermodal DW generation, which was demonstrated both numerically and experimentally, can be easily explained by considering Figure 9, which shows the comparison of single-mode and multimode numerical simulations, with the calculated phase mismatch as a function of wavelength for the single-mode and multimode case in panel (a) and (b), respectively [206]. First, the two modes were excited separately with energies of 170 and 20 pJ for the TE_01_ and the TM_01_ mode, respectively. The single-mode numerical simulation of the normalized spectral response of the strong TE_01_ mode is shown in Figure 9(c) where, as expected, the input pulse initially undergoes SPM followed by soliton fission and generation of a phase-matched DW at ≈2.2 μm, in good agreement with the phase mismatch calculation reported in Figure 9(a). Meanwhile, the single-mode simulation of the normalized spectral response of the TM_01_ mode shown in Figure 9(d) shows that this weak mode does not have enough energy to induce soliton formation and spectral broadening. When the two modes propagate at the same time through the waveguide (see Figure 9(e) and (f) for the spectral evolution of the strong TE_01_ and the weak TM_01_ mode, respectively), the TE_01_ mode experiences the same dynamics described before, while a relevant spectral broadening of the weak TM_01_ mode is induced by the iXPM effect. Thereby, the spectrum of the weak mode becomes sufficiently broad to reach the spectral region where the phase-matching condition is satisfied thus causing the formation and growth of an intermodal DW (iDW) radiation. The authors confirmed the origin of such an effect by performing a third simulation where the two modes propagated simultaneously, but the iXPM was not included in the numerical algorithm. This case is shown in Figure 9(g) and (h) for the TE_01_ and TM_01_ modes, respectively, confirming that the TM_01_ mode does not experience any spectral broadening.

SC generation by soliton fission has proved an extremely powerful and flexible technique. Despite this, the level of coherence of the radiation can be limited by the modulation instability effect, especially in the presence of pump pulses that carry high phase noise [207]. Such an issue can be tackled by following a completely different approach based on the exploitation of nonlinear media exhibiting normal dispersion in the wavelength range of interest, thus suppressing the role of modulation instability. In such a case, SC generation is primarily due to SPM and optical wave breaking [208]. Cheng et al. extended this concept to the multimodal case, by exploiting a chalcogenide (As_2_Se_3_) multimode strip waveguide, which represents a promising material for MIR SC generation due to its transparency window in the wavelength range from 0.85 to 17.5 μm [209]. The waveguide structure was properly optimized to guarantee that the pump pulses travel in the normal dispersion regions of the three employed optical modes TE_00_, TE_10_ and TE_20_. Through numerical simulations, the authors investigated the best conditions in terms of the wavelengths of the pump pulses launched in the normal dispersion regions, peak powers and pulse durations. Highly coherent and octave-spanning MIR SC was generated by the three modes when an optimized pump pulse with 80 fs pulse duration, 3 kW peak power, and a 3 μm central wavelength was used.

To conclude, the use of integrated multimodal platforms opens up new perspectives for SC generation. The critical factors are represented by the spectral transparency range of the chosen nonlinear platform and the ability to engineer the dispersion of all the modes involved in the process over a wide band. High-performance and broadband building blocks for modal coupling and manipulation may represent a turning point in the development of integrated devices for this specific application.

## 5 Frequency combs and Raman lasing

Integrated optical sources are fundamental for advancing photonic technologies. They enable the incorporation of light sources directly onto integrated photonic circuits and facilitate the cost-effective production of compact and energy-efficient systems for advanced applications in telecommunications, data processing, sensing, imaging and quantum computing. Considerable research in this area has progressed the integration of linear sources such as distributed feedback lasers (DFBs) [210], vertical-cavity surface-emitting lasers (VCSELs) [211], light-emitting diodes (LEDs) [212], Fabry–Perot lasers [213], and quantum dot lasers [214]. However, new emerging applications have brought increasing attention to optical sources that exploit nonlinear effects, such as OPOs [215], [216], optical frequency combs [217] and Raman lasers [218] because they can generate coherent and ultra-broadband light in a wide range of wavelengths without relying on the material properties of an active medium.

Integrated micro-resonators realised in a variety of materials and geometries, including whispering gallery mode (WGM) resonators, micro-rings, micro-disks, and photonic crystals, have been essential for the development of nonlinear optical sources. Although historically they have been used in optical filters and modulators, these compact optical cavities with a high-quality-factor (*Q*) can confine light within a small volume for extended periods, significantly intensifying light–matter interactions. By doing so, they can enhance the nonlinear effects and reduce the power requirements of nonlinear optical sources to enable the generation of new frequencies. When paired with Kerr nonlinearity, micro-resonators can be used to efficiently generate frequency combs with equally spaced wavelengths [217]. They can also be used to increase the efficiency of the nonlinear Raman scattering process exploited in Raman lasers to generate photons at a different wavelength with low pump powers [218].

In recent years, micro-resonators based on multimode waveguides have also gained interest. Unlike their single-mode counterpart, they allow for complex interactions between different modes that can be exploited to achieve phase-matching in nonlinear processes. They provide larger effective mode areas that support higher output powers and offer an advantage in achieving the ultra-low losses desirable in nonlinear applications since the optical modes supported by the waveguide have a lower overlap with their sidewalls, thus potentially reducing scattering losses without requiring complex fabrication schemes.

In this section, we discuss how resonators based on multimode waveguides have been used to explore new frequency generation schemes, demonstrate frequency combs with broadband operation and record-low threshold power (both in single-mode and multimode operation), and realise widely-tunable Raman lasers. It is important to note that many other types of microresonators, such as those utilizing whispering gallery modes, are not considered within the scope of this review. Our emphasis is strictly on the unique characteristics and applications of multimode waveguide microresonators, which differ significantly from other microresonator configurations. This distinction is crucial for understanding the specific behavior of the devices discussed here.

In the 2010s, researchers investigated multimode resonators as a way to generate multiple solitons within a single device to create frequency combs with improved stability and coherence for high-precision applications. Solitons are stable and localised pulses that travel around the resonator without changing shape, which can only be obtained when a balance between the waveguide dispersion and the nonlinear effects is achieved.

**Figure 10:**
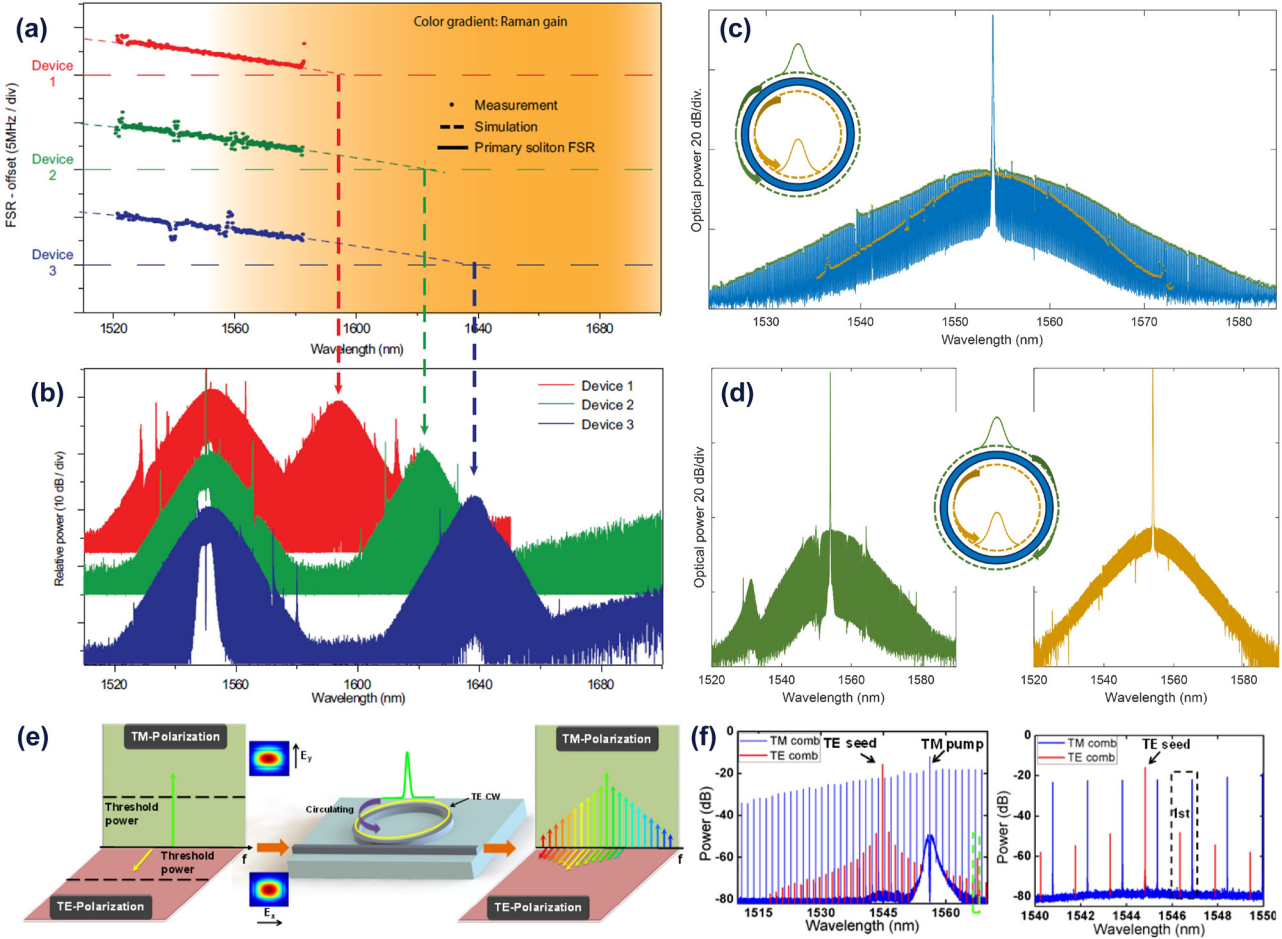
Frequency comb generation in multimode resonators. (a) FSR dispersion spectra for the Stokes-soliton forming mode family in three devices. The horizontal dashed lines give the repetition frequency of the primary soliton in each device [220]. (b) The measured primary and Stokes soliton spectra corresponding to the devices in (a) [220]. (c) Optical spectrum of the co-propagating dual-comb demonstrated in refs. [221], [222]. (d) Optical spectra of the two counter-propagating combs demonstrated in refs. [221], [222]. (e) Schematic of TE-polarized comb generation from a TM-polarized soliton comb [223]. (f) Optical spectrum of the TE- and TM-polarized combs (left) and zoom-in view (right) in the spectral range 1,540–1,550 nm [223]. (a)–(b) Panels are reproduced with permission from ref. [220] ©2017 Optica Publishing Group. (c)–(d) Panels are adapted from ref. [222] under a CC BY 4.0 license. (e)–(f) Panels are reproduced with permission from ref. [223] ©2019 Optica Publishing Group.

In 2017, Yang et al. demonstrated the Stokes soliton for the first time by utilizing the Raman amplification induced by a Kerr-generated soliton in a 3 mm silica WGM resonator with a *Q* factor of 250 million [219], [220]. This achievement was possible because the resonator supported an additional transverse mode family within the Raman gain spectrum that had a free spectral range (FSR) closely matching that of the Kerr soliton. When the resonator was pumped at 1,550 nm, generating a primary soliton with a frequency of 22 GHz, a second soliton emerged at 1,593 nm with the same frequency (see Figure 10(a) and (b)). This confirmed the optical trapping of the Stokes soliton by the Kerr soliton, as both of them exhibited synchronized repetition rates despite their differing absolute optical frequencies. This was the first observation of soliton trapping in multimode cavities.

A year later, Lucas et al. demonstrated a scalable method to simultaneously generate multiple dissipative Kerr soliton (DKS) based micro-combs in a crystalline MgF_2_ WGM cavity [221]. This multimode resonator supported 5 mode families that could sustain DKS formation with *Q* factors exceeding 10 × 10^9^. In this case, the simultaneous pumping of two resonances was achieved via the electro-optical modulation of a 1,554 nm laser with a single sideband and without carrier suppression. The modulation rate was selected to match the frequency separation of the two resonant modes. Since both mode families had distinct FSRs, the generated pulse streams had different repetition rates with an offset that was large enough to prevent soliton locking. The authors showed that the two mode families could be excited in co-propagating (see Figure 10(c)) or counter-propagating directions (see Figure 10(d)) with excellent coherence and that, by locking the laser to the resonator, the dual combs could be stably maintained for more than 12 h.

Bao et al. proved the generation of orthogonally polarized frequency combs as shown in Figure 10(e) via the XPM of two transverse mode families using a SiN micro-ring resonator with a 900 nm × 1,550 nm cross-section, a radius of 119 µm and *Q* factors of 1.3 × 10^6^ for TM and 7.8 × 10^5^ for TE polarisation [223]. To achieve this result, the micro-ring was first pumped with TM polarized light at 1,555.9 nm with a power of 600 mW to generate a TM frequency comb with a frequency of 191.8 GHz and a bandwidth of 5 THz. Then, a TE-polarised seed at a corresponding resonant wavelength (1,554.6 nm) was coupled to the ring with a power of 6.3 mW, which is much lower than the one required to generate a TE-polarised comb on its own. Nevertheless, a TE frequency comb was still produced by XPM induced by the strong TM comb when using both pumps (see Figure 10(f)).

Research on multimode resonators then moved to investigate the effect of higher-order dispersion to generate new frequency conversion schemes. In 2019, Kamel et al. showed clustered frequency comb formation by utilising an intermodal FWM process between two different transverse modes in a SiN micro-ring (cross-section = 720 nm × 1,400 nm, radius = 56 µm) with intrinsic *Q* = 4 × 10^5^ [224]. Since the resonator waveguides exhibited anomalous dispersion at telecom wavelengths, the device was initially used to generate a frequency comb by pumping it above its threshold power of 90 mW at 1,570 nm. Afterwards, the pump was slightly de-tuned to generate a sideband in a higher-order mode (TE_20_) at the second harmonic wavelength of 785 nm. Although SiN does not intrinsically possess second-order nonlinearity, it is believed that second harmonic generation was possible due to the effect of the interface between the core material and the silica cladding. The phase-matched FWM process between the pump and its second harmonic then generated sidebands at 1,011 nm and 1,085 nm, which are wavelengths considerably far away from the pump. Finally, as the pump wavelength was tuned back into resonance, a clustered comb appeared near 1,000 nm, since one of the sidebands acted as a pump to generate the new comb.

Until the time of that research, most works had focused on generating frequency combs using waveguides operating in the anomalous dispersion regime because normal dispersion would inhibit the modulation instability required to initiate a frequency comb. However, the need to further improve the propagation losses of the fully integrated resonators in order to increase their *Q* factors and reduce the threshold powers associated with optical frequency combs, led to the investigation of comb generation in much bigger waveguides operating in the normal dispersion regime. In this regime, the mode coupling, which is typically detrimental to comb generation, can be exploited to create hybrid modes that avoid modal crossings and break the normal dispersion. Furthermore, dual pump configurations can also take advantage of XPM to induce the modulation instability required for comb generation.

Zhang et al. proposed a silicon multimode racetrack resonator driven by two pump lasers to achieve frequency comb generation in the normal dispersion regime exploiting mode coupling and XPM [226], [227]. The racetrack resonator (bend radius = 130 µm, length of the straight waveguide of the racetrack = 1,000 µm) was based on a silicon rib waveguide with a height of 220 nm, etch depth of 70 nm and width of 2 µm with losses as low as 0.41 dB/cm that enabled a high *Q* = 1.8 × 10^6^. This waveguide operated in the normal dispersion regime at wavelengths between 1,400 and 1,700 nm. However, when two pumps in the mode coupling regime were launched simultaneously, the presence of the higher-order mode resonance affected the dispersion of the fundamental mode enabling the generation of a highly efficient frequency comb with 11 tones. In order to significantly decrease the pump power required to observe the frequency combs, the authors applied a reverse bias to a p-i-n diode fabricated near the resonators to remove the free carriers produced by TPA. When the applied reverse bias was 25 V, the power required for the pumps was reduced to a sub-milliwatt value of 0.3 mW with a conversion efficiency of −24.4 dB.

**Figure 11:**
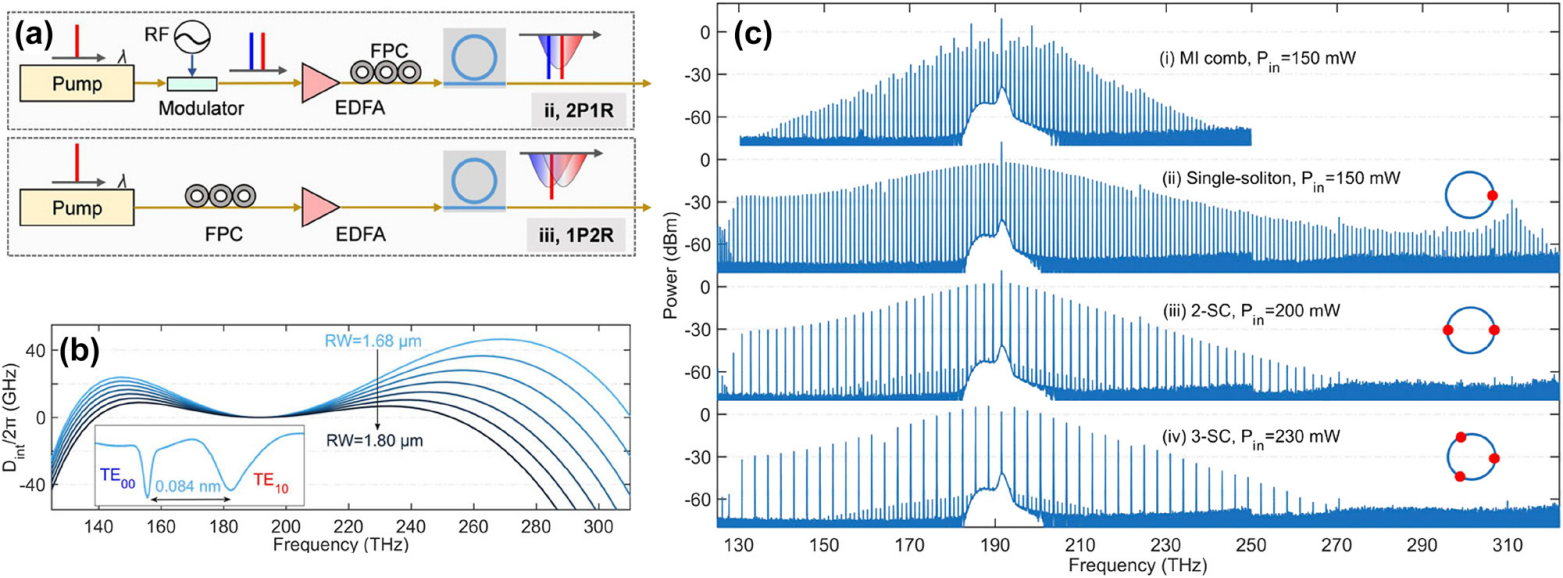
Octave-spanning DKS-based comb generation in multimode resonators. (a) Schematic of different thermal compensation schemes for Kerr soliton generation discussed in ref. [225]: (top panel) dual-pumping for one single resonance and (bottom panel) single pump to two close resonances (dual-mode scheme). RF: radio-frequency; FPC: fiber polarization controller; EDFA: Erbium-doped fiber amplifier. (b) Simulated integrated dispersion profiles with a zoomed-in view of the dual-mode. RW: ring width. (c) Optical spectra of (i) modulation instability (MI) comb, (ii) single-soliton, (iii) 2-soliton crystal (SC), and (iv) 3-soliton crystal states. All panels are adapted from ref. [225] under a CC BY 4.0 license.

In 2022, Weng et al. presented the scheme illustrated in Figure 11(a) to produce octave-spanning DKS-based combs by injecting a single pump to two close resonances in a SiN multimode racetrack resonator (cross-section = 800 nm × 1,680 nm, radius = 23.3 µm) with *Q* = 1.1 × 10^6^ for the TE_00_ mode and *Q* = 4.2 × 10^5^ for the TE_10_ mode [225]. The width of the multimode waveguide was designed to ensure that the resonant wavelengths of the TE_00_ and TE_10_ modes were separated only by 0.084 nm (see Figure 11(b)) so that they both could be pumped with the same laser wavelength. Since the resonator exhibited the near-zero anomalous dispersion crucial for broadband micro-comb generation, it could be driven with a pump power of 150 mW in the dual-mode scheme to achieve the modulation instability micro-comb state with a spectral bandwidth of 136–240 THz (Figure 11(c)(i)) and the single-soliton state with a wider bandwidth of 125–322 THz (Figure 11(c)(ii)). The authors also showed that with this configuration it was possible to achieve the soliton crystal state at slightly higher pump powers (200–230 mW) in the presence of avoided mode crossings (Figure 11(c)(iii) and (iv)). This state was characterised by having regularly distributed soliton pulses with stronger comb lines spaced by multiples of the cavity FSR, producing combs with spectral bandwidth between 127 and 270 THz, increased comb line power up to 31 mW, and enhanced conversion efficiencies of 13.5 %.

**Figure 12:**
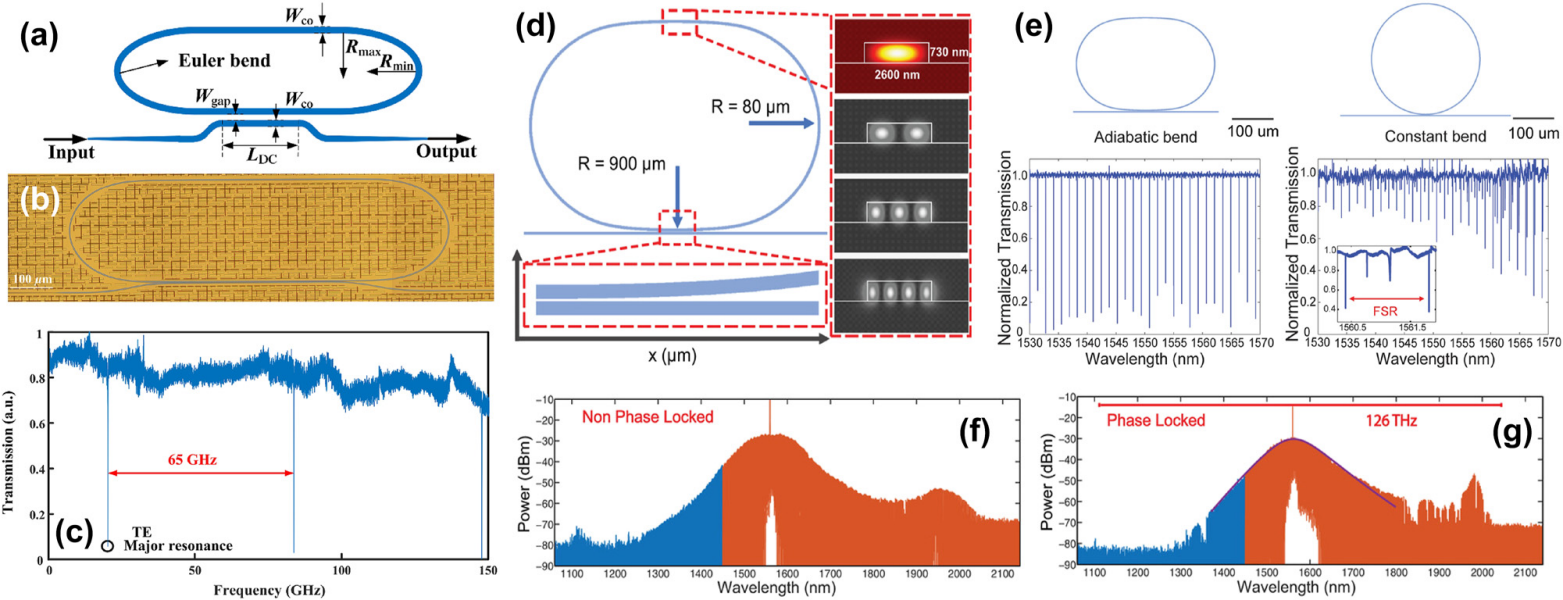
Ultra-high Q multimode resonators for comb generation. (a) Schematic diagram of the compact ultra-high *Q* multimode resonator based on adiabatic Euler bends reported in ref. [228]. (b) Fabricated ultra-high *Q* SiN racetrack resonator [228]. (c) Measured transmission spectrum for a multimode resonator with an ultra-high *Q* of 10.8 × 10^6^ and an FSR of 65 GHz [228]. (d) Schematic of the micro-resonator with adiabatic bends design reported in ref. [229]. (e) Measured transmission spectra with no higher-order modes observed (left) and with clear signatures of higher-order modes (right) [229]. (f) Broadband frequency comb before phase locking with an FSR of 174 GHz [229]. (g) A phase-locked single soliton frequency comb close to octave-spanning [229]. (a)–(c) Panels are reproduced from ref. [228] under a CC BY 4.0 license. (d)–(g) Panels are reproduced with permission from ref. [229] ©2020 Wiley-VCH GmbH.

Lately, research efforts have focused on optimising the geometry of multimode resonators to further increase their *Q* factors in order to achieve even lower threshold powers when operating solely in the fundamental mode. Cui et al. proposed a SiN racetrack resonator with adiabatic Euler bends, as illustrated in Figure 12(a) and (b) [228], characterized for having a curvature which varied linearly to reduce their footprint while ensuring the adiabatic transmission of the fundamental mode with low intermodal coupling. This resonator consisted of multimode waveguides (cross-section = 800 nm × 3 µm) with a maximum propagation loss of 3.3 dB/m. In this particular case, the radius of the bends ranged from a minimum value of 100 µm to a maximum of 4,000 µm with an overall effective radius of only 195 µm. Although the designed multimode waveguide supported higher-order modes for both TE and TM polarization, no multimode interference was observed due to the design of the bends and coupling regions which provided low cross-talk with higher-order modes. Thanks to the ultra-low losses of the multimode waveguides, the authors were able to demonstrate a resonator with a *Q* of 10.8 × 10^6^ and an FSR of 65 GHz that could be used to generate efficient optical frequency combs (see Figure 12(c)).

Similarly, Ji et al. proposed a micro-ring resonator with an optimised bend geometry designed to hinder the excitation of higher-order modes and prevent the increase of the power threshold required for nonlinear processes (Figure 12(d)) [229]. The optimised design was based on a highly multimode SiN waveguide (cross-section = 730 nm × 2,600 nm), which significantly reduced the losses due to sidewall roughness and provided a dispersion favourable for broadband comb generation. The bend region consisted of the same multimode waveguide with a bending radius that changed in a nonlinear manner, following a hyperbolic tangent function, from a value of 900 µm close to the coupling section to a value of 80 µm, which allowed for a small effective bending radius. Although the multimode waveguide supported 10 transverse modes, this bend geometry ensured a high degree of adiabaticity and effectively avoided the excitation of higher-order modes. Using this configuration, the authors were able to demonstrate a resonator with an ultra-high intrinsic *Q* of 31.8 × 10^6^ and ultra-low propagation losses of <1 dB/m. Compared to resonators with constant bend radius, the selected nonlinear adiabatic design ensured a transmission that only dipped at the selected FSR without traces of higher-order modes (see Figure 12(e)). This enabled the generation of a broadband, almost octave-spanning single-soliton frequency comb with an FSR of 174 GHz covering the range from 1,097 to 2,040 nm, equivalent to a spectral bandwidth of 126 THz, when phase-locking the resonator using the micro-heater thermal tuning method (Figure 12(f) and (g)). Furthermore, the design reduced the pump power threshold to 73 µW for parametric oscillation for a pump wavelength of around 1,560 nm.

More recently, Ye et al. demonstrated a robust tapered bus waveguide design on an AlGaAs on insulator (AlGaAsOI) platform to achieve selective-mode coupling in resonators and increase their *Q* factor without altering the shape of the resonances [231]. The resonator consisted of a ring (cross-section = 360 nm × 780 nm, radius = 25 µm) which supported both the TE_00_ and TE_10_ modes. This ring was paired with a mode-selective bend tapered bus waveguide, whose width was linearly tapered from *W*_1_ (*W*_1_ = *W*_0_ + 50 nm), with *W*_0_ being the central waveguide width) to *W*_2_ (*W*_2_ = *W*_0_ − 50 nm), to effectively filter out undesired higher-order modes even with fabrication variations of up to 30 nm that enabled achieving *Q* factors as high as 4.4 × 10^5^. The authors also proposed using the same type of resonator with wider waveguides (cross-section = 360 nm × 1,000 nm, radius = 50 µm) operating in the normal dispersion regime to generate dark-pulse combs. This type of comb exhibits better thermal stability, as it is generated by tuning the blue side of the resonance, whose range can be extended via the thermal nonlinear effects. When pumping the resonator at 1,580.23 nm, it exhibited a *Q* = 7.8 × 10^5^ and an FSR = 265 GHz that allowed demonstrating dark-pulse combs with an initial existence range of 77 GHz through forward tuning that could be extended to 89 GHz with additional backward tuning operation.

**Figure 13:**
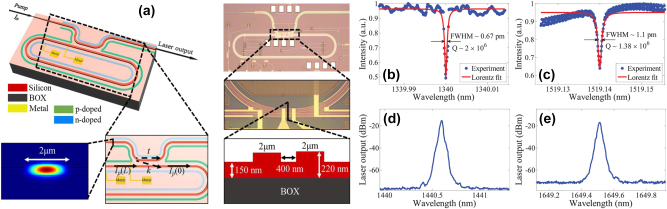
Raman laser based on a multimode racetrack resonator. (a) Configuration of the silicon racetrack resonator reported in ref. [230] and modal characteristics. Transmission characteristics of the resonator around resonances of (b) 1,340 nm and (c) 1,519 nm. Measured Raman laser spectra at pump wavelengths of (d) 1,340 nm and (e) 1,519 nm with a reverse bias voltage of 25 V. All panels are reproduced with permission from ref. [230] ©2020 Wiley-VCH GmbH.

Besides optical frequency comb generation, multimode resonators with high-*Q* factors have also been investigated in the context of integrated Raman lasers to provide higher output powers and reduce their pump threshold. These lasers exploit Raman scattering to achieve single-longitudinal mode operation with efficient wavelength conversion over a wavelength range well beyond the pump laser without requiring dispersion engineering. Initially, in ref. [230], Zhang et al. showed a Raman laser using a multimode racetrack resonator (radius = 130 µm, total length of the racetrack cavity = 2.8 mm) based on the silicon waveguide they previously used for optical frequency combs in ref. [227] (Figure 13(a)). For silicon, the Stokes frequency shift for Raman scattering is 15.6 THz. In this work, the coupling ratios for the pump and the Stokes wavelengths were designed below the required critical coupling ratios so that only a small portion of the power was cross-coupled into the racetrack section of the resonator. By doing so, most of the power generated at the Stokes wavelength was trapped in the cavity, enabling the build-up that eventually induced Raman lasing at a 15.6 THz de-tuning from the pump wavelength. When the lasing operation was tested with 1,340 and 1,519 nm pump wavelengths, Raman lasing was observed once the pump power exceeded the lasing threshold (Figure 13(b)–(e)). The laser powers were 27.9 μW at 1,440.6 nm and 19.4 μW at 1,649.5 nm. The lower output power observed at the longer wavelength was consistent with the smaller Raman gain coefficient and larger nonlinear losses exhibited by silicon at these wavelengths. By applying a bias of 25 V to a diode adjacent to the fabricated resonators, the authors were able to reduce the lasing thresholds down to 0.4 mW at 1,340 nm and 1.3 mW at 1,519 nm with slope efficiencies of 1.4 % and 1.1 %, respectively.

Later on, Zhang et al. improved the design of the racetrack resonator by including two Euler bends and a bent directional coupler to maintain high *Q* factors (2.2 × 10^6^) over a 200 nm wavelength range to demonstrate a step tunable Raman laser [232]. In this case, the Euler bends (maximum radius *R*_max_ = 2,000 µm, minimum radius *R*_min_ = 56 µm) were optimised to suppress the coupling of higher-order modes, while the bent directional coupler was engineered to provide a broadband coupling ratio that covered the 1,260–1,490 nm range. The resulting laser exhibited a tuning range of 157 nm between 1,320 and 1,480 nm, lasing threshold power of 0.2 mW and slope efficiency of 27.5 %, when injecting a pump wavelength between 1,240 and 1,380 nm with a 0.6 mW pump power and using a reverse bias of 25 V to remove the free carriers generated when the intra-cavity power was high. This approach provides a good higher-order mode suppression since only one main resonance was observed in the studied wavelength range.

As discussed in this section, the investigation of multimode waveguides within micro-resonators has enabled achieving high *Q* factors for the efficient generation of both frequency combs and tunable Raman lasers with low threshold powers. However, these devices still face challenges in terms of mode control and thermal management that must be addressed to fully harness their potential for the realization of stable and efficient nonlinear optical sources.

## 6 Conclusions

We have reviewed key research topics highlighting the growing potential of introducing spatial modes in nonlinear integrated circuits. Table 1 reports the advantages and limitations of nonlinear multimode photonics on-chip, mainly compared to its single-mode counterpart, future perspectives and research directions for the various applications discussed in this review. The introduction of intermodal effects in nonlinear systems offers significant advantages, such as enabling access to widely separated spectral regions for both classical and quantum applications. This approach also facilitates the development of widely tunable sources, even in spectral regions where such sources are scarce, thereby making a broader part of the optical spectrum accessible for different applications, and enables extreme spectral broadening by increasing the dimensionality over which phase matching can be achieved. Additionally, intramodal and intermodal effects in nonlinear multimode waveguides offer a compelling route to introducing parallelism in nonlinear optic schemes implementing logic functions, enhancing data processing rates without increasing the device footprint.

**Table 1:** Advantages, limitations and future research directions of nonlinear multimode photonics on-chip for different applications.

Application	Advantages	Limitations	Future perspectives
Telecommunications	– Broadband wavelength conversion between widely separated telecom bands – All-optical mode-selective wavelength conversion in MDM systems – Mitigation/suppression of undesired crosstalk in WDM systems	– Reduced overlap between the modes that yield a limited conversion efficiency – Difficult to realize long waveguides due to bend losses and mode cross-talk	– Low-loss, meter-long multimode waveguides with optimized bends for wavelength conversion – Low-loss, meter-long rare-earth-doped multimode waveguides amplifiers operating simultaneously on different spatial modes
Quantum	– Perfect phase matching at non-degenerate wavelengths – Increased spectral tunability tailored to the specific requirements – Facilitates separation between signal and idler, and pump filtering	– Reduced overlap between the modes that yield a limited generation efficiency – Waveguide tailoring to accommodate the required high order modes	– Squeezed state manipulation and quantum noise reduction for quantum measurements – Spatial mode engineering to enhance applications in quantum computing and communication
MIR generation	– Broad MIR generation tunability exploiting the degree of freedom given by multimodality – Realization of narrow-linewidth MIR sources ideal for spectroscopy using well-established NIR pumps	– Reduced overlap between the modes that yield a limited conversion efficiency – Difficult to realize long waveguides due to bend losses and mode crosstalk	– Low-loss, meter-long multimode waveguides with optimized bends for efficient MIR generation – High-efficiency intermodal-DFG-based MIR generation in poling-free TFLN waveguides
All-optical signal processing	– Parallel signal processing on different channels through MDM within a single multimode waveguide with diverse functionalities – Possibility to exploit intermodal nonlinear effects to perform flexible all-optical control, for example, in all-optical switching	– Typically lower nonlinear efficiency for higher-order modes due to large effective areas – Modal crosstalk can degrade signal quality – Spatial mode handling demands complex and fabrication-tolerant (de)multiplexers	– Increase the number of processed channels utilizing nonlinear multimode waveguides supporting several spatial modes – Design fully-integrated devices for all-optical switching to avoid challenges in free-space mode excitation and coupling
SC generation	– Dispersion engineering of various modes results in multiple phase matching conditions, providing an additional tool for SC shaping and bandwidth enhancement – Larger waveguide cross-section reduces risks of damage when high peak power pulses are used	– Critical control over simultaneous mode excitation is needed – Critical dispersion control is needed to guarantee a proper soliton propagation condition in each mode – Power exchange between different modes can induce additional noise in the SC radiation	– Spatio-temporal coupling of intermodal nonlinear effects is at an early stage of study and could provide additional tools for SC shaping and bandwidth enhancement – Optimization of simultaneous coupling of the desired modes to reduce intermodal beating
Frequency combs and Raman lasing in multimode waveguide microresonators	– Multimode microresonators can achieve very high *Q* factors with a small footprint, significantly reducing power requirements – Higher-order modes can be exploited to achieve tailored dispersion profiles in multimode microresonators	– Unwanted mode coupling and crosstalk can occur, complicating the control of the desired nonlinear processes – Low propagation and bending losses are required, particularly when higher-order modes are considered	– Combining microresonators with other on-chip components to enhance performance – Advanced waveguide bend designs to reduce mode coupling and intermodal crosstalk – Thermal management strategies to mitigate thermal effects and enhance stability

Beyond intermodal interactions, we have also examined applications that have used multimode waveguides to benefit from the low loss propagation offered by the larger dimension structures, but which have suppressed higher-order modes. This strategy has proved effective in generating single-mode frequency combs and Raman lasers.

Material selection remains a critical challenge in realising these nonlinear devices, with various options offering distinct advantages. The variety of materials involved in the applications we have reviewed underscores the ongoing search for optimal solutions. Silicon nitride is particularly attractive for its extremely broadband performance and resistance to free-carrier effects at the more conventional near-infrared wavelengths; this is in contrast to silicon, which is limited by these effects. However, other nonlinear materials, such as tantalum pentoxide and organic polymers on silicon, have also gained interest.

Nonlinear multimode photonics could also significantly benefit from the implementation of topological photonic structures, which are emerging as a powerful tool to enable robust light transport in integrated platforms. The use of topological phases in photonic crystals with high Chern numbers allows the simultaneous propagation of multiple unidirectional modes immune to backscattering, even in the presence of substantial fabrication disorder. In this context, Skirlo et al. showed that multimode one-way edge states could be engineered in two-dimensional photonic crystals, with each mode associated with a distinct topological invariant [233]. These results suggest the possibility of designing multimode waveguides where intermodal interactions can be enhanced or selectively suppressed depending on the topological character of the supported modes. More recently, the feasibility of integrating topological protection into silicon photonics platforms has been demonstrated in ref. [234], where topological and trivial edge states were realized and distinguished in a one-dimensional array of coupled waveguides. The ability to reconfigure the topological properties through local perturbations opens new routes for dynamic control of modal dispersion, which can be beneficial in broadband nonlinear processes such as intermodal FWM. Finally, in ref. [235], the generation and transport of biphoton states in topologically protected waveguides were experimentally demonstrated using a one-dimensional array of coupled waveguides. The preservation of quantum correlations despite structural disorder highlights the potential of these systems for quantum nonlinear photonics. These approaches are highly complementary to the use of intermodal nonlinearities in engineered multimode waveguides. The integration of topological design principles could offer additional resilience to modal phase-matching conditions and suppress undesired back-conversion or crosstalk in parametric processes. As topological photonics matures towards scalable integration, its intersection with nonlinear multimode platforms is likely to yield novel functionalities, both in classical and quantum regimes.

To realise the full potential of intermodal nonlinearities in integrated devices, additional on-chip functionalities will be essential. In this respect, we have reviewed demonstrations incorporating components, such as mode converters and splitters, paving the way for more sophisticated multimode nonlinear systems. Future developments will integrate active and passive elements – such as pump sources for the nonlinear processes of interest, electro-optic modulators, sensors and detectors – gradually enabling complete optical systems that have traditionally relied on bulk components to be realised within an integrated platform.

A natural direction for future work is the extension of current approaches to systems supporting a greater number of spatial modes. Most demonstrations to date involve only two or three modes, mainly due to the increased complexity of achieving efficient intermodal phase matching and strong nonlinear overlap as the modal order grows. Higher-order modes are also more susceptible to losses and fabrication variations. However, increasing the number of modes would directly scale several of the concepts highlighted in this review. More mode pairs mean more independent phase-matching channels, enabling broader and more flexible spectral translation, including the possibility to concatenate multiple converted bands across a wide range, with the only limitation being the material transparency. At the same time, larger waveguides can handle higher optical powers and support stronger nonlinear interactions without compromising integration. Access to a richer set of spatial profiles would also allow for finer spatial shaping and routing of light, or the design of more complex switching and logic gate schemes. These capabilities could be particularly valuable in applications that require high throughput, broad spectral access, or multidimensional control. The full exploitation of this high-dimensional space, both theoretically and technologically, remains a largely open and promising challenge for multimode nonlinear integrated photonics.

## References

[1] Stolen R. H. (2008). The early years of fiber nonlinear optics. *J. Lightwave Technol.*.

[2] Agrawal G. P. (2019). *Nonlinear Fiber Optics*.

[3] Agrawal G. P. (2020). *Applications of Nonlinear Fiber Optics*.

[4] Brès C.-S., Della Torre A., Grassani D., Brasch V., Grillet C., Monat C. (2023). Supercontinuum in integrated photonics: generation, applications, challenges, and perspectives. *Nanophotonics*.

[5] Pu M. (2018). Ultra-Efficient and broadband nonlinear AlGaAs-on-insulator chip for low-power optical signal processing. *Laser Photonics Rev.*.

[6] Hendrickson S. M., Foster A. C., Camacho R. M., Clader B. D. (2014). Integrated nonlinear photonics: emerging applications and ongoing challenges. *JOSA B*.

[7] Lacava C., Ettabib M. A., Petropoulos P. (2017). Nonlinear silicon photonic signal processing devices for future optical networks. *Appl. Sci.*.

[8] Baboux F., Moody G., Ducci S. (2023). Nonlinear integrated quantum photonics with AlGaAs. *Optica*.

[9] Piergentili P. (2023). Quantum information with integrated photonics. *Appl. Sci.*.

[10] Dutt A., Mohanty A., Gaeta A. L., Lipson M. (2024). Nonlinear and quantum photonics using integrated optical materials. *Nat. Rev. Mater.*.

[11] Scalari G., Faist J., Picqué N. (2019). On-chip mid-infrared and THz frequency combs for spectroscopy. *Appl. Phys. Lett.*.

[12] Hwang A. Y. (2023). Mid-infrared spectroscopy with a broadly tunable thin-film lithium niobate optical parametric oscillator. *Optica*.

[13] Wright L. G., Renninger W. H., Christodoulides D. N., Wise F. W. (2022). Nonlinear multimode photonics: nonlinear optics with many degrees of freedom. *Optica*.

[14] Cristiani I. (2022). Roadmap on multimode photonics. *J. Opt.*.

[15] Li C., Liu D., Dai D. (2019). Multimode silicon photonics. *Nanophotonics*.

[16] Dai D. (2018). Advanced passive silicon photonic devices with asymmetric waveguide structures. *Proc. IEEE*.

[17] Su Y., He Y., Chen H., Li X., Li G. (2021). Perspective on mode-division multiplexing. *Appl. Phys. Lett.*.

[18] Du J., Shen W., Liu J., Chen Y., Chen X., He Z. (2021). Mode division multiplexing: from photonic integration to optical fiber transmission. *Chin. Opt. Lett.*.

[19] Liu R., Lu L., Zhang P., Chang W., Liu D., Zhang M. (2020). Integrated dual-mode 3-dB power splitter based on multimode interference coupler. *IEEE Photonics Technol. Lett.*.

[20] Ye C., Zhang M., Shi Y., Dai D. (2020). Broadband dual-mode 2 × 2 3 dB multimode interference couplers with a shallowly etched multimode section. *Appl. Opt.*.

[21] Pérez-Armenta C. (2022). Polarization-independent multimode interference coupler with anisotropy-engineered bricked metamaterial. *Photonics Res.*.

[22] González-Andrade D. (2020). Experimental demonstration of a broadband mode converter and multiplexer based on subwavelength grating waveguides. *Opt. Laser. Technol.*.

[23] Chack D., Hassan S., Qasim M. (2020). Broadband and low crosstalk silicon on-chip mode converter and demultiplexer for mode division multiplexing. *Appl. Opt.*.

[24] Wang Q. (2022). On-chip mode division (de) multiplexer for multi-band operation. *Opt. Express*.

[25] Haines J. (2024). Subwavelength and broadband on-chip mode splitting with shifted junctions. *Opt. Express*.

[26] Haines J. (2024). Fabrication of 1 × N integrated power splitters with arbitrary power ratio for single and multimode photonics. *Nanophotonics*.

[27] Huang Q. (2024). Co-planar arbitrary ratio optical power splitter based on cascaded hybrid-core vertical directional couplers for arbitrary guide modes. *Opt. Laser. Technol.*.

[28] Li S. (2018). Universal multimode waveguide crossing based on transformation optics. *Optica*.

[29] Wu B., Yu Y., Zhang X. (2020). Multimode waveguide crossing with ultralow loss and low imbalance. *Opt. Express*.

[30] Wu H., Li C., Song L., Tsang H.-K., Bowers J. E., Dai D. (2019). Ultra-sharp multimode waveguide bends with subwavelength gratings. *Laser Photonics Rev.*.

[31] Sun S., Dong P., Zhang F., Wang J., Zhu N., Shi Y. (2021). Inverse design of ultra-compact multimode waveguide bends based on the free-form curves. *Laser Photonics Rev.*.

[32] Kittlaus E. A., Otterstrom N. T., Rakich P. T. (2017). On-chip inter-modal Brillouin scattering. *Nat. Commun.*.

[33] Liu Y. (2021). Circulator-free Brillouin photonic planar circuit. *Laser Photonics Rev.*.

[34] Aryanfar I., Wolff C., Steel M., Eggleton B. J., Poulton C. G. (2014). Mode conversion using stimulated Brillouin scattering in nanophotonic silicon waveguides. *Opt. Express*.

[35] Ji K., Davidson I., Sahu J., Richardson D. J., Wabnitz S., Guasoni M. (2023). Mode attraction, rejection and control in nonlinear multimode optics. *Nat. Commun.*.

[36] Ji K., Richardson D. J., Wabnitz S., Guasoni M. (2024). Sub-nanosecond all-optically reconfigurable photonics in optical fibres. ..

[37] Guasoni M. (2015). Generalized modulational instability in multimode fibers: wideband multimode parametric amplification. *Phys. Rev. A*.

[38] Wu F. O., Hassan A. U., Christodoulides D. N. (2019). Thermodynamic theory of highly multimoded nonlinear optical systems. *Nat. Photonics*.

[39] Krupa K. (2017). Spatial beam self-cleaning in multimode fibres. *Nat. Photonics*.

[40] Chang L. (2020). Second order nonlinear photonic integrated platforms for optical signal processing. *IEEE J. Sel. Top. Quantum Electron.*.

[41] Leuthold J., Koos C., Freude W. (2010). Nonlinear silicon photonics. *Nat. Photonics*.

[42] Ding Y., Xu J., Ou H., Peucheret C. (2014). Mode-selective wavelength conversion based on four-wave mixing in a multimode silicon waveguide. *Opt. Express*.

[43] Xiong J., Yu Y., Yang W., Sun C., Zhang X. (2019). Crosstalk suppressed high efficient mode-selective four-wave mixing through tailoring waveguide geometry. *IEEE Photonics J.*.

[44] Qiu Y. (2017). Mode-selective wavelength conversion of OFDM-QPSK signals in a multimode silicon waveguide. *Opt. Express*.

[45] Renaudier J. (2022). Devices and fibers for ultrawideband optical communications. *Proc. IEEE*.

[46] Ferrari A. (2020). Assessment on the achievable throughput of multi-band ITU-T G.652.D fiber transmission systems. *J. Lightwave Technol.*.

[47] Escobar-Landero S., Lorences-Riesgo A., Zhao X., Frignac Y., Charlet G. (2023). Ultra-wideband high-capacity transmission systems: challenges and opportunities. *49th European Conference on Optical Communications (ECOC 2023)*.

[48] Hazarika P. (2024). Multi-band transmission over E-S-C- and L-band with a hybrid Raman amplifier. *J. Lightwave Technol.*.

[49] Shimizu S. (2024). L- and U-band WDM transmission over 6 THz using PPLN-based optical parametric amplification and wavelength-band conversion. *J. Lightwave Technol.*.

[50] Yoo S. B. (1996). Wavelength conversion technologies for WDM network applications. *J. Lightwave Technol.*.

[51] Kachris C., Tomkos I. (2012). A survey on optical interconnects for data centers. *IEEE Commun. Surv. Tutorials*.

[52] Minami H. (2024). Low-penalty band-switchable multi-band optical cross-connect using PPLN-based inter-band wavelength converters. *J. Lightwave Technol.*.

[53] Foster M. A., Turner A. C., Salem R., Lipson M., Gaeta A. L. (2007). Broad-band continuous-wave parametric wavelength conversion in silicon nanowaveguides. *Opt. Express*.

[54] Ophir N. (2010). Continuous wavelength conversion of 40-Gb/s data over 100 nm using a dispersion-engineered silicon waveguide. *IEEE Photonics Technol. Lett.*.

[55] Ophir N. (2012). Wavelength conversion and unicast of 10-Gb/s data spanning up to 700 nm using a silicon nanowaveguide. *Opt. Express*.

[56] Ayan A., Mazeas F., Liu J., Kippenberg T. J., Brès C.-S. (2022). Polarization selective ultra-broadband wavelength conversion in silicon nitride waveguides. *Opt. Express*.

[57] Ayan A., Liu J., Kippenberg T. J., Brès C.-S. (2023). Towards efficient broadband parametric conversion in ultra-long Si_3_N_4_ waveguides. *Opt. Express*.

[58] Signorini S. (2018). Intermodal four-wave mixing in silicon waveguides. *Photonics Res.*.

[59] McKinstrie C. J., Radic S., Chraplyvy A. R. (2002). Parametric amplifiers driven by two pump waves. *IEEE J. Sel. Top. Quantum Electron.*.

[60] Friis S. M. M. (2016). Inter-modal four-wave mixing study in a two-mode fiber. *Opt. Express*.

[61] Vitali V. (2024). Fully integrated and broadband Si-rich silicon nitride wavelength converter based on Bragg scattering intermodal four-wave mixing. *Photonics Res.*.

[62] Lacava C. (2019). Intermodal Bragg-scattering four wave mixing in silicon waveguides. *J. Lightwave Technol.*.

[63] Signorini S., Finazzer M., Bernard M., Ghulinyan M., Pucker G., Pavesi L. (2019). Silicon photonics chip for inter-modal four wave mixing on a broad wavelength range. *Front. Phys.*.

[64] Ronniger G., Lischke S., Mai C., Zimmermann L., Petermann K. (2020). Investigation of inter-modal four wave mixing in p-i-n diode assisted SOI waveguides. *2020 IEEE Photonics Society Summer Topicals Meeting Series (SUM)*.

[65] Ronniger G. (2021). Efficient ultra-broadband C-to-O band converter based on multi-mode silicon-on-insulator waveguides. *2021 European Conference on Optical Communication (ECOC)*.

[66] Kernetzky T., Ronniger G., Höfler U., Zimmermann L., Hanik N. (2021). Numerical optimization and CW measurements of SOI waveguides for ultra-broadband C-to-O-band conversion. *2021 European Conference on Optical Communication (ECOC)*.

[67] Gajda A. (2012). Highly efficient CW parametric conversion at 1550 nm in SOI waveguides by reverse biased pin junction. *Opt. Express*.

[68] Vitali V. (2021). Design rules for low electrical power consumption in nonlinear silicon waveguides with active carrier removal. *CLEO: QELS_Fundamental Science*.

[69] McKinstrie C., Harvey J., Radic S., Raymer M. (2005). Translation of quantum states by four-wave mixing in fibers. *Opt. Express*.

[70] Bell B. A., Xiong C., Marpaung D., McKinstrie C. J., Eggleton B. J. (2017). Uni-directional wavelength conversion in silicon using four-wave mixing driven by cross-polarized pumps. *Opt. Lett.*.

[71] Lacava C. (2019). Intermodal frequency generation in silicon-rich silicon nitride waveguides. *Photonics Res.*.

[72] Gardes F. (2022). A review of capabilities and scope for hybrid integration offered by silicon-nitride-based photonic integrated circuits. *Sensors*.

[73] Vitali V. (2024). L- to U-band wavelength conversion of QPSK signals using intermodal four-wave mixing. *IEEE Photonics Technol. Lett.*.

[74] Gow P. C. (2024). Mechanical dicing of optical quality facets and waveguides in a silicon nitride platform. *Electron. Lett.*.

[75] Riemensberger J., Kuznetsov N., Liu J., He J., Wang R. N., Kippenberg T. J. (2022). A photonic integrated continuous-travelling-wave parametric amplifier. *Nature*.

[76] Ye Z., Zhao P., Twayana K., Karlsson M., Torres-Company V., Andrekson P. A. (2021). Overcoming the quantum limit of optical amplification in monolithic waveguides. *Sci. Adv.*.

[77] Li S. (2020). Compact and broadband multimode waveguide bend by shape-optimizing with transformation optics. *Photonics Res.*.

[78] Xu B. (2025). High performance B-spline multimode waveguide bends in lithium niobate on insulator. *Opt. Express*.

[79] Sun S. (2022). Ultra-sharp silicon multimode waveguide bends based on double free-form curves. *Photonics Res.*.

[80] Liu Y. (2022). A photonic integrated circuit–based erbium-doped amplifier. *Science*.

[81] Wang J., Sciarrino F., Laing A., Thompson M. G. (2020). Integrated photonic quantum technologies. *Nat. Photonics*.

[82] Alexander K. (2024). A manufacturable platform for photonic quantum computing. ..

[83] Somaschi N. (2016). Near-optimal single-photon sources in the solid state. *Nat. Photonics*.

[84] Esmann M., Wein S. C., Antón-Solanas C. (2024). Solid-state single-photon sources: recent advances for novel quantum materials. *Adv. Funct. Mater.*.

[85] Zhang J., Chattaraj S., Huang Q., Jordao L., Lu S., Madhukar A. (2022). On-chip scalable highly pure and indistinguishable single-photon sources in ordered arrays: path to quantum optical circuits. *Sci. Adv.*.

[86] Signorini S., Pavesi L. (2020). On-chip heralded single photon sources. *AVS Quantum Sci.*.

[87] Feng L. (2022). Silicon photonic devices for scalable quantum information applications. *Photonics Res.*.

[88] Lee J.-M. (2023). Do different kinds of photon-pair sources have the same indistinguishability in quantum silicon photonics?. *Photonics Res.*.

[89] Meyer-Scott E., Silberhorn C., Migdall A. (2020). Single-photon sources: approaching the ideal through multiplexing. *Rev. Sci. Instrum.*.

[90] Karpinski M., Radzewicz C., Banaszek K. (2012). Generation of spatially pure photon pairs in a multimode nonlinear waveguide using intermodal dispersion. *Quantum Communications and Quantum Imaging X*.

[91] Signorini S. (2021). A silicon source of heralded single photons at 2 μm. *APL Photonics*.

[92] Sanna M., Rizzotti D., Signorini S., Pavesi L. (2024). 2 μm ghost spectroscopy with an integrated silicon quantum photonics source. *Adv. Quantum Technol.*.

[93] Paesani S., Borghi M., Signorini S., Maïnos A., Pavesi L., Laing A. (2020). Near-ideal spontaneous photon sources in silicon quantum photonics. *Nat. Commun.*.

[94] Feng L.-T. (2019). On-chip transverse-mode entangled photon pair source. *npj Quantum Inf.*.

[95] Deng F.-G., Ren B.-C., Li X.-H. (2017). Quantum hyperentanglement and its applications in quantum information processing. *Sci. Bull.*.

[96] Kim Y. (2023). Evidence for the utility of quantum computing before fault tolerance. *Nature*.

[97] Tindall J., Fishman M., Stoudenmire E. M., Sels D. (2024). Efficient tensor network simulation of IBM’s eagle kicked Ising experiment. *PRX Quantum*.

[98] Borghi M., Pavesi L. (2022). Mitigating indistinguishability issues in photon pair sources by delayed-pump intermodal four wave mixing. *Opt. Express*.

[99] Koefoed J. G., Müller R. R., Rottwitt K. (2022). Mode entanglement in silicon waveguides through intermodal four wave mixing. *IEEE J. Sel. Top. Quantum Electron.*.

[100] Hoenders B. (2018). Review of a bewildering classical–quantum phenomenon: ghost imaging. *Adv. Imag. Electron. Phys.*.

[101] Michelini C., Signorini S., Pruneri V., Pavesi L. (2023). Undetected photon interference measurements on a silicon chip. *Quantum Communications and Quantum Imaging XXI*.

[102] Lahiri M., Lapkiewicz R., Lemos G. B., Zeilinger A. (2015). Theory of quantum imaging with undetected photons. *Phys. Rev. A*.

[103] Amorim R., Grüner-Nielsen L., Rottwitt K. (2025). Spatial mode conversion of single photons at the C-band using in fiber long-period gratings. *Sci. Rep.*.

[104] Mohanty A., Zhang M., Dutt A., Ramelow S., Nussenzveig P., Lipson M. (2017). Quantum interference between transverse spatial waveguide modes. *Nat. Commun.*.

[105] Lingenfelter A., Clerk A. A. (2023). Surpassing spectator qubits with photonic modes and continuous measurement for Heisenberg-limited noise mitigation. *npj Quantum Inf.*.

[106] Otte E., Nape I., Rosales-Guzmán C., Denz C., Forbes A., Ndagano B. (2020). High-dimensional cryptography with spatial modes of light: tutorial. *J. Opt. Soc. Am. B*.

[107] Wang Q., Liu J., Lyu D., Wang J. (2024). Ultrahigh-fidelity spatial mode quantum gates in high-dimensional space by diffractive deep neural networks. *Light: Sci. Appl.*.

[108] Barreto Lemos G., Lahiri M., Ramelow S., Lapkiewicz R., Plick W. N. (2022). Quantum imaging and metrology with undetected photons: tutorial. *J. Opt. Soc. Am. B*.

[109] Fadel M., Yadin B., Mao Y., Byrnes T., Gessner M. (2023). Multiparameter quantum metrology and mode entanglement with spatially split nonclassical spin ensembles. *New J. Phys.*.

[110] Vainio M., Halonen L. (2016). Mid-infrared optical parametric oscillators and frequency combs for molecular spectroscopy. *Phys. Chem. Chem. Phys.*.

[111] Haas J., Mizaikoff B. (2016). Advances in mid-infrared spectroscopy for chemical analysis. *Ann. Rev. Anal. Chem.*.

[112] Fiddler M. N., Begashaw I., Mickens M. A., Collingwood M. S., Assefa Z., Bililign S. (2009). Laser spectroscopy for atmospheric and environmental sensing. *Sensors*.

[113] De Bruyne S., Speeckaert M. M., Delanghe J. R. (2018). Applications of mid-infrared spectroscopy in the clinical laboratory setting. *Crit. Rev. Clin. Lab. Sci.*.

[114] Bogomolov A. (2015). Development and testing of mid-infrared sensors for in-line process monitoring in biotechnology. *Sensor. Actuator. B Chem.*.

[115] Botez D., Belkin M. A. (2023). *Mid-Infrared and Terahertz Quantum Cascade Lasers*.

[116] Skauli T. (2003). Improved dispersion relations for GaAs and applications to nonlinear optics. *J. Appl. Phys.*.

[117] Papatryfonos K. (2021). Refractive indices of MBE-grown Al_x_Ga_(1−x)_As ternary alloys in the transparent wavelength region. *AIP Adv.*.

[118] Anderson D., Boyd J. (1971). Wideband CO_2_ laser second harmonic generation phase matched in GaAs thin-film waveguides. *Appl. Phys. Lett.*.

[119] Fiore A., Berger V., Rosencher E., Bravetti P., Nagle J. (1998). Phase matching using an isotropic nonlinear optical material. *Nature*.

[120] Abolghasem P., Kang D., Logan D. F., Lungwitz M., Helmy A. S. (2014). Widely tunable frequency conversion in monolithic semiconductor waveguides at 2.4 μm. *Opt. Lett.*.

[121] Logan D. F., Giguere M., Villeneuve A., Helmy A. S. (2013). Widely tunable mid-infrared generation via frequency conversion in semiconductor waveguides. *Opt. Lett.*.

[122] Stievater T. H. (2014). Mid-infrared difference-frequency generation in suspended GaAs waveguides. *Opt. Lett.*.

[123] Vitali V., Demirtzioglou I., Lacava C., Petropoulos P. (2024). Nonlinear signal processing on chip. *On-Chip Photonics*.

[124] Haines J., Gandolfi M., Franz Y., De Angelis C., Guasoni M. (2021). Mid-infrared frequency generation via intermodal difference frequency generation in AlGaAs-on-insulator waveguides. *Front. Photonics*.

[125] Madsen M. L., Ulsig E. Z., Folsach S., Godoy P. H., Stanton E. J., Volet N. (2023). Mid-infrared difference-frequency generation in AlGaAs-on-insulator waveguides. *JOSA B*.

[126] Niu Y. (2022). Research progress on periodically poled lithium niobate for nonlinear frequency conversion. *Infrared Phys. Technol.*.

[127] Brameri R. (2025). Generation of CW mid-infrared radiation in the mW power range and tuneable over 400 nm. *Opt. Express*.

[128] Mishra J. (2021). Mid-infrared nonlinear optics in thin-film lithium niobate on sapphire. *Optica*.

[129] Mishra J. (2022). Ultra-broadband mid-infrared generation in dispersion-engineered thin-film lithium niobate. *Opt. Express*.

[130] Wang C., Zhong H., Ning M., Fang B., Li L., Cheng Y. (2023). Broadband second harmonic generation in a z-cut lithium niobate on insulator waveguide based on type-I modal phase matching. *Photonics*.

[131] Hansen M. T. (2023). Efficient and robust second-harmonic generation in thin-film lithium niobate using modal phase matching. *Front. Photonics*.

[132] Du H., Zhang X., Wang L., Chen F. (2023). Highly efficient, modal phase-matched second harmonic generation in a double-layered thin film lithium niobate waveguide. *Opt. Express*.

[133] Wang C. (2017). Second harmonic generation in nano-structured thin-film lithium niobate waveguides. *Opt. Express*.

[134] Luo R., He Y., Liang H., Li M., Lin Q. (2018). Highly tunable efficient second-harmonic generation in a lithium niobate nanophotonic waveguide. *Optica*.

[135] Chakkoria J. J. (2024). Efficient poling-free wavelength conversion in thin film lithium niobate harnessing bound states in the continuum. *Laser Photonics Rev.*.

[136] Ito H., Inaba H. (1975). Phase-matched guided, optical second-harmonic generation in nonlinear ZnS thin-film waveguide deposited on nonlinear LiNbO_3_ substrate. *Opt. Commun.*.

[137] Xia Y.-J., Jia R.-Z., Yang Y.-J., Zhang D.-L., Liu J.-M., Sun W.-B. (2024). Ultra-broadband and ultra-high conversion efficiency difference frequency generation in a dual-layer thin-film LiNbO_3_ waveguide with reversed ferroelectric domain orientations. *Opt. Laser. Technol.*.

[138] Zlatanovic S. (2010). Mid-infrared wavelength conversion in silicon waveguides using ultracompact telecom-band-derived pump source. *Nat. Photonics*.

[139] Kowligy A. S. (2018). Tunable mid-infrared generation via wide-band four-wave mixing in silicon nitride waveguides. *Opt. Lett.*.

[140] Franz Y., Haines J., Lacava C., Guasoni M. (2021). Strategies for wideband light generation in nonlinear multimode integrated waveguides. *Phys. Rev. A*.

[141] Botez D., Belkin M. A. (2023). State-of-the-art mid-infrared QCLs: Elastic scattering, high CW power, and coherent-power scaling. *Mid-Infrared and Terahertz Quantum Cascade Lasers*.

[142] Wang F., Slivken S., Wu D., Lu Q., Razeghi M. (2020). Continuous wave quantum cascade lasers with 5.6 W output power at room temperature and 41% wall-plug efficiency in cryogenic operation. *AIP Adv.*.

[143] Vurgaftman I. (2015). Interband cascade lasers. *J. Phys. D: Appl. Phys.*.

[144] Anagha E. G., Jeyachitra R. K. (2022). Review on all-optical logic gates: design techniques and classifications–heading toward high-speed optical integrated circuits. *Opt. Eng.*.

[145] Chen X., Lin J., Wang K. (2023). A review of silicon-based integrated optical switches. *Laser Photonics Rev.*.

[146] Sun C. (2015). Single-chip microprocessor that communicates directly using light. *Nature*.

[147] Miller D. A. (2009). Device requirements for optical interconnects to silicon chips. *Proc. IEEE*.

[148] Richardson D. J., Fini J. M., Nelson L. E. (2013). Space-division multiplexing in optical fibres. *Nat. Photonics*.

[149] Kim J.-Y., Kang J.-M., Kim T.-Y., Han S.-K. (2006). All-optical multiple logic gates with XOR, NOR, OR, and NAND functions using parallel SOA-MZI structures: theory and experiment. *J. Lightwave Technol.*.

[150] Li Z. (2005). All-optical logic gates using semiconductor optical amplifier assisted by optical filter. *Electron. Lett.*.

[151] Chen X., Huo L., Zhao Z., Zhuang L., Lou C. (2016). Study on 100-Gb/s reconfigurable all-optical logic gates using a single semiconductor optical amplifier. *Opt. Express*.

[152] Fouskidis D. E., Zoiros K. E., Hatziefremidis A. (2020). Reconfigurable all-optical logic gates (AND, NOR, NOT, OR) with quantum-dot semiconductor optical amplifier and optical filter. *IEEE J. Sel. Top. Quantum Electron.*.

[153] Lei L., Dong J., Zou B., Wu Z., Dong W., Zhang X. (2014). Expanded all-optical programmable logic array based on multi-input/output canonical logic units. *Opt. Express*.

[154] Bogris A., Velanas P., Syvridis D. (2007). Numerical investigation of a 160-Gb/s reconfigurable photonic logic gate based on cross-phase modulation in fibers. *IEEE Photonics Technol. Lett.*.

[155] Qiu J., Sun K., Rochette M., Chen L. R. (2010). Reconfigurable all-optical multilogic gate (XOR, AND, and OR) based on cross-phase modulation in a highly nonlinear fiber. *IEEE Photonics Technol. Lett.*.

[156] Li F. (2011). All-optical XOR logic gate for 40Gb/s DPSK signals via FWM in a silicon nanowire. *Opt. Express*.

[157] Yin Z. (2014). All-optical logic gate for XOR operation between 40-Gbaud QPSK tributaries in an ultra-short silicon nanowire. *IEEE Photonics J.*.

[158] Gui C., Wang J. (2013). Simultaneous optical half-adder and half-subtracter using a single-slot waveguide. *IEEE Photonics J.*.

[159] Bogoni A., Wu X., Bakhtiari Z., Nuccio S., Willner A. E. (2010). 640 Gbits/s photonic logic gates. *Opt. Lett.*.

[160] Wang J. (2008). PPLN-based flexible optical logic AND gate. *IEEE Photonics Technol. Lett.*.

[161] Wang J., Sun J., Sun Q. (2007). Proposal for all-optical switchable OR/XOR logic gates using sum-frequency generation. *IEEE Photonics Technol. Lett.*.

[162] Vo T. D. (2011). Photonic chip-based all-optical XOR gate for 40 and 160 Gbit/s DPSK signals. *Opt. Lett.*.

[163] Eggleton B. J. (2012). Photonic chip based ultrafast optical processing based on high nonlinearity dispersion engineered chalcogenide waveguides. *Laser Photonics Rev.*.

[164] Husko C., Vo T., Corcoran B., Li J., Krauss T. F., Eggleton B. J. (2011). Ultracompact all-optical XOR logic gate in a slow-light silicon photonic crystal waveguide. *Opt. Express*.

[165] Corcoran B. (2011). Ultracompact 160 Gbaud all-optical demultiplexing exploiting slow light in an engineered silicon photonic crystal waveguide. *Opt. Lett.*.

[166] Rao D. G. S., Swarnakar S., Palacharla V., Raju K. S. R., Kumar S. (2021). Design of all-optical AND, OR, and XOR logic gates using photonic crystals for switching applications. *Photonic Netw. Commun.*.

[167] Ma M., Chen L. R. (2016). Harnessing mode-selective nonlinear optics for on-chip multi-channel all-optical signal processing. *APL Photonics*.

[168] Wang J. (2017). Dual-channel AND logic gate based on four-wave mixing in a multimode silicon waveguide. *IEEE Photonics J.*.

[169] Hu Y. (2022). 3 × 40 Gbit/s all-optical logic operation based on low-loss triple-mode silicon waveguide. *Micromachines*.

[170] Wang Y. (2020). Highly nonlinear organic-silicon slot waveguide for ultrafast multimode all-optical logic operations. *IEEE Photonics J.*.

[171] Seok T. J., Kwon K., Henriksson J., Luo J., Wu M. C. (2019). Wafer-scale silicon photonic switches beyond die size limit. *Optica*.

[172] Zhang G., Mojaver H. R., Das A., Liboiron-Ladouceur O. (2020). Mode insensitive switch for on-chip interconnect mode division multiplexing systems. *Opt. Lett.*.

[173] Yang L. (2018). General architectures for on-chip optical space and mode switching. *Optica*.

[174] Liu S., Fu X., Niu J., Huo Y., Cheng C., Yang L. (2024). Demultiplexing-free ultra-compact WDM-compatible multimode optical switch assisted by mode exchanger. *Nanophotonics*.

[175] Lüpken N. M., Hellwig T., Schnack M., Epping J. P., Boller K.-J., Fallnich C. (2018). Low-power broadband all-optical switching via intermodal cross-phase modulation in integrated optical waveguides. *Opt. Lett.*.

[176] Lin Y.-Y., Yang S.-E., Deng Y.-L., Lee C.-K., Chiu Y.-J. (2022). All-optical signal processing by nonlinear multimode interference in Ta_2_O_5_ waveguides. *ACS Appl. Opt. Mater.*.

[177] Nozaki K. (2010). Sub-femtojoule all-optical switching using a photonic-crystal nanocavity. *Nat. Photonics*.

[178] Sharping J. E., Fiorentino M., Kumar P., Windeler R. S. (2002). All-optical switching based on cross-phase modulation in microstructure fiber. *IEEE Photonics Technol. Lett.*.

[179] Schnack M., Hellwig T., Fallnich C. (2016). Ultrafast, all-optical control of modal phases in a few-mode fiber for all-optical switching. *Opt. Lett.*.

[180] Chen X., Bai R., Huang M. (2019). Optical properties of amorphous Ta_2_O_5_ thin films deposited by RF magnetron sputtering. *Opt. Mater.*.

[181] Ye Z. (2023). Foundry manufacturing of tight-confinement, dispersion-engineered, ultralow-loss silicon nitride photonic integrated circuits. *Photonics Res.*.

[182] Kuznetsov N. (2024). An integrated gallium phosphide travelling-wave optical parametric amplifier. *Optical Fiber Communication Conference*.

[183] Ranka J. K., Windeler R. S., Stentz A. J. (2000). Visible continuum generation in air–silica microstructure optical fibers with anomalous dispersion at 800 nm. *Opt. Lett.*.

[184] Cristiani I., Tediosi R., Tartara L., Degiorgio V. (2004). Dispersive wave generation by solitons in microstructured optical fibers. *Opt. Express*.

[185] Husakou A. V., Herrmann J. (2001). Supercontinuum generation of higher-order solitons by fission in photonic crystal fibers. *Phys. Rev. Lett.*.

[186] Kou R. (2023). Spatially resolved multimode excitation for smooth supercontinuum generation in a SiN waveguide. *Opt. Express*.

[187] Roy S., Bhadra S. K., Agrawal G. P. (2009). Effects of higher-order dispersion on resonant dispersive waves emitted by solitons. *Opt. Lett.*.

[188] Epping J. P. (2015). On-chip visible-to-infrared supercontinuum generation with more than 495 THz spectral bandwidth. *Opt. Express*.

[189] Jia J., Kang Z., Huang Q., He S. (2022). Mid-infrared highly efficient, broadband, and flattened dispersive wave generation via dual-coupled thin-film lithium-niobate-on-insulator waveguide. *Appl. Sci.*.

[190] Fan R. (2021). Higher order mode supercontinuum generation in tantalum pentoxide (Ta_2_O_5_) channel waveguide. *Sci. Rep.*.

[191] Chen H. (2021). Supercontinuum generation in high order waveguide mode with near-visible pumping using aluminum nitride waveguides. *ACS Photonics*.

[192] Hickstein D. D. (2018). Quasi-phase-matched supercontinuum generation in photonic waveguides. *Phys. Rev. Lett.*.

[193] Grassani D. (2019). Mid infrared gas spectroscopy using efficient fiber laser driven photonic chip-based supercontinuum. *Nat. Commun.*.

[194] Montesinos-Ballester M. (2020). On-chip mid-infrared supercontinuum generation from 3 to 13 μm wavelength. *ACS Photonics*.

[195] Lu Q., Wu D., Sengupta S., Slivken S., Razeghi M. (2016). Room temperature continuous wave, monolithic tunable THz sources based on highly efficient mid-infrared quantum cascade lasers. *Sci. Rep.*.

[196] Xia L., van der Slot P., Timmerkamp M., Bastiaens H., Fallnich C., Boller K.-J. (2023). On-chip phase-shift induced control of supercontinuum generation in a dual-core Si_3_N_4_ waveguide. *Opt. Express*.

[197] Xia L., van der Slot P. J., Toebes C., Boller K.-J. (2024). Physical mode analysis of multimode cascaded nonlinear processes in strongly-coupled waveguides. ..

[198] Xia L., van der Slot P., Timmerkamp M., Fallnich C., Boller K.-J. (2024). Transverse-mode nonlinear interactions in strongly coupled integrated waveguides. *Frontiers in Optics*.

[199] Fatema S., Mia M. B., Kim S. (2021). Multiple mode couplings in a waveguide array for broadband near-zero dispersion and supercontinuum generation. *J. Lightwave Technol.*.

[200] Guo H. (2020). Nanophotonic supercontinuum-based mid-infrared dual-comb spectroscopy. *Optica*.

[201] Yulin A., Skryabin D. V., Vladimirov A. G. (2006). Modulational instability of discrete solitons in coupled waveguides with group velocity dispersion. *Opt. Express*.

[202] Królikowski W., Kivshar Y. S. (1996). Soliton-based optical switching in waveguide arrays. *J. Opt. Soc. Am. B*.

[203] Shi M., Ribeiro V., Perego A. M. (2023). Parametric amplification in coupled nonlinear waveguides: the role of coupling dispersion. *Front. Photonics*.

[204] Ribeiro V., Perego A. M. (2022). Parametric amplification in lossy nonlinear waveguides with spatially dependent coupling. *Opt. Express*.

[205] Yuan J. (2015). Enhanced intermodal four-wave mixing for visible and near-infrared wavelength generation in a photonic crystal fiber. *Opt. Lett.*.

[206] Lüpken N. M. (2021). Numerical and experimental demonstration of intermodal dispersive wave generation. *Laser Photonics Rev.*.

[207] Dudley J. M., Coen S. (2002). Coherence properties of supercontinuum spectra generated in photonic crystal and tapered optical fibers. *Opt. Lett.*.

[208] Liu L. (2016). Coherent mid-infrared supercontinuum generation in all-solid chalcogenide microstructured fibers with all-normal dispersion. *Opt. Lett.*.

[209] Cheng Y. (2020). Mid-infrared supercontinuum and frequency comb generations by different optical modes in a multimode chalcogenide strip waveguide. *IEEE Access*.

[210] Niu S. (2022). Research progress of monolithic integrated DFB laser arrays for optical communication. *Crystals*.

[211] Li N. (2022). Integrated lasers on silicon at communication wavelength: a progress review. *Adv. Opt. Mater.*.

[212] Liu D.-S., Wu J., Xu H., Wang Z. (2021). Emerging light-emitting materials for photonic integration. *Adv. Mater.*.

[213] Van Gasse K. (2019). Recent advances in the photonic integration of mode-locked laser diodes. *IEEE Photonics Technol. Lett.*.

[214] Cao V. (2022). Recent progress of quantum dot lasers monolithically integrated on Si platform. *Front. Phys.*.

[215] Stone J. R., Lu X., Moille G., Srinivasan K. (2022). Efficient chip-based optical parametric oscillators from 590 to 1150 nm. *APL Photonics*.

[216] Jornod N. (2023). Monolithically integrated femtosecond optical parametric oscillators. *Optica*.

[217] Fortier T., Baumann E. (2019). 20 years of developments in optical frequency comb technology and applications. *Commun. Phys.*.

[218] Ferrara M. A., Sirleto L. (2020). Integrated Raman laser: a review of the last two decades. *Micromachines*.

[219] Yang Q.-F., Yi X., Yang K. Y., Vahala K. (2017). Stokes solitons in optical microcavities. *Nat. Phys.*.

[220] Yang Q.-F., Yi X., Yang K. Y., Vahala K. J. (2017). Stokes solitons in optical microcavities. *Conference on Lasers and Electro-Optics*.

[221] Lucas E. (2018). Spatial multiplexing of soliton microcombs. *Nat. Photonics*.

[222] Lucas E. (2018). Data and code for figures: spatial multiplexing of soliton microcombs [data set]. In spatial multiplexing of soliton microcombs. ..

[223] Bao C. (2019). Orthogonally polarized frequency comb generation from a Kerr comb via cross-phase modulation. *Opt. Lett.*.

[224] Kamel A. N. (2019). Generation of clustered frequency comb via intermodal four-wave mixing in an integrated Si_3_N_4_ microresonator. *2019 Conference on Lasers and Electro-Optics (CLEO)*.

[225] Weng H. (2022). Dual-mode microresonators as straightforward access to octave-spanning dissipative Kerr solitons. *APL Photonics*.

[226] Zhang Y., Zhong K., Hu G., Yi D., Kumar R. R., Tsang H. K. (2020). Sub-milliwatt optical frequency combs in dual-pumped high-Q multimode silicon resonators. *Appl. Phys. Lett.*.

[227] Zhang Y., Hu G., Zhong K., Zhou W., Tsang H. K. (2021). Investigation of low-power comb generation in silicon microresonators from dual pumps. *J. Opt.*.

[228] Cui S., Cao K., Pan Z., Gao X., Yu Y., Zhang X. (2023). Compact microring resonator based on ultralow-loss multimode silicon nitride waveguide. *Adv. Photonics Nexus*.

[229] Ji X. (2021). Exploiting ultralow loss multimode waveguides for broadband frequency combs. *Laser Photonics Rev.*.

[230] Zhang Y., Zhong K., Tsang H. K. (2021). Raman lasing in multimode silicon racetrack resonators. *Laser Photonics Rev.*.

[231] Ye C. (2024). Multimode AlGaAs-on-insulator microring resonators for nonlinear photonics. *IEEE J. Sel. Top. Quantum Electron.*.

[232] Zhang Y., Zhong K., Tsang H. K. (2023). Compact multimode silicon racetrack resonators for high-efficiency tunable Raman lasers. *Appl. Phys. Lett.*.

[233] Skirlo S. A., Lu L., Soljačić M. (2014). Multimode one-way waveguides of large Chern numbers. *Phys. Rev. Lett.*.

[234] Blanco-Redondo A. (2016). Topological optical waveguiding in silicon and the transition between topological and trivial defect states. *Phys. Rev. Lett.*.

[235] Blanco-Redondo A., Bell B., Oren D., Eggleton B. J., Segev M. (2018). Topological protection of biphoton states. *Science*.

